# New Docking, Molecular Dynamics, and QSAR Models to Predict Disruption of Human and Rat Transthyretin Function by Per- and Polyfluoroalkyl Substances (PFAS)

**DOI:** 10.1021/acs.chemrestox.5c00424

**Published:** 2026-04-27

**Authors:** Nuno M. S. Almeida, Heather M. Bolstad, Scott Coffin, Sana Majid, Angela K. Wilson, Anatoly A. Soshilov

**Affiliations:** † New Toxicology Evaluations Section (NTES), Office of Environmental Health Hazard Assessment, 7020 California Environmental Protection Agency, 1001 I St, Sacramento, California 95814, United States; ‡ Department of Chemistry, 3078 Michigan State University, East Lansing, Michigan 48864, United States

## Abstract

Per- and polyfluoroalkyl substances (PFAS) are environmentally persistent chemicals that require an improved understanding of the toxicity mechanisms and the development of predictive models for risk assessment. One observed effect of PFAS exposure is a decrease in thyroxine (T_4_) levels *in vivo* resulting from the direct displacement of T_4_ from a carrier protein, transthyretin (TTR), in a proposed adverse outcome pathway (AOP). In this study, the mechanism of thyroxine (T_4_) displacement from human and rat TTRs was investigated by using structural approaches (i.e., docking and molecular dynamics) and quantitative structure–activity relationship (QSAR) models. A QSAR model was developed using the largest available binding data set and a two-tier approach that allowed inclusion of all data. Docking models that utilized a pharmacophore approach showed nearly perfect overlap with independently sourced crystal structures for perfluorooctanoic acid (PFOA) and perfluorooctanesulfonic acid (PFOS). Molecular dynamics simulations demonstrated similar PFAS binding modes in rat and human TTR, enabling interspecies toxicity comparisons. All models predicted moderate to strong binding of the novel PFAS 4,8-Dioxa-3*H*-perfluorononanoic acid (ADONA) and hexafluoropropylene oxide dimer acid (GenX) to TTR, consistent with the limited toxicity and binding data for these chemicals. Predicted PFAS binding energies for rat TTR correlated well with the *in vivo* PFAS-associated decreases in T_4_ levels, supporting the AOP. The development of reliable predictive toxicity models for PFAS requires extensive validation, maximal use of available experimental data, and careful consideration of toxicokinetic differences in interchemical comparisons.

## Graphical abstract

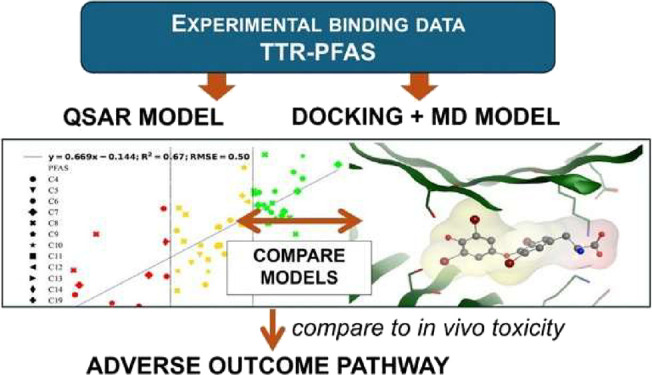

## Introduction

Per- and polyfluoroalkyl substances are hazardous, persistent environmental pollutants with documented adverse effects in humans and animals. Despite growing concerns, PFAS continue to be widely used in industrial processes, firefighting foams, and in common household products.^1,2^ The resistance of PFAS to environmental degradation stems from their fluorine saturation and the strength of the carbon–fluorine bonds. Due to their widespread use and biological persistence, PFAS pose a significant health risk; they are reported to be present in more than 99% of the U.S. population.^3–8^

Health risk assessments have been conducted for certain PFAS and mixtures with sufficient toxicological data.^9–11^ However, many PFAS of potential concern have not been evaluated due to limited *in vivo* data necessary for the establishment of health-protective standards. Additionally, new PFAS are regularly introduced as alternatives, often without comprehensive toxicity profiles.^12^ This underscores the need for predictive approaches to assess PFAS toxicity.^13–16^

In mammals, exposure to PFAS has been associated with a wide variety of adverse outcomes such as cancer, hepatotoxicity, immunotoxicity, developmental toxicity, and thyroid toxicity, among the most reported.^17,18^ While epidemiological PFAS studies generally support many of the observations in animal models, it is difficult to isolate the effects of individual PFAS in multichemical environmental exposures.^19,20^ Therefore, animal models have had an important role in health risk assessment, serving both as a foundation for understanding human effects and as an aid in the development and validation of predictive tools for PFAS toxicity. However, the ever-expanding list of PFAS of concern, limited available animal data, and the overall proposed shift away from animal research toward alternative toxicity models make the development of predictive in silico models imperative in the PFAS health risk assessment.

Thyroid toxicity caused by PFAS is an important example of this framework. Decreases in triiodothyronine (T_3_) and thyroxine (T_4_) in rat studies have been identified as the most sensitive adverse effects for certain short-chain PFAS—perfluorobutanesulfonic acid (PFBS), perfluorobutanoic acid (PFBA), perfluorohexanoic acid (PFHxA), and perfluorohexanesulfonic acid (PFHxS)—and these findings have informed human health-protective concentrations in recent health assessments.^21–35^ For longer-chain PFAS, such as perfluorooctanoic acid (PFOA), perfluorooctanesulfonic acid (PFOS), perfluorononanoic acid (PFNA), and perfluorodecanoic acid (PFDA), hepatic or immune effects appear to be more sensitive indicators of toxicity than thyroid effects, suggesting end point-specific trends within this chemical group.^2,9,10,17,24,25,27–36^ Epidemiological data for PFAS typically do not show consistent changes in thyroid hormone levels, although decreases in T_3_/T_4_ have been reported in occupational settings.^37–40^ The differences between available animal and human data could be due to species (e.g., toxicokinetic) differences and different levels of exposure in the animal (higher) and human epidemiological (lower) studies. Specifically, species and chemical toxicokinetic differences among PFAS can be quite drastic;^41^ these are rarely accounted for in model development due to limited data.

*In vivo* T_3_ and T_4_ decreases have been proposed to result from increased degradation following the PFAS-dependent displacement from the T_3_/T_4_ transport protein transthyretin (TTR). This mechanism has been proposed as an AOP for the neurodevelopmental toxicity of xenobiotics (AOP 152 at aopwiki.org).^42^

*In vitro* ability of PFAS to displace T_4_ or related probes has been intensively investigated with human TTR;^43–49^ however, no *in vitro* studies are available with rat TTR. Most recently, Degitz *et al.* examined 136 PFAS with a panel of nine *in vitro* assays targeting various thyroid toxicity mechanisms and found that binding to human TTR was the most common (i.e., had the highest number of active compounds) and most sensitive *in vitro* end point among those analyzed.^45^ In this and related studies, the ability of PFAS to displace T_4_ from TTR is quantified using half maximal effective concentration (EC_50_) or half maximal inhibitory concentration (IC_50_) in a ligand binding assay, employing a radioactive or fluorescent high-affinity ligand probe (distinct from the endogenous T_4_). Of note, Degitz *et al.* reported three PFAS-PFHxS, PFHpS, and (perfluorobutyryl)-2-thenoylmethane with higher affinity for TTR than that of T_4_.^45^ PFAS binding to hTTR was also investigated with mutagenesis, demonstrating the critical role of Lys15.^44^

In silico investigations into the mechanism of T_4_ displacement by PFAS primarily involved docking models and quantitative structure–activity relationship (QSAR) models. Docking studies based on crystal structures of PFAS with TTR have been reported.^50,51^ Furthermore, such docking models have been used to conduct in silico mutagenesis of TTR and to investigate species and PFAS differences in TTR-PFAS binding.^50,52–54^ For example, Zhang *et al.* conducted virtual mutagenesis of hTTR to investigate how a small set of thyroid-disrupting chemicals, including PFOA, interacted differently with seabream TTR and hTTR.^50^ In this study, Ser117 was identified as potentially important for binding polar chemicals (but not PFOA); however, no experimental validation of this prediction was provided.^50^ In another recent study, binding energies predicted from molecular dynamics simulations of TTR-PFAS were used to develop a QSAR model for 430 PFAS.^55^ Dharpure *et al.* analyzed species-specific PFAS binding to TTR and found that many key binding residues (including Lys15) are conserved among species.^53^ This study also suggested that long-chain PFAS (≥6 carbons) tend to bind TTR more efficiently than short-chain PFAS (<6 carbons).

Several QSAR models for hTTR-PFAS interactions have been developed based on experimental direct binding data.^43,49,55–59^ Notably, almost all published QSAR models for direct PFAS binding are based on the same TTR-PFAS binding data set from Weiss *et al.* ^43,55^ A key limitation of this data set is its small size (*N* = 24 PFAS), which limits the predictive power of the QSAR models. As mentioned above, more comprehensive and structurally diverse data sets have recently become available,^45,47^ potentially enabling more comprehensive QSAR models. Thus, Evangelista *et al.* utilized the TTR-PFAS binding data set from Degitz *et al.*, which is the largest data set available to date, to build a multilinear regression QSAR model.^45^

While PFAS-T_4_ displacement from hTTR has been extensively studied *in vitro* and in silico,^43–49,58^ no animal PFAS-T_4_/TTR *in vitro* displacement data have been reported to date. This represents an important knowledge gap, especially since the more conclusive *in vivo* data on PFAS-induced thyroid toxicity are from animal (rat) studies. Buglewicz *et al.* used structural comparisons and molecular dynamics simulations of PFOA to understand species differences in binding to TTR.^60^ The authors found that PFOA-TTR binding interactions are generally conserved in vertebrate groups. Thus, the clear need for predictive models of PFAS thyroid toxicity faces several challenges: (i) models need to incorporate observations for the highest number of chemicals possible and clearly explore the applicability aspects; (ii) there is a need to compare among multiple existing and developed models, selecting not only better performing but also most informative and relevant models, and such relevance criteria need to be clearly defined; and (iii) at least two paramount extrapolation challenges need to be explicitly addressed, including the species data gap (*in vitro* data in human systems; *in vivo* toxicity data in the rat) and toxicokinetic adjustments in model validation experiments. While select cited studies described above include some of these aspects, the overall pragmatic and detail-exacting framework has not been reported.

Developing such a framework was the purpose of this study, with the overall focus on investigating the PFAS-mediated displacement of T_4_ from TTR as a molecular mechanism and as a basis for the AOP for PFAS-induced T_4_ reduction in rats. Molecular docking and QSAR models were developed for binding of PFAS to hTTR, with maximum use of multiple types of available experimental data for model development and validation. The validated hTTR-PFAS models were used to predict binding affinities for two novel PFAS that were not analyzed in the hTTR binding data set (ADONA and GenX) as an example of framework application. To account for interspecies differences in model development and toxicity extrapolations, a rat TTR (rTTR) docking model was developed, and molecular dynamics simulations were used to compare predicted PFAS binding energies between the hTTR and rTTR models. Finally, to investigate the role of PFAS/T_4_ displacement as a key molecular interaction underpinning *in vivo* T_4_ reduction in male rats (AOP), predicted rTTR-PFAS binding energies were compared to toxicokinetically standardized *in vivo* points of departure (PODs) for PFAS-dependent T_4_ decreases. A summary of the study design is shown in Figure 1. This graphic includes different experimental data sources used, models that were developed and validated by using experimental data, and the predictions generated from these models.

**Figure 1:**
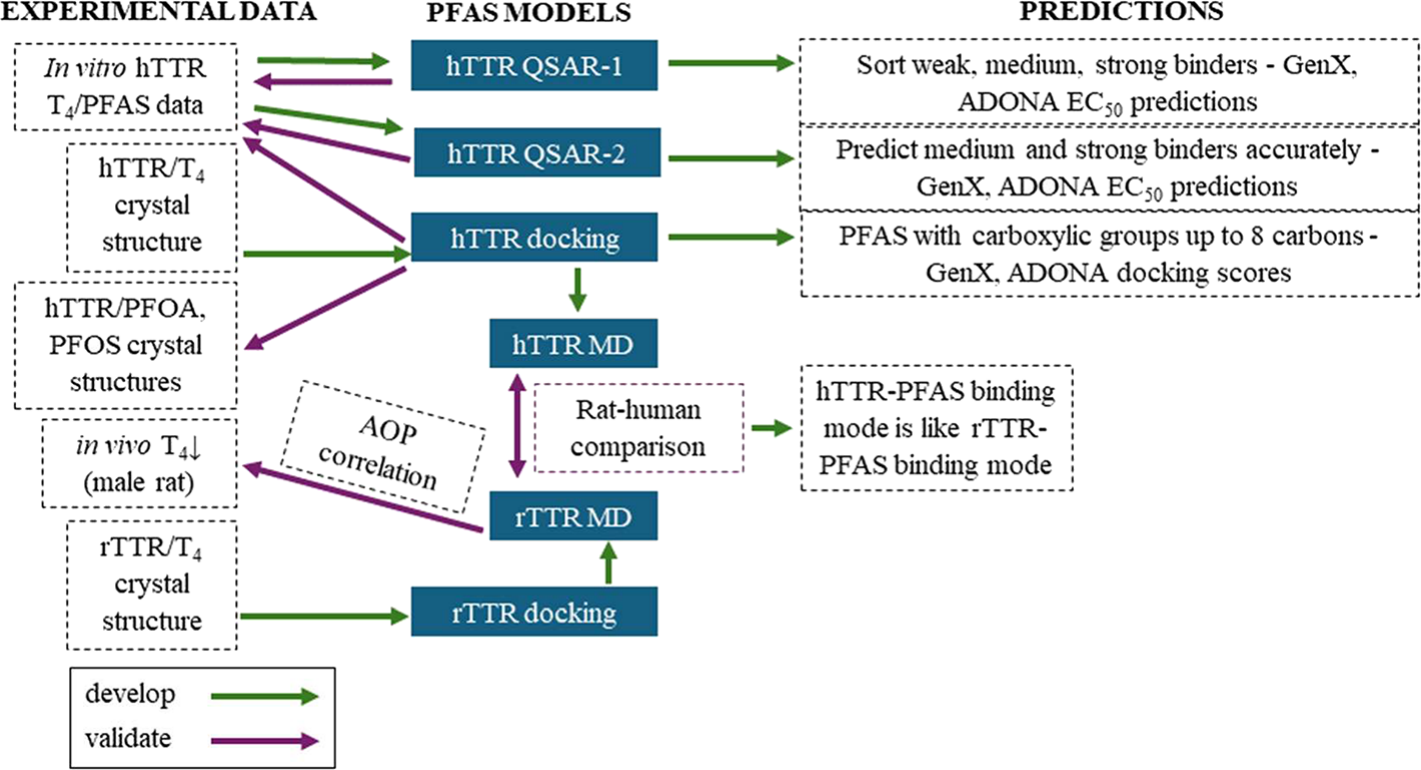
Project outline. Abbreviations: h, human; r, rat; TTR, transthyretin; T_4_, thyroxine; AOP, adverse outcome pathway; EC_50_, effective concentration at half-maximum activity; MD, molecular dynamics.

## Methodology

### hTTR and rTTR QSAR Models

Five *in vitro* dose–response studies reporting T_4_ displacement from TTR were identified using PubMed searches.^43–45,47,48^ Binding data from these are summarized in Figure 2 (only a representative selection of PFAS from Degitz *et al.* ^45^ is included due to the large size of this data set). To develop relative binding values (−log_10_ EC_50_/EC_50_^PFOA^) for this graph, EC_50_ values from corresponding sources were converted to μM. The red vertical line corresponds to a binding affinity similar to that of PFOA. The data set of Degitz *et al.* (Table S1) was chosen for model development since it had the largest number of active PFAS (*n* = 80).

**Figure 2:**
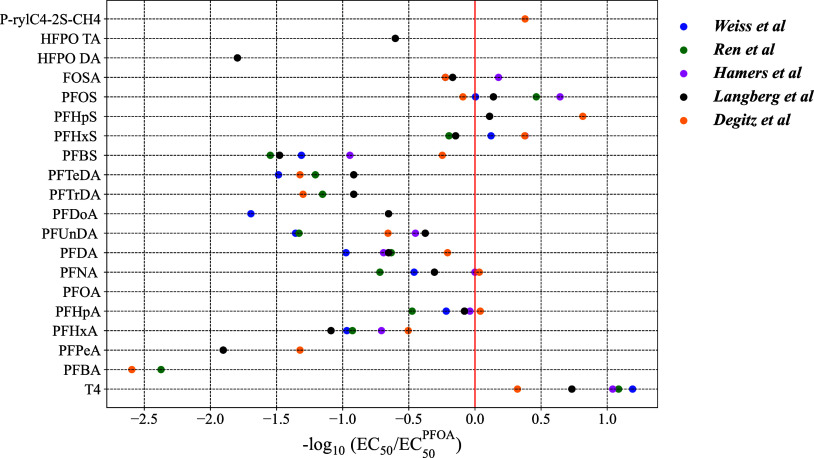
*In vitro* TTR displacement data. EC_50_ values for each PFAS were transformed into normalized binding activities and plotted on the *x*-axis. Data are from Weiss *et al.*,^43^ Ren *et al.*,^44^ Hamers *et al.*,^48^ Langberg *et al.* ^47^ and Degitz *et al.* ^45^ Only select representative data points from Degitz *et al.* are included, and the full data set can be found in the Supporting Information.

Several quantitative structure–activity relationship (QSAR) models were constructed based on the data set from Degitz *et al.* using partial least-squares (PLS) in Molecular Operating Environment (MOE 2022) (equations for the models are provided in the SI).^61^

3D molecular conformations of ligands were optimized in MOE by using the AMBER 10 Extended Hückel Theory (EHT) force field.^62,63^ Molecular and physicochemical descriptors (2D and 3D) were generated using MOE 2022. Optimization on the full data set (80/20 split, as described below) and selection of the most significant descriptors led to QSAR Model 1. The model optimization strategy involved increasing the goodness of fit (*R* ^2^), decreasing the gap between the model *R* ^2^ and cross-validation *R* ^2^, and minimizing the total number of descriptors for equal model performance. Further investigating QSAR Model 1, we also noted that limiting the PFAS set to medium- and high-affinity binders allowed development of a better performing QSAR model, even though the training/test sets were smaller. Further selection of the most significant descriptors for this approach led to QSAR Model 2. In the end, these two QSAR models, demonstrating the best fit (highest *R* ^2^) with different applications (as described below), were chosen for further evaluation in a tiered approach. The main difference between the two models is that QSAR Model 1 applies to all studied PFAS with a somewhat higher prediction error (RMSE), while QSAR Model 2 targets medium- and high-affinity PFAS with a lower prediction error (RMSE). For QSAR Model 1, the selected descriptors (i.e., ten most significant in optimizations with the full descriptor set) included the number of carbons (n_C), the number of atoms (a_count), the number of single bonds (b_single), Topological Polar Surface Area (TPSA), Balaban J Index (BalabanJ), number of oxygens (a_no), Lipinski violation rules (lip_violation), GCUT descriptors using atomic contribution to logP (GCUT_SLOP_3), and first kappa alpha modified shape index (KierA1). For QSAR Model 2, the ten selected descriptors (i.e., ten most significant in optimizations with the full descriptor set) were very similar to Model 1’s list, with one additional descriptor—van de Waals energy (E_vdw) on the list, at the expense of the KierA1 descriptor, which was no longer included.

For QSAR Model 1, a training set containing 62 PFAS structures and a test set containing 10 PFAS structures were created using an 80/20 stratified split from the Degitz *et al.* data set with the aim of including a representative range of chemical structures and pEC_50_ values (Table S2). The pEC_50_ values for the training set ranged from 1.17 to −2.62 and covered weak (pEC_50_ < −0.9), medium (−0.9 ≤ pEC_50_ ≤ 0.12), and strong (0.12 < pEC_50_) binders. The test set contained ten PFAS, including weak, medium, and strong binders (Table S2). For QSAR Model 2, the training set containing 32 PFAS (with pEC_50_ values ranging from 1.17 to 0.16) and the test set containing eight PFAS were created using an 80/20 stratified split with the aim of including medium and strong binders in either set (Table S3).

The domain of applicability (DoA) for the QSAR models was analyzed using principal component analysis (PCA) in MOE 2022.^60^ PCA components were generated for QSAR Model 1 and QSAR Model 2. PCA values for GenX and ADONA were calculated and plotted alongside PCA1 and PCA2 values from the model training sets, including variance. ADONA and GenX were found to be within the DoA for both QSAR models. Accordingly, the predicted pEC_50_ values for these compounds were generated in both QSAR models.

### hTTR and rTTR Docking Models

Docking simulations were performed with MOE 2022.^61^ Crystal structures of human and rat TTR were obtained from the RSCB Protein Data Bank (PDB IDs: 1ICT and 1IE4, respectively).^64,65^ *In vivo*, TTR has a homotetrameric structure with T_4_ binding sites seemingly located in the interface or interfaces between monomers. The hTTR crystal structure (1ICT) is composed of a tetrameric form of TTR cocrystallized with T_4_, therefore enabling a full structural analysis of the potential binding sites.^36^ The rTTR structure (1IE4) includes T_4_ bound in two distinct binding pockets (Figure S1A).^37^ For both species, binding pocket 1 was selected for model development (Figure 2 A,C); the rationale for this selection is detailed in the Molecular Dynamics Simulations section below. Prior to docking, protein structures were protonated at pH = 7, 300K and 1 atm, utilizing the protonate3D approach in MOE 2022.^61,66^

For docking to the hTTR, 72 PFAS were selected from the Degitz *et al.* data set^45^ and all PFAS salt forms were removed. Thus, the following compounds were not included: ammonium perfluorooctanoate, potassium perfluorooctanoate, potassium perfluorooctanesulfonate, potassium perfluorohexanesulfonate, sodium perfluorooctanoate, potassium perfluorobutanesulfonate, and perfluorooctanesulfonamide ammonium iodide. One duplicate was removed (1,6-Diiodoperfluorohexane), and the lower EC_50_ value (1.71 μM) was retained. Only linear isomers of PFAS were considered. PFAS were deprotonated at pH 7 and 300 K.

For the rTTR, a targeted subset of 10 PFAS and T_4_ was docked to allow comparison with the hTTR binding data and corresponding *in vivo* toxicity data. In both structures, the binding site was selected using MOE’s “site finder” and encompassed residues within a 5 Å range of the cocrystallized T_4_ ligand. The induced fit approach was used for docking with the London Δ*G* scoring function, followed by the generalized-born volume integral/weighted surface area scoring function (GBVI/WSA Δ*G*, energy refinement function), which provided 10 refined poses.^67^ The selected ligand binding pocket 1 for human and rat TTRs is shown in Figure 3A,C, respectively, highlighting key protein residue interactions. One such residue, Lys15, has previously been identified by Ren *et al.* as critical for ligand binding.^44^ In this study, Lys15 mutations led to significantly weaker binding affinities for all of the studied PFAS and T_4_. Pharmacophore models for the T_4_ interaction with human and rat TTRs were developed on the basis of the importance of Lys15 and considering the structural features of 1ICT and 1IE4 (Figure 3B,D). These pharmacophore models were then integrated into the docking protocols with an essential hydrogen bonding acceptor feature to Lys15, with radii of 1.8 and 1.6 Å, for the human and rat, respectively. Additional details on the docking protocol are provided in the SI (Methodology, hTTR and rTTR docking models).

**Figure 3:**
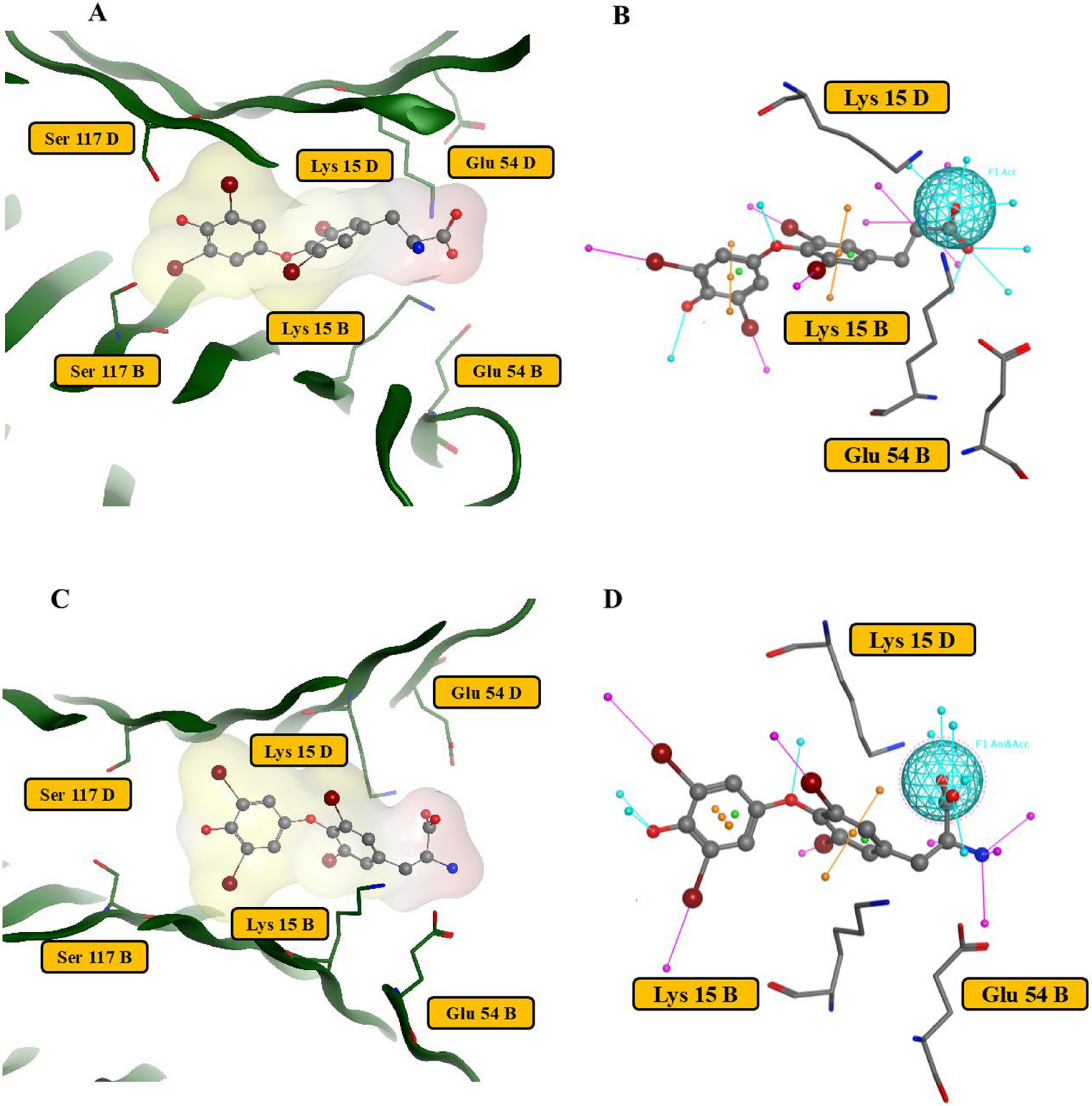
Binding pockets of the crystal structures of human (3A) and rat (3C) cells with T_4_. T_4_-binding fragments of hTTR (3A) and rTTR (3C) are shown for binding pocket 1. The T_4_ surface is indicated and marked yellow for lipophilic areas and red for hydrophilic areas. The pharmacophore models utilized for hTTR and rTTR docking models are shown (3B and 3D, respectively).

### Molecular Dynamics Simulations

Molecular dynamics simulations were performed for selected PFAS using the relevant docking poses in human and rat TTR models with Amber22.^68^ Simulations were run for 10 PFAS and T_4_, each for 100 ns, with a 2 fs time step. To evaluate the stability and strength of T_4_ binding to TTR, simulations were done in triplicate in both human and rat models. These simulations assessed T_4_ positional dynamics and potential interactions within the binding pocket (further details of the simulations are provided in the (Methodology—Molecular dynamics simulations).

As previously noted, the rat crystal structure (1IE4) contains two distinct binding pockets, each with distinct residue interactions. To investigate possible differences in binding behavior, both pockets were initially simulated. However, no noticeable differences in binding affinity were observed between the two; hence, only binding pocket 1 was used for subsequent PFAS-TTR simulations in the rat model. For human TTR, binding pocket 1 was also selected, as T_4_ binding poses were found to be consistent across both sites.

Binding energies were calculated using the molecular mechanics Poisson–Boltzmann surface area (MM-PBSA) method.^69^ The full simulation trajectory was analyzed with 51 frames sampled per 10 ns time step. Each frame was analyzed individually, and the binding energy results were averaged. CPPTRAJ was used for the residue decomposition step analysis.^70^

### *In Vivo* Toxicity Data Analysis

PFAS rat toxicology literature was reviewed to identify studies reporting thyroid hormone measurements. Our analysis focused on 28-day oral gavage studies conducted by the National Toxicology Program (NTP), which encompassed a wide range of PFAS chain lengths and used standardized methodologies, thereby reducing interstudy variability and facilitating direct comparison of results.^24,25^ The NTP evaluated the following perfluoroalkylcarboxylic acids (PFCAs): PFHxA, PFOA, PFNA, and PFDA, along with the following perfluoroalkylsulfonic acids (PFSAs): PFBS, PFHxS, and PFOS. In addition, a separate 28-day oral gavage study in rats conducted with PFBA was identified.^21^ Thyroid toxicity end points from selected studies were reviewed, and data sets on free and total T_4_ decreases were chosen for inclusion in this analysis. These end points were prioritized due to their widespread reporting across the selected PFAS studies and the mechanistic considerations of the model (TTR specifically binds T_4_). Applied doses in these data sets were converted to serum concentrations (see below) and dose–response data were modeled in ToxicR (version 24.10.1.4)^71^ to derive benchmark dose (BMD) values using the default suite of continuous models and the Bayesian model average option. In this analysis, a BMR of one standard deviation was used. Consistent with regulatory practice, the corresponding BMDL values would be used as the points of departure (PODs) for subsequent risk assessment. A single-compartment, first-order toxicokinetic model (with or without an absorption phase), or a physiologically based pharmacokinetic (PBPK) model, was used to model serum concentrations at 1 h time-point increments, with model choice based on parameter data availability. Sex-specific rat toxicokinetic parameters (including elimination half-life, volume of distribution, and absorption rate constant) for each PFAS were obtained from the literature (Table S4). Serum maximum concentration (*C*_max_) and time-weighted average concentrations (TWA) were estimated using each toxicology study’s modeled time-series data. Additional details about the models are included in the Supporting Information (Toxicokinetic modeling).

### Bayesian Regression

To investigate the relationship between rat TTR binding energy and BMD metrics, a Bayesian regression model was implemented using the BMRS package (2.22)^72^ in R (4.4.1), with separate analyses for dose metrics. The model incorporated measurement errors in both the predictor (rTTR binding energy; kcal mol^–1^) and the response variable (*in vivo* BMD PODs; μM), with prior knowledge informed by frequentist regression results. Specifically, the slope and intercept priors were approximated as normal distributions, centered on the frequentist estimates with standard deviations equal to the standard deviation for the predictor and geometric standard error for the response variable. A weakly informative Cauchy prior was applied to the residual standard deviation. Posterior samples were drawn using Markov Chain Monte Carlo methods, and posterior predictive checks were performed to evaluate model fit. Model performance metrics, including root-mean-squared error (RMSE) and Bayesian R-squared, were calculated to assess predictive accuracy and model quality.

## Results and Discussion

Three predictive approaches were used to assess the binding of PFAS to hTTR: QSAR, docking, and molecular dynamics simulations. For the binding of PFAS to rTTR, docking and molecular dynamics simulations were used. The PFAS-hTTR QSAR model was developed in two tiers: Tier 1 predicted binding across all PFAS, while Tier 2 provided refined predictions for medium and strong binders.

### QSAR Models for TTR

For the hTTR-PFAS model, the pEC_50_ values from the Degitz *et al.* data set provided an appropriate benchmark for QSAR predictions. The developed QSAR models allow for predictions of PFAS-hTTR binding. Model 1 facilitates the understanding of the binding region of any PFAS, while Model 2 predicts strong and medium binders with a smaller root-mean-square error (RMSE). These models provide a wide range of structural features for use in the structure–activity relationships of different PFAS (see Figures 4 and 5), including carboxylic and sulfonic acids, alcohols, aliphatic, and nonaliphatic structures. GenX and ADONA were also evaluated using both QSAR models, and their pEC_50_ were calculated (Figure 6 shows QSAR predictions for GenX and ADONA, along with calculated docking scores). A comparison summary of prior QSAR modeling based on the Weiss *et al.* data from 2009 ^43,56–59^ was performed (Table S5). The most recent PFAS-TTR QSAR model (Zhao *et al.* ^55^) used the Degitz *et al.* data set similar to this study, incorporated both 2D and 3D descriptors, and was also included in the comparison of QSAR models (Table S5).

**Figure 4:**
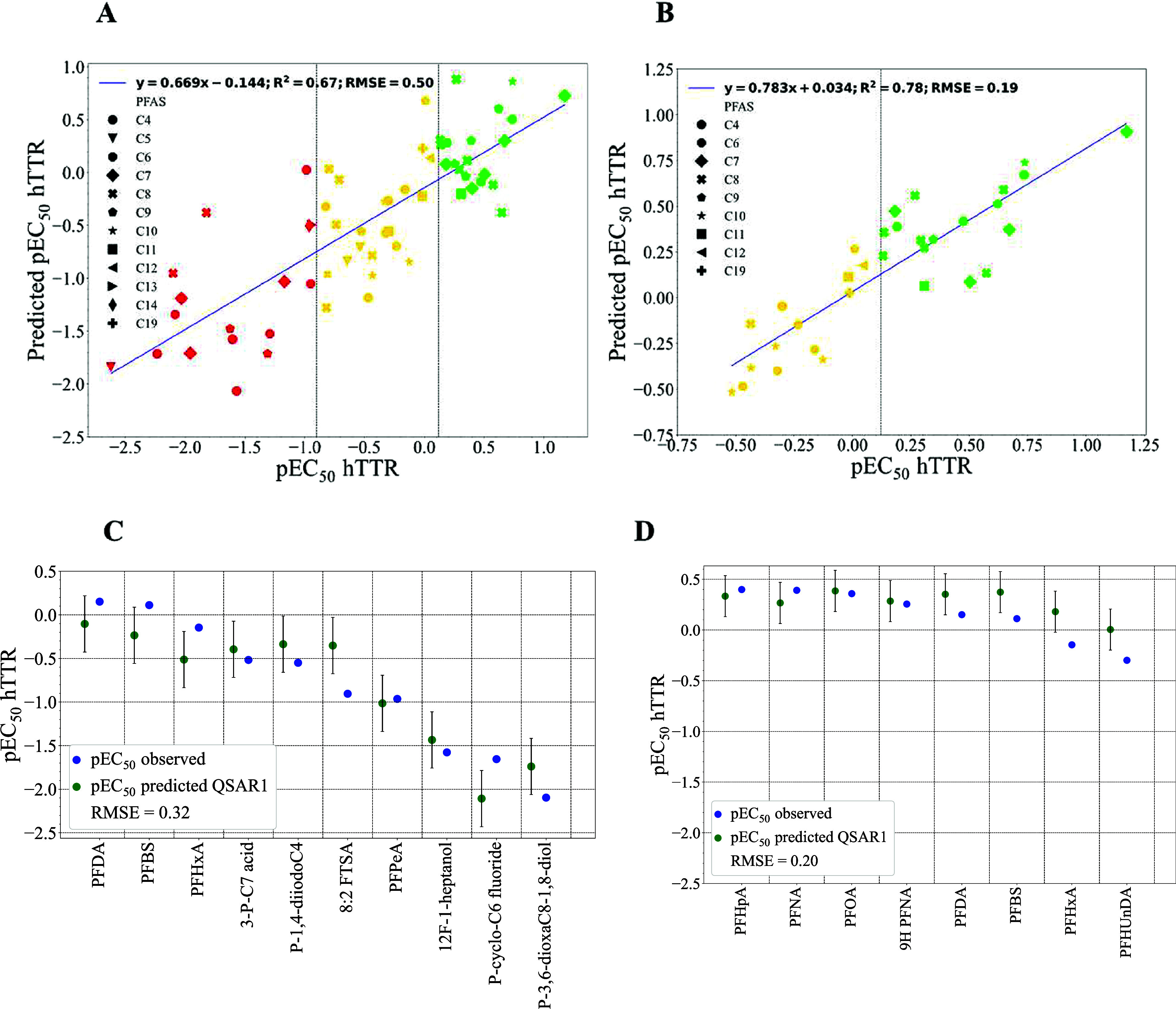
PFAS-hTTR QSAR models: (A) QSAR Model 1 derivation (training set); (B) QSAR Model 2 derivation (training set); (C) QSAR Model 1 validation (test set); and (D) QSAR Model 2 validation (test set). (A, B) Green, yellow, and red correspond to strong, medium, and weak binders, respectively. Error bars (C, D) represent RMSE values for the test set.

**Figure 5:**
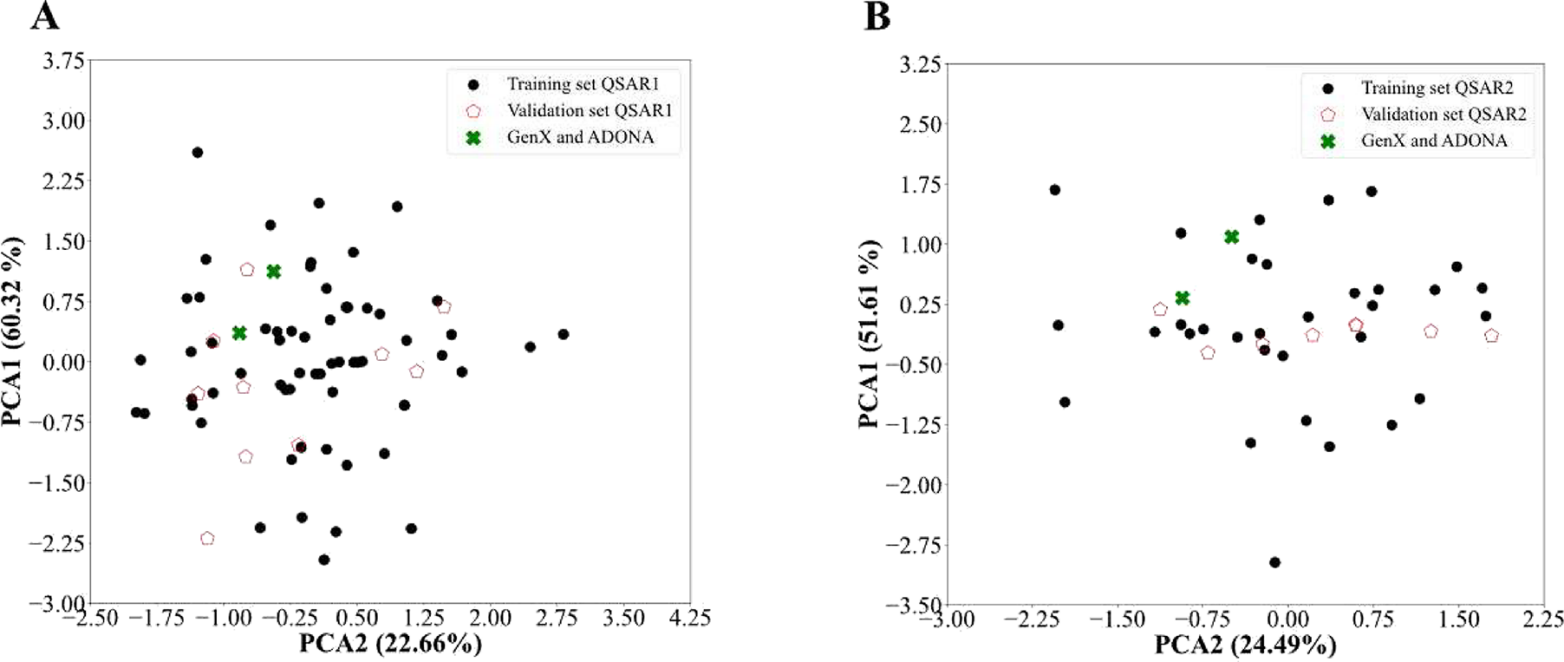
Principal component (PCA) analysis for QSAR Model 1 (A) and QSAR Model 2 (B) with PCA1 and PCA2 variance.

**Figure 6:**
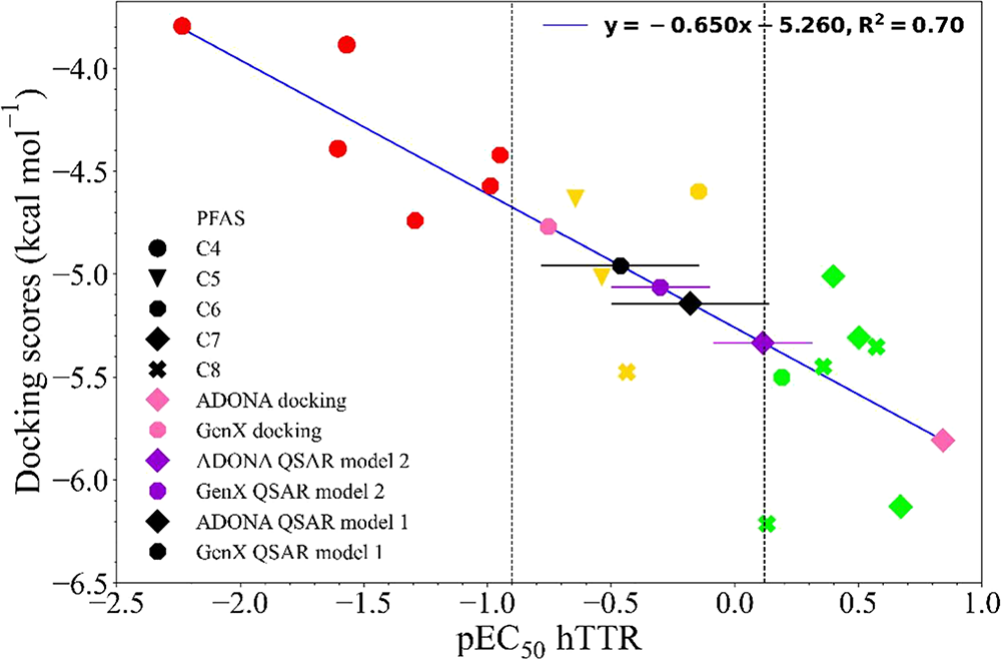
Correlation plot between docking scores and experimental pEC_50_ values for PFAS-containing carboxylic groups (4–8 carbons). Green, yellow, and red correspond to strong, medium, and weak binders, respectively. ADONA and GenX predictions are provided with docking (pink) and two different QSAR models (QSAR Model 1, black; QSAR Model 2, purple). Error bars for ADONA and GenX values (*x*-axis) represent the RMSE values from the test set of the corresponding QSAR model.

The two QSAR models reported here were the best fit and possess unique features. For the first QSAR model, the *R* ^2^ and RMSE are 0.67 and 0.50, respectively (Figure 4A). The validation set for this model in Figure 4C shows that it worked equally well for strong, medium, and weak binders (the test set RMSE is 0.32). The comparatively better performing QSAR Model 2, which was developed for strong and medium binders, had a smaller RMSE (0.19) and an *R* ^2^ of 0.78 (Figure 4B). As expected, this model provided good predictions for strong and medium binders (Figure 4D, the test set RMSE is 0.20). The cross-validation plots (leave-one-out cross-validation, LOO CV) for QSAR Models 1 and 2 are included in the SI (Figures S2 and S3). Based on the model characteristics, QSAR Model 1 offers broad applicability across diverse PFAS structures, while QSAR Model 2 can produce accurate predictions for strong and medium binders, with a smaller RMSE. The QSAR models were also used to predict EC_50_ values for T_4_/TTR displacement for ADONA and GenX. QSAR Model 1 predicted ADONA and GenX as medium binders with pEC_50_ values of −0.180 and −0.462, respectively. Similarly, QSAR Model 2 predicts ADONA and GenX as medium binders with pEC_50_ values of 0.114 and −0.301, respectively. In both models, ADONA is a stronger binder than GenX (Figure 6). The top descriptors for each model are presented in Table S6 in the Supporting Information.

DoA was defined for the QSAR models by principal component analysis (PCA) (Figure 5A and 5B). PCA used the two most relevant descriptors, which were a_count and b_single in both QSAR models, although with switched order (Table S6). DoA for QSAR Model 1 is more extensive than the DoA for QSAR Model 2 (greater range on either PCA axis), reflecting the inclusion of lower-affinity PFAS that are more structurally diverse. Smaller DoA for Model 2, which is optimized for stronger binders, likely indicates more specific structural parameters that favor binding. For both models, validation sets and tested chemicals GenX and ADONA are located well within the DoA (Figure 5A,B).

### Comparison with Previous QSAR Studies

The mechanism of TTR/T_4_ displacement by PFAS has been investigated in several previously published QSAR models, which include classification and regression models (as summarized in Table S5). Weiss *et al.* ^43^ reported a TTR/PFAS experimental data set but concluded that it was not sufficient for an effective regression QSAR (*R* ^2^ 0.61). In contrast, Kovarich *et al.*,^56^ Papa *et al.*,^57^ and Kar *et al.* ^58^ leveraged the same Weiss *et al.* data set for better performing QSAR models (*R* ^2^ values for regression models 0.87–0.89). One notable difference between these studies and Weiss *et al.* was the inclusion of two-dimensional and three-dimensional descriptors.

In 2024, the Tox24 challenge took place, focusing on the prediction of *in vitro* binding of multiple chemicals to TTR.^73,74^ Seventy-eight teams used Tox21 ^75,76^ and Degitz *et al.* ^45^ data sets to create QSAR models for TTR binding, which were validated by blind testing with a separate set of 300 chemicals. The top two competitors chose machine learning algorithms for their best models/predictions: the winning team (Makarov *et al.* ^77^) used Transformer Convolutional Neural Networks (CNN),^78^ and the second-placed team (Cirino *et al.* ^79^) used Convolutional Neural Fingerprints (CNF2),^80^ CNN,^78^ and Graph Neural Networks (GNN).^81^ Makarov *et al.* chose an ensemble of four methodologies for their best-performing model and included descriptors based on chemical mixtures, while Cirino *et al.* optimized their model by using tautomers to help validate active compounds.^74,77,79,82^ Both models used the Online CHEmical database and Modeling environment (OCHEM) to submit their models.^83^ Only the Makarov *et al.* model is included in Table S5, as the representative study of the Tox24 challenge.

Similar to the Tox24 challenge, Evangelista *et al.* expanded the training set to more than 200 heterogeneous chemicals that included 14 PFAS (from Weiss *et al.*).^43,59^ Additional alternative approaches have been used. Zhao *et al.* constructed a TTR QSAR model based on developed docking scores (we experimented with a similarly constructed model in this study, see discussion below).^55^ Sosnowska *et al.* utilized activity values from an *in vitro* reporter bioassay that would indirectly measure ligand binding to TTR as a basis for several QSAR models.^49^ This study also investigated the combination of multiple regression models into a model suite to optimize prediction.

Despite differences in data sets and modeling approaches, there is some level of similarity in descriptors across QSAR models (Table S5) and models developed here. Some of the common top descriptors, e.g., in Weiss *et al.*,^43^ Makarov *et al.*,^77^ Zhang *et al.*,^52^ and Zhao *et al.*,^55^ overlap with our choices, for example, the topological surface area, molecular size, and van der Waals interactions, potentially indicating important factors for effective binding. Surprisingly, despite using similar or significantly overlapping training sets and molecular descriptors derived from the same limited platforms (DRAGON, MOE, or PaDEL), there is no overlap in top descriptors among models. Additional discussion of the important issue of the relevance of top QSAR descriptors to the TTR binding mechanism is included below.

One recurring challenge in developing effective QSAR models for TTR-PFAS binding is the presence of apparent outliers that could be due to the inherent nonuniformity of structurally diverse PFAS data sets. For instance, Weiss *et al.* excluded PFBS from the test set to improve the accuracy of the developed model. Similarly, Evangelista *et al.* excluded multiple PFAS outliers from the two PFAS-containing QSAR models.^59^ Exclusion of outliers to improve model performance, however, may not be an optimal strategy, as it would decrease the overall applicability domain of the model. In the present study, the same effect was observed, exacerbated by many structurally diverse PFAS in the data set. Rather than developing a model for the best-performing PFAS subset (and excluding the worst-performing PFAS as outliers), we chose a tiered model strategy: a more encompassing, if less efficient, model that can separate PFAS into low, medium, and high binding categories and the second highly efficient model that predicts binding affinity for medium and high binders. Evangelista *et al.* also ranked TTR disruptors as strong, moderate, and weak binders; however, that classification was based on relative potencies.^59^ Both QSAR models predicted medium–high TTR binding potentials for ADONA and GenX.

### TTR Docking Models

PFAS-TTR docking models were developed using human structures and rat TTR cocrystallized with T_4_.^64,65^ Each structure has four T_4_ subunits. hTTR has two equivalent binding sites, while rTTR has two nonidentical binding sites. Figure 3 demonstrates binding site 1 for human and rat TTRs (Figure 3A,C, respectively), and Figure S1 shows a complete description of both pockets. The docking scores for hTTR-PFAS binding are provided in Table S7. A key stabilizing residue common to each structure is Lys15, which is consistent with a previous mutagenesis study, which found this residue to be essential for T_4_ and PFAS binding in hTTR.^44^ However, for rTTR (Figure 3B), additional binding differences were observed: besides interacting with Lys15, T_4_ formed a hydrogen bond with Glu54 via its NH_3_^+^ group. This interaction results in the twist of the T_4_ NH_3_^+^ group in the pocket compared to the human crystal structure, as detailed in Figure S1. The geometries of the TTR/T_4_ complexes and mutagenesis data for hTTR/T_4_ binding (Lys15) were used to generate human and rat pharmacophore models for TTR ligand binding sites (Figure 3B,D). For hTTR, the pharmacophore model was used to generate docking poses for seventy-two PFAS, for which binding data are available from the experiment.

When the docking scores were compared to experimental binding data, the correlation was poor (Figures S4 and S5). The correlation was slightly improved when only the docking scores below 6 kcal mol^–1^ were included (Figure S5). Specifically, PFSAs were generally overestimated (along with additional smaller PFAS) with predicted lower binding scores (more negative) despite having low binding affinities, resulting in placement into an incorrect binding range. However, PFCAs containing four to eight carbons (17 compounds) showed a strong correlation between docking scores and pEC_50_ values (*R* ^2^ = 0.70, Figure 6). This specificity suggests that the hTTR docking model is more reliable for PFCAs (*N* = 4–8), highlighting the importance of ligand similarity for accurate predictions. In this docking model, ADONA and GenX resulted in docking scores of −5.808 and −4.770 kcal mol^–1^, respectively. In the resulting linear correlation, these docking scores correspond to pEC_50_ estimates of 0.94 and −0.66, respectively (Figure 6). Interestingly, these docking score-based estimates would place GenX in the medium binding range (−0.9 ≤ pEC_50_ ≤ 0.12), while ADONA would be classified as a strong binder (0.12 < pEC_50_). In comparison, both QSAR models described above (QSAR1 and QSAR2) place ADONA and GenX in the medium binding range (black and purple data points in Figure 6, area between two dashed vertical lines indicates medium binding range), although the QSAR2 estimate for ADONA (0.114) is just below the strong binding range (pEC_50_ > 0.12). Overall, all three correlation models (based on docking scores, QSAR1 and QSAR2) demonstrated comparable predictions for ADONA and GenX, identifying both compounds as at least medium binders.

While previous studies have developed PFAS-hTTR docking models, they have not been formally (externally) validated.^44,51,54,55,58^ In contrast, the hTTR docking model generated here was based on mutagenesis evidence, and moreover, it was successfully validated with two independently sourced human TTR-PFOA and TTR-PFOS crystal structures, which demonstrated nearly perfect overlap with the modeled hTTR-PFOA^50^ and hTTR-PFOS^51^ structures, providing strong support for the model’s accuracy.

### Comparison of QSAR and Docking Approaches

In ligand–receptor experimental systems with available binding data (such as PFAS-hTTR), QSAR and docking models serve complementary roles. QSAR models use structural and physicochemical descriptors to enable formal predictive analysis, including the domain of applicability considerations. Docking models maximize the use of structural data (for both the receptor interface and the ligand) and serve as the foundation for molecular dynamics simulations by generating initial ligand binding poses. In contrast, the domain of applicability of the docking model cannot be easily defined. To expand the structural applicability of the docking, molecular dynamics can be used to help refine binding poses and allow for a more accurate calculation of binding energies. Moreover, the easy-to-interpret mechanistic insights derived from the docking and molecular dynamics models provide an interesting contrast to the important issue of the mechanistic interpretation of QSAR models, as described in Table 1 and detailed below.

**Table 1:** Comparison of T_4_/PFAS Binding Site Properties (as Supported by Docking/MD Conclusions) with Applicable Top Descriptors in QSAR Models (Mechanistic Relevance of QSAR Models)

T_4_/PFAS binding site properties	Supporting evidence from docking/molecular dynamics model	**TTR-PFAS QSAR1 TTR-PFAS QSAR2** (this study)	Makarov *et al.* ^a^ catBoost/AlogPS/OEstate model
Primary stabilization through ionic bonds to Lys 15 “charge advantage”	Success of the pharmacophore modeling	Number of oxygens (a_nO, #5)	Polar surface area (PSA #4)
	Binding stabilization with Lys 15 (Figure 8B)		Carboxyl group (Se1C3O1a, #2)
Optimum molecule length for linear PFAS mimics T_4_ molecular length^b^ (16 Å) “length limitation”	Highest affinity is predicted for PFNA (15.6 Å—molecular length^b^), lower affinity for short and long chained	Number of atoms (a_nc, a_count, #1, #3) molecule linearity (balabanJ, #8)	Molecular weight (MW, #8)
Spatially shifted PFAS binding site due to hydrophobic properties “hydrophobicity disadvantage”	PFAS bind distinctly than T_4_ and away from Ser 117 and Glu 54; minor/no stabilization from hydrophobic interactions	Lipophilicity (GCUT_SLOGP_3, #5) van der Waals interactions (E_vdw, #4 in QSAR2 only)	Lipophilicity (ALogPS_logP #1, ALogPS_logS #3)

a Winner of the Tox24 challenge.

b Maximum distance between any pair of atoms within the molecule.

The most recent update of the OECD QSAR assessment framework^84^ lists plausibility of mechanistic interpretation of a QSAR model as one of the five essential principles to facilitate the consideration of a (Q)­SAR model for regulatory purposes. For the equation-based models (such as those developed in this study), the mechanistic interpretation can be based on physicochemical interpretation of each descriptor and its association with the mechanism of action.^84^ Table 1 lists and explains three important mechanistic considerations of the PFAS/TTR binding mechanism (as we named them): “charge advantage,” “length limitation,” and “hydrophobicity disadvantage.” These considerations are supported by external evidence (mutational analysis for Lys 15) and by the human docking and molecular dynamics models. As demonstrated in Table 1, the interpretation that can be inferred between these mechanistic considerations and the top descriptors in the QSAR models developed in this study and the representative Makarov *et al.* PFAS/TTR QSAR model (winner of the Tox24 challenge) is somewhat limited. For example, the presence of oxygen (QSAR models in this study) or carboxyl groups (Makarov model) would only weakly point to the observed “charge advantage” (Table 1). From a regulatory perspective, the interpretability of the QSAR models included in Table 1 is more meaningful in the context of the corresponding docking and MD models.

QSAR models based on docking scores have been previously reported.^55,85–87^ The limitations of this approach were demonstrated by a similar model experiment conducted in this study. The QSAR model for hTTR-PFAS was developed based on docking scores but only successfully converged when the training set was limited to carboxylates (Figure 6). A similar attempted model with all PFAS resulted in a low R^2^ (0.12) when predicted values were compared to observed values (Figure S4). We interpreted this result as an inability of this carboxylate (i.e., T_4_)-based docking model to effectively capture distinct binding characteristics of noncarboxylate PFAS molecules compared to carboxylates. Thus, future improvements to QSAR models may include docking scores (as part of descriptor sets) but also explore multiple binding modes in developing chemically specific binding score predictions.

ADONA and GenX, two PFAS that are not included in the Degitz *et al.* TTR *in vitro* binding data set, have been predicted to bind TTR in all in silico models developed in this study (Figure 6). Interestingly, weak binding of GenX was reported in the study by Langberg *et al.* ^47^ Moreover, pregnant female rats exposed to GenX during gestation demonstrated reduced serum total T_4_ levels,^88^ which is consistent with the proposed PFAS-mediated TTR/T_4_ displacement mechanism. No *in vivo* data for thyroid hormone effects are available for ADONA.

### Comparison of Human and Rat TTR-PFAS Binding Using Molecular Dynamics

Molecular dynamics simulations were performed for both human and rat TTR models, enabling direct structural interactions and calculating the free binding energies for the two species. The last frame for each of the PFOA and PFOS simulations in the human model was aligned with its respective crystal structure (Figure 7), showing nearly perfect overlap and similar residue interactions, thereby validating the approach and the resulting docking models.

**Figure 7:**
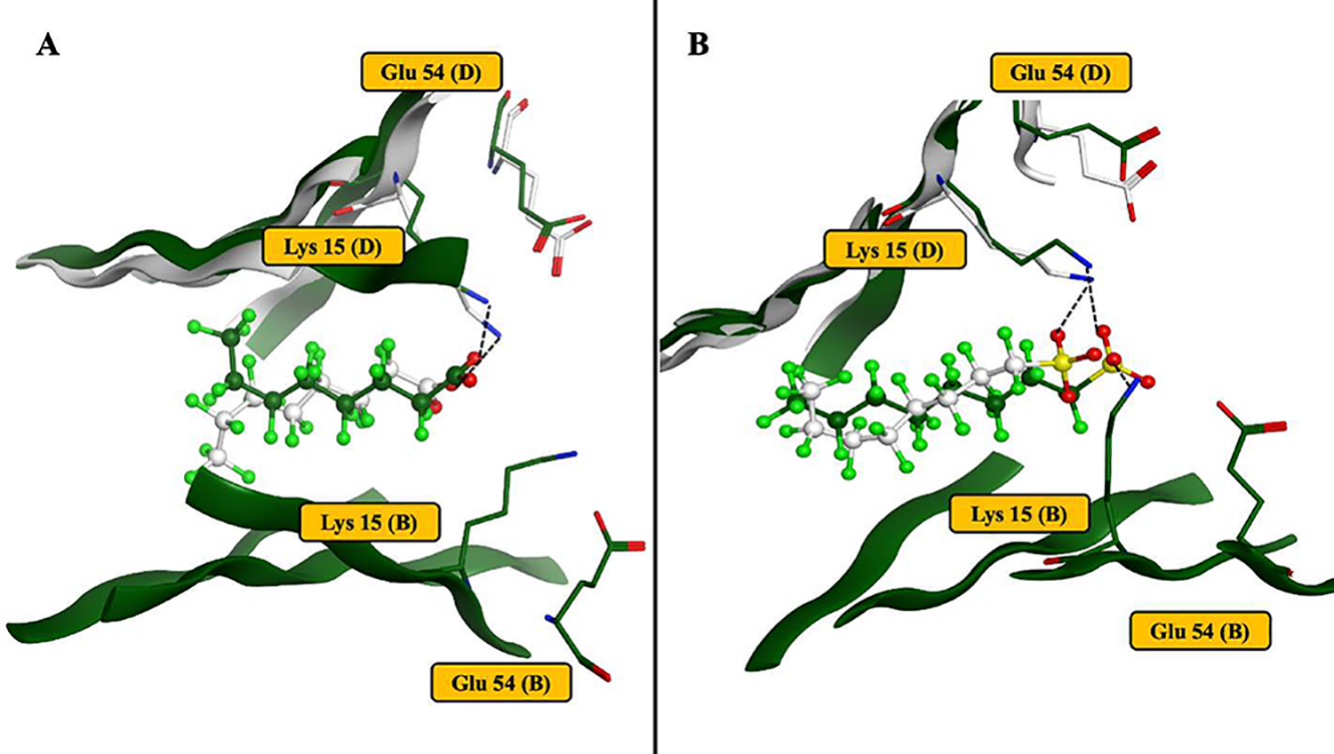
Representative modeled PFOA (A) and PFOS (B) binding poses (dark green) overlapped with TTR/PFOA and TTR/PFOS crystal structures (gray).^50,51^ Modeled binding poses demonstrate excellent agreement with published structures.

Human and rat models were simulated for 100 ns, and binding energies for each analyzed PFAS were estimated using MM-PBSA and compared between species (Figure 8A). The MD simulations showed consistent and stable results, with converging RMSDs, both for the protein and the ligand (Figures S6–S33). Protein RMSDs stabilized/plateaued around 2 Å within the first 5 ns for all simulated PFAS and T_4_. The averaged binding energy for T_4_ was −40.01 ± 4.34 kcal mol^–1^ in the human model, −35.97 ± 4.02 kcal mol^–1^ for the rat binding pocket 1, and −36.14 ± 3.97 kcal mol^–1^ for the rat binding pocket 2 (Table S8). In the human model, short-chain PFAS (PFBA, PFBS) were relatively less stable in the pocket than longer-chain PFAS. For example, during the hTTR MD simulation, PFBA repeatedly broke the hydrogen bond with Lys15, resulting in a higher RMSD (Figure S9). PFBS was also comparatively less stable, showing some movement within the pocket (Figure S17). For rat, PFBA and PFBS were comparatively more stable within the pocket (Figures S24 and S30, respectively). Despite fewer stable poses with hTTR compared to rTTR, short-chain PFAS (PFBS, PFBA) demonstrated binding energies comparable to those of these two proteins (Figure 8A). The long-chain PFAS (PFHxA, PFHxS, PFHpA, PFHpS, PFOA, PFOS, PFNA, PFDA, ADONA, and GenX) maintained stable poses within the pocket for both human and rat TTR models (Figures S10–S16, S18–S20, S25–S29, and S31–S33), consistent with their stronger binding affinities.

**Figure 8:**
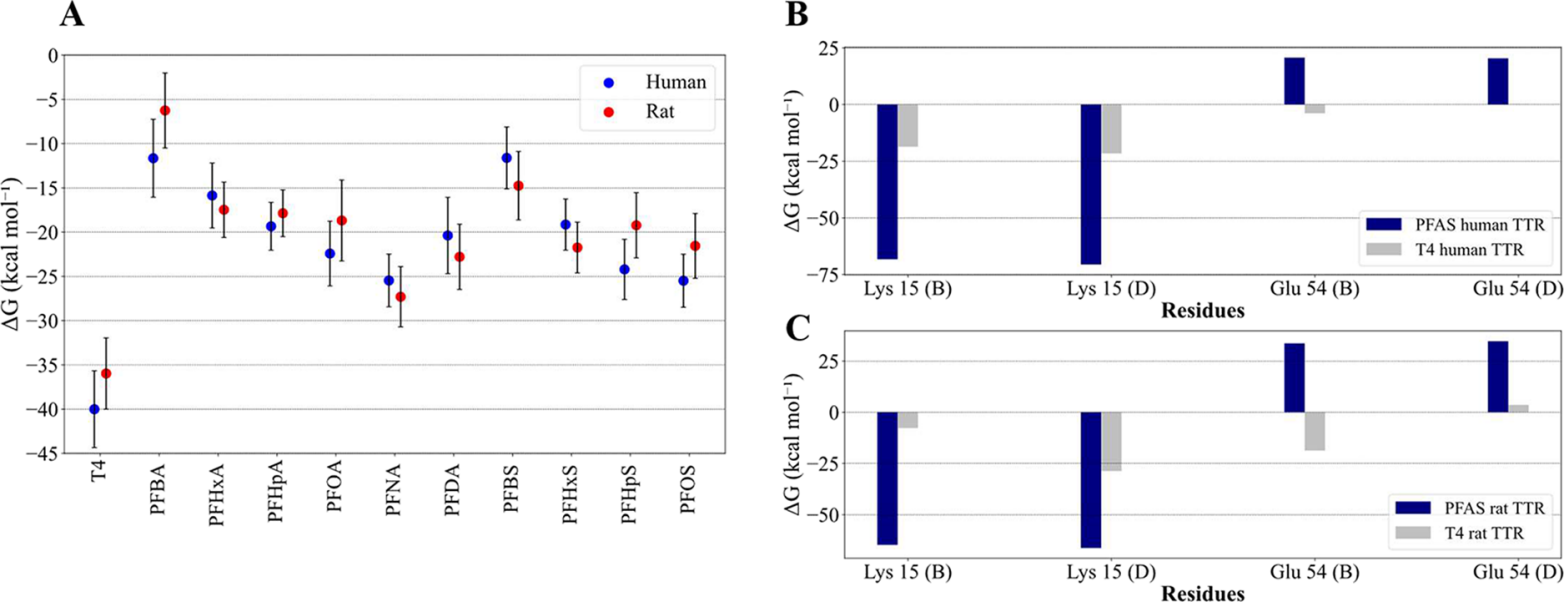
PFAS binding to hTTR and rTTR demonstrates similar binding energies and comparable contributions from individual amino acids. (A) Free binding energies calculated for human and rat models with MM-PBSA. Values are averages of three independent runs and bars are standard deviations. (B, C) Comparison of residue decompositions for hTTR (B) and rTTR (C) between all PFAS bound averaged MD simulations and T_4_ bound MD simulations. Letters B and D in parentheses after each amino acid refer to the two TTR subunits in the dimer.

A direct comparison of free binding energies across species (Figure 8A) showed striking similarity between the human and rat models, despite differences in their initial docking poses (Figure 3A,C). This demonstrates cross-species similarity of TTR-PFAS binding, which is important for species extrapolation of toxicological outcomes. To further investigate this similarity, residue decomposition analysis was conducted for PFAS and T_4_ simulations (Figure 8B). Interestingly, in either hTTR or rTTR, T_4_ binds very differently compared to PFAS. However, the PFAS interactions with individual residues were quite similar between the human and rat models. A notable species difference was observed for T_4_, which forms a strong hydrogen bond with Glu54 (subunit B) in rTTR but not in hTTR. For PFAS, the deprotonated acid group of Glu54 (subunit B) appears to repel PFAS more strongly for rTTR than for hTTR, with the resulting higher positive contribution for rTTR (Figure 8B). This could be due to this residue being located closer to the bound ligand in rTTR.

### Comparison of Predicted Binding Energies to *In Vivo* Toxicity

The difference between the calculated free binding energies (Figure 8A) of PFAS and that of T_4_ represents the free energy change of the PFAS-T_4_ displacement and can be correlated with the *in vivo* potency data for PFAS-induced reductions in serum T_4_ levels in the male rat as evidence in support of the proposed AOP.^42^ This analysis would inform the applicability of the developed models to predict toxicity *in vivo*, specifically in the AOP context.

Better *in vivo* comparisons among PFAS are based on internal exposures (such as averaged serum concentration) to account for dramatic toxicokinetic differences among species and PFAS.^41^ Thus, toxicokinetic adjustments were applied to studied PFAS (PFBA, PFBS, PFHxA, PFHxS, PFOA, PFOS, PFNA, and PFDA), as described in the Supporting Information, and serum concentrations were expressed with two metrics: *C*_max_, indicating maximum achieved plasma concentration during treatment, and TWA, time-weighted average serum concentration during treatment. Use of different internal metrics can further inform toxicity comparisons.

Available toxicity data for PFAS were reviewed.^2,9,10,17,24,25,27–36^ Most studies that analyzed thyroid effects reported some changes in the thyroid hormone levels. Total and free plasma T_4_ levels were selected as primary end points, as these effects have been reported for most PFAS of interest in this study, and they are linked to the proposed PFAS-T_4_ direct displacement mechanism. Male rats were chosen for this analysis since in female rats, thyroglobulin (and not TTR) has been previously found to be primarily responsible for serum T_4_ stabilization.^89^ Toxicity analysis included PFBA, PFBS, PFHxA, PFHxS, PFOA, PFOS, PFNA, and PFDA. Applied doses were converted into effective serum concentrations (*C*_max_ and TWA), and the resulting dose–response data were analyzed by BMD analysis, as described in the Methodology section. The resulting benchmark dose values (BMDs) are considered to be appropriate metrics for potency comparisons among compounds. The correlation of BMDs with PFAS/TTR binding energies was analyzed with Bayesian linear regression. Variances (standard deviations) for the two arguments were included in this analysis. Variances for BMDs were derived from the 95th percentile upper and lower (BMDL, BMDU) from the ToxicR’s MCMC model average outputs. Variances for the binding energies were adopted from the selected MD simulations. Fit and validation metrics (including root-mean-squared error, RMSE; Bayesian R-squared; and leave-one-out cross-validation, LOO CV) were calculated.

Predicted binding energy of PFAS to rTTR (averaged over the first 90 ns of MD simulation) correlated well with both *C*_max_ and TWA BMDs for the studied PFAS (Figure 9A,B respectively). The strongest correlation was for *C*_max_ and free T_4_ levels (Figure 9A), which had a plausible biological slope (3.66; 95% CI: −2.47, 10.48) with an intercept of 103 (95% CI: −5.95, 236), an *R* ^2^ of 0.325, and an RMSE of 53 μM. The performance with TWA was nearly equivalent, with a slope of 2.46 (95% CI: −1.86, 7.43) and an intercept of 67.8 (95% CI: −8.44, 168), an *R* ^2^ value of 0.282, and an RMSE of 39 μM (Figure 9B). The important observation in these experiments is the statistically plausible positive slope under conservative uncertainty assumptions, although the credibility interval for the slope overlaps with zero. The simplified analysis of linear trend only using mean values is presented in Figure S34. In this analysis, *R* ^2^ for the linear trend was 0.65 for *C*_max_ vs binding energy and 0.63 for TWA vs binding energy. In either approach (Figures 9 and S34), there were no clear differences in the goodness of fit between using *C*_max_ or TWA as metrics; thus, no conclusion can be made regarding the most appropriate metric.

**Figure 9:**
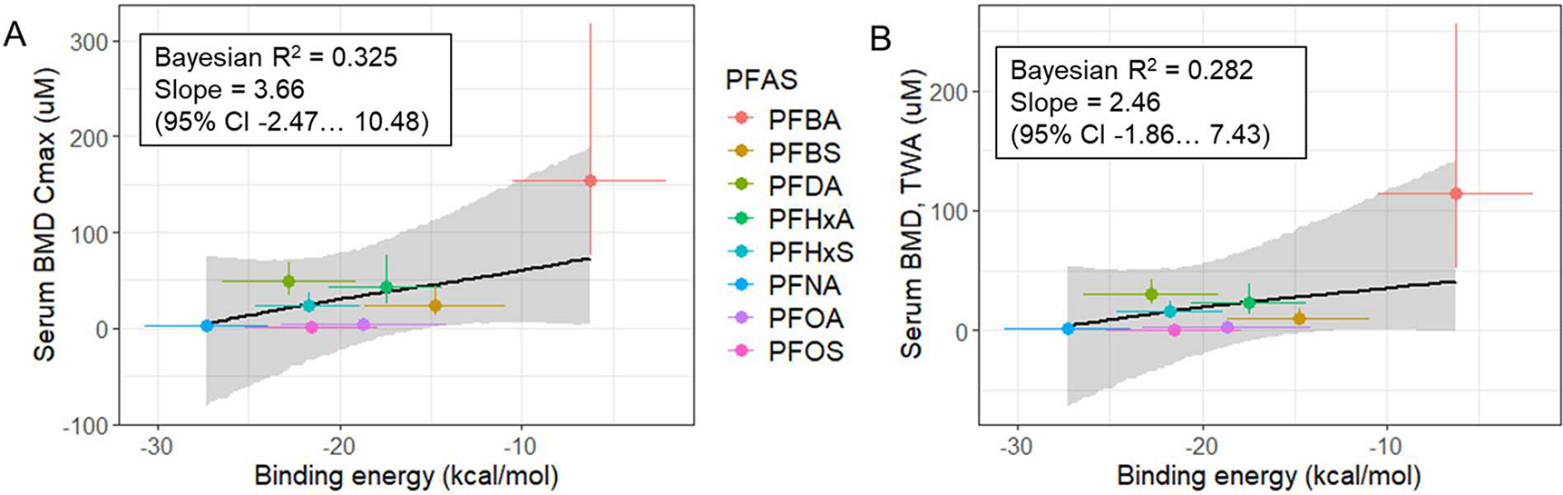
PFAS/rTTR free binding energies correlate with *in vivo* points of departure (PODs) for serum free T_4_ decreases in male rats expressed as serum benchmark doses (BMD). Free binding energies were calculated with molecular dynamics simulations. For toxicity studies, applied doses were converted to (A) maximum plasma concentrations (*C*_max_) and (B) time-weighted averaged (TWA) plasma concentrations using PFAS-specific toxicokinetic models, BMDs and standard geometric errors of posterior distributions were calculated in ToxicR; Bayesian regression was fitted against modeled binding energies. Shaded area represents a 95% credibility interval for linear regression. Data points are modeled distribution means, and error bars represent geometric standard errors (*y* axis) and standard deviations (*x* axis).

Correlations developed for total T_4_ with both metrics (*C*_max_ and TWA) were generally similar to the correlations for free T_4_ (data not shown). These findings support the PFAS-T_4_ displacement mechanism as a rate-controlling step in the proposed AOP. Two additional considerations arise. First, the mechanism appears to operate within PFAS as a group since data points from multiple PFAS contribute to the observed correlation. Second, the displacement step could be rate-limiting (i.e., proportionally affecting the apical effects—observable outcomes) and therefore may represent the molecular initiating event (MIE) of this AOP. This conclusion is preliminary due to the limited number of data points available for analysis and additional sources of uncertainty related to the use of simplified toxicokinetic models.

Importantly, alternative mechanisms of thyroid toxicity have also been proposed for PFAS, including inhibition of the Na^+^/I^–^ symporter and binding to the thyroid receptor.^90,91^ Thus, the discussion of the underlying mechanisms of thyroid toxicity of PFAS is ongoing. While the AOP observations and evidence proposed here are for an animal model (male rat), they can also inform thyroid toxicity considerations of PFAS in humans, particularly since several available health evaluations for some PFAS (PFBS, PFHxA, PFHxS) from U.S. EPA and California’s Office of Environmental Health Hazard Assessment (OEHHA) are based on animal thyroid toxicity studies as the most sensitive end point.^2,9,10,17,24,25,27–36^

PFAS effects on thyroid hormone levels (T_3_, T_4_, TSH) have been extensively investigated in epidemiological studies.^2,9,10,28–33,35^ Some meta-analyses found mixed effects on T_3_ and T_4_ in the general population although one recent study found an overall TSH increase in pregnant females.^92,93^ Most animal studies are conducted at higher PFAS doses compared to observed human exposures, which could be one reason for the difference from human data. Thus, *in vitro* binding data for PFOA and PFOS (as an example) indicate effective displacement at approximately 0.5 μM, which is about an order of magnitude higher than the higher end of reported plasma concentrations (approximately 10 ng mL^–1^ or 0.025 μM) in most epidemiological studies.^34^ Interestingly, in one occupational study, Olsen *et al.* reported upper limit PFOA exposure at higher levels (maximum, 92 ng mL^–1^) and found PFOA-dependent decreases in free T_4_ when adjusted for PFOS.^94^ Thus, PFAS serum levels in general population studies appear to be generally below the TTR/PFAS displacement levels, although most analyses have not considered mixture effects, human variability, or sensitive subpopulations.

## Final Remarks

TTR models developed in this study are summarized in Table 2. Three fundamental aims were addressed: (1) evaluating species differences between human and rat TTR-PFAS interactions (using docking and MD models); (2) predicting binding potential using QSAR models; and (3) assessing how in silico predictions of rTTR-PFAS binding energies correlate with *in vivo* points of departure for PFAS-dependent T_4_ decreases. This study demonstrated how docking/MD models and QSAR models can be complementary in analyzing and predicting the structural interaction of a ligand with its protein target.

**Table 2:** TTR Model Comparisons

TTR model	Rationale	Main results/Predictions	Interpretation/Limitations
Human QSAR models	Maximize use of *in vitro* hTTR binding data	Predictive model with defined DoA	In tandem, models can be used to predict PFAS binding to hTTR
		Prediction: ADONA, GenX	
Human docking	Based on crystal structure	Basis for human MD model	Predicted hTTR-PFOA and hTTR-PFOS structures matched well with independently reported crystal structures
	Use mutation data (Lys15) to design the TTR-PFAS pharmacophore and to improve docking approach	Prediction: ADONA, GenX	As prediction model: only 4–8 carbons PFCAs
Rat docking	Based on crystal structure	Basis for rat MD model	Limitation: no *in vitro* rTTR-PFAS data for validation
Human MD	Investigate interspecies (rat-human) differences in PFAS binding to TTR	Binding modes for human and rat TTR-PFAS appear similar	Limitation: time- and resource-consuming approach
Rat MD			
Comparison with free T_4_↓ (*in vivo*)	Investigate TTR-PFAS displacement as a possible mechanism for free T_4_↓ in male rats	Moderate correlation was observed between in silico calculated rTTR-PFAS free binding energies and *in vivo* PODs for free plasma T_4_ decrease in male rats	Developed in silico evidence is consistent with the AOP; limitation: empirically observed correlation

Development of predictive models for PFAS toxicity is of paramount importance given the spread in the environment and accumulation in humans for these chemicals, continually increasing variety of chemical structures, and the lack of actionable toxicity studies for most chemicals in the group. To date, the approach in the literature has been somewhat piecemeal, with separate types of models (e.g., QSAR, docking, mechanistic) explored in distinct studies and for a limited number of PFAS. The relationship between published models of different types has not been clear, and important aspects, including toxicokinetic adjustments, species extrapolations, and uncertainty considerations, have not been fully addressed. Here, we develop and bring together different types of structural and toxicity models (summarized in Table 2) with the overall intention to maximally utilize available experimental data for model development and validation, and to establish clear roles for different models in distinctly informing the overall framework. Our approach has been pragmatic: we acknowledged important challenges (such as toxicokinetic adjustments, interspecies extrapolation and data gaps, and uncertainty considerations) and attempted to characterize them quantitatively. The proposed approaches were also evaluated with novel PFAS (GenX, ADONA), demonstrating good agreement between the predictions and the limited available data. The resulting framework can and will be further improved as additional *in vitro* and *in vivo* data become available and with better understanding of the overall toxicity of PFAS.

## Supplementary Material





## Data Availability

All human and rat TTR-PFAS poses, and molecular dynamics calculation input files are made available in Zenodo (DOI: 10.5281/zenodo.17059159).

## Notes

Table S4: Toxicokinetic models and parameters used to estimate serum concentrations; Table S5: QSAR comparison: models that predict TTR/PFAS binding potencies; Table S6: top descriptors for QSAR models 1 and 2 (listed in other order of descending importance); Table S8: averaged binding energies for human and rat MD models; Table S9: measured serum concentration data for PFAS in male rats used to test performance of toxicokinetic models; Table S10: model performance metrics for best-performing toxicokinetic models for each PFAS chemical for male rats; Figure S1: crystal structures for human and rat TTR; Figure S2: QSAR Model 1 cross-validation data; Figure S3: QSAR Model 2 cross-validation data; Figure S4: all docking scores versus pEC_50_; Figure S5: docking scores (below 6 kcal mol^–1^) versus pEC_50_; Figures S6–S33: human and rat RMSD plots (100 ns); Figure S34: PFAS/rTTR free binding energies correlate with *in vivo* points of departure (PODs) for serum free T_4_ decreases in male rats; and Figure S35: toxicokinetic modeled vs measured serum concentrations of PFAS in male rats (PDF)
Table S1: All PFAS with available *in vitro* data from the Degitz *et al.* data set; Table S2: PFAS molecules used in QSAR Model 1, along with experimental pEC_50_ values from Degitz *et al.*, predicted, and cross validated pEC_50_ values; Table S3: PFAS molecules used in QSAR Model 2, along with experimental pEC_50_ values from Degitz *et al.*, predicted, and cross validated pEC_50_ values; and Table S7: PFAS used for docking models and corresponding pEC_50_ values and docking scores (ZIP)
