# Supplementary material for: New Docking, Molecular Dynamics, and QSAR Models to Predict Disruption of Human and Rat Transthyretin Function by Per- and Polyfluoroalkyl Substances (PFAS)
Source: Chem Res Toxicol. 2026 Apr 27;39(5):835–51. doi: 10.1021/acs.chemrestox.5c00424 (PMC13188166; doi:10.1021/acs.chemrestox.5c00424)
Supplement: Supplementary file 1 [file tx5c00424_si_001.pdf]

Supporting Information

New Docking, Molecular Dynamics and QSAR Models to Predict Disruption of Human and Rat Transthyretin Function by Per- and Polyfluoroalkyl Substances (PFAS)

Nuno M. S. Almeida[a,b*], Heather M. Bolstad[a], Scott Coffin[a], Sana Majid[a], Angela K. Wilson[b], Anatoly A. Soshilov[a*]

[a]New Toxicology Evaluations Section (NTES), Office of Environmental Health Hazard Assessment, California Environmental Protection Agency
1001 I St, Sacramento, CA, 95814, USA
[b]Department of Chemistry, Michigan State University, East Lansing, MI, 48864, USA

* Corresponding authors (nuno.almeida@oehha.ca.gov; anatoly.soshilov@oehha.ca.gov)

Table of Contents

Table S4 – Toxicokinetic models and parameters used to estimate serum concentrations S2
Table S5 – QSAR comparison: Models that predict TTR/PFAS binding potencies S3
Table S6 – Top descriptors for QSAR models 1 and 2 (listed in the order of decreasing importance) S7
Table S8 – Averaged binding energies for human and rat MD models S7
Table S9 – Measured serum concentration data for PFAS in male rats used to test performance of toxicokinetic models S7
Table S10 – Model performance metrics for best-performing toxicokinetic models for each PFAS chemical for male rats S8
METHODOLOGY S8
hTTR and rTTR docking models S9
Molecular dynamic simulations S9
Toxicokinetic modelling S9
TK model evaluation S11
SUPPORTING FIGURES S11
Figure S1 – Crystal structures for human and rat TTR. S12
Figure S2 – QSAR model 1 cross validation data – LOO (from Figure 4A). S13
Figure S3 – QSAR model 2 cross validation data – LOO (from Figure 4B). S13
Figure S4 – All Docking scores versus pEC_{50}. S13
Figure S5 - Docking scores (below 6 kcal mol^{-1}) versus pEC_{50}. S14
Figure S6 - Human TTR and T_4 run1 RMSD. S15

Figure S8 - Human TTR and T_4 run3 RMSD. S16
Figure S9 - Human TTR and PFBA RMSD S16
Figure S10 - Human TTR and PFHxA RMSD. S17
Figure S11 - Human TTR and PFHpA RMSD. S17
Figure S12 - Human TTR and PFOA RMSD. S18
Figure S13 - Human TTR and PFNA RMSD. S18
Figure S14 - Human TTR and PFDA RMSD. S19
Figure S15 - Human TTR and ADONA RMSD. S19
Figure S16 - Human TTR and GenX RMSD. S20
Figure S17 - Human TTR and PFBS RMSD. S20
Figure S18 - Human TTR and PFHxS RMSD. S21
Figure S19 - Human TTR and PFHpS RMSD. S21
Figure S20 - Human TTR and PFOS RMSD. S22
Figure S21 - Rat TTR and T_4 run1 RMSD. S22
Figure S22 - Rat TTR and T_4 run2 RMSD. S23
Figure S23 - Rat TTR and T_4 run3 RMSD. S23
Figure S24 - Rat TTR and PFBA RMSD. S24
Figure S25 - Rat TTR and PFHxA RMSD. S24
Figure S26 - Rat TTR and PFHpA RMSD. S25
Figure S27 - Rat TTR and PFOA RMSD. S25
Figure S29 - Rat TTR and PFDA RMSD. S26
Figure S30 - Rat TTR and PFBS RMSD. S27
Figure S31 - Rat TTR and PFHxS RMSD. S27
Figure S32 - Rat TTR and PFHpS RMSD. S28
Figure S33 - Rat TTR and PFOS RMSD. S28
Figure S34 - PFAS/rTTR free binding energies correlate with *in vivo* points of departure (PODs) for serum free T_4 decreases in male rats. S29
Figure S35 - Toxicokinetic modeled vs. measured serum concentrations of PFAS in male rats. S30
References S30

Table S4 – Toxicokinetic models and parameters used to estimate serum concentrations

PFAS	Sex	Model Type	Volume of Distribution (L/kg)	Elimination Half-Life (hr)	Absorption rate coefficient (Unitless)
PFPrA	F	Absorption	0.187[a]	1.76[a]	4.15[a]
PFPrA	M	Absorption	0.253[a]	9.22[a]	3.04[a]
PFBA	F	Absorption	0.187[b]	1.76[b]	4.15[b]
PFBA	M	Absorption	0.253 [b]	9.22[b]	3.04[b]
PFOS	F	Absorption-independent	0.538[c]	6,770[c]	NA
PFOS	M	PBPK[d]	NA[d]	NA[d]	NA[d]
PFOA	F	Absorption-independent	0.211[e]	2.23[f]	NA
PFOA	M	Absorption	0.339[e]	39.4[h]	0.45[g]
PFNA	F	Absorption-independent	0.243[e]	50.4[i]	NA
PFNA	M	Absorption-independent	0.287 [e]	672[j]	NA
PFHxS	F	Absorption-independent	0.126[k]	21.1[h]	NA
PFHxS	M	Absorption-independent	0.275 [k]	382[l]	NA
PFHxA	F	Absorption-independent	1.48[a]	2.7[a]	NA
PFHxA	M	Absorption-independent	1.31[a]	5.4[a]	NA
PFDA	F	Absorption-independent	0.441[e]	569[f]	NA
PFDA	M	Absorption-independent	0.348[e]	264[m]	NA
PFBS	F	Absorption-independent	0.351	3.96	NA
PFBS	M	Absorption	0.33	2.1	7

[a]Toxicokinetic data for PFPRA are unavailable, therefore, toxicokinetic data for the closely-related linear short-chain (4-carbon) perfluorocarboxylic acid (i.e., PFBA) was used based on the approach implemented by the US EPA (2023)[1], using data from Chang *et al.*[2]
[b]Chang *et al.*[2]
[c]Model estimates reported in Table 4-4 of US EPA (2024).[3]
[d]PBPK model from Chou *et al.*[4]
[e]Ohmori *et al.* [5]
[f]Dzierlenga *et al.*[6]
[g]ATSDR [7]
[h]Kim *et al.*[8]
[i]De Silva *et al.*[9]
[j]Tatum-Gibbs[10]
[k]Sundstrom *et al.*[11]
[l]Benskin *et al.*[12]
[m]D'eon *et al.*[13]

Table S5 – QSAR comparison: Models that predict TTR/PFAS binding potencies

Reference Experimental set	Chemical coverage	Descriptors	Model/Method/Performance	Significant (top) descriptors

Weiss *et al.* (2009) [14]<br><br>TTR-PFAS binding data from same study	Excluded PFBS. 23 PFAS used in the training set. No separate validation set	56 calculated molecular descriptors (MOE software)	Regression model Partial Least Squares (PLS).<br>R^2 0.61 (training set)<br>R^2 0.41 (cross-validation)	Size-related connectivity indices and diameter and radius, and surface-describing descriptors related to total and negative polar surface area
Kovarich *et al.* (2012) [15]<br>TTR-PFAS binding data from Weiss et al. (2009)[14]	24 PFAS used in the model. Training set 19 chemicals; validation set 5 chemicals	1-, 2-, and 3-dimensional molecular descriptors (444). 4 models, each model has two descriptors. (DRAGON software)	Classification model K-nearest neighbors (K-NN)	AMW, HATS6m.; nH, HATS6m; nH, F06[C-O]; T(F..F), HATS6m
Papa *et al.* (2012)[16]<br><br>TTR-PFAS binding data from Weiss et al. (2009)[14]	PFAS + brominated flame retardants. Divided data into different classes. Training set included 11 PFAS, validation set included 4 PFAS.	500 mono-, bi- and tridimensional molecular descriptors (DRAGON software).	Regression model (3 descriptors)<br>Least square regression.<br>Training R^2 0.89, RMSE 0.42<br>Validation RMSE 0.34<br>Classification model (3 descriptors)<br>Accuracy training 84%, validation 81%<br>5 misclassified PFAS: perfluorotetradecanoic acid, perfluorohexanoic acid, perfluorododecanoic acid,<br>2-perfluorohexyl ethanol and perfluorooctane sulfonic acid. DoA: leverage approach, the Williams plot and the Insubria graph – all values fit within the domain of application	Regression model: FO7[C-O], nArOH<br>Classification model: nArOH, (F03[Br-Br]), HATS6m

Zhang *et al.* (2015)[17]<br><br>TTR-PFAS binding data from Weiss et al. (2009)[14]	Structurally diverse dust contaminants<br>Training set: 178<br>Validation set: 44 (23 – other chemicals and 4 PFAS, respectively)	132 2D molecular descriptors (MOE software)	Classification model (14 descriptors)<br>K-nearest neighbors (kNN), Partial Least Squares-Discriminant Analysis (PLS-DA), C-SVC from a Library for Support Vector Machines (LIBSVM)<br>Accuracy (with best method – kNN): 0.88 (cross validation), 0.82 (external validation)	14 chemical descriptors - hydrophobicity, van der Waals surface area, partial charge, and numbers of hydroxyl groups and halogen atoms
Kar *et al.* (2017)[18]<br><br>TTR-PFAS binding data from Weiss et al. (2009)[14]	Classification model: Training set: 16, Validation set: 8. Regression model: 10 (training), 5 (validation)	Molecular Descriptors (DRAGON software)<br><br>Does not provide full number of descriptors	Classification model (3 descriptors)<br>Accuracy: 93.75% (training), 100% (validation)<br>MLR model<br>R^2 0.87 (training)<br>DoA: Euclidean distance and the standardization based technique	AM1 descriptors (semi-empirical method): HOMO/LUMO gap, geometrical structures
Evangelista *et al.* (2024)[19]<br><br>TTR-PFAS binding data from Weiss et al. (2009)[14] and Ren et al. (2016)[20]	50 and 137 compounds (including PFAS) are grouped by binding study substrate.	7185 molecular descriptors (PaDEL).	FITC-T4 MLR (3 descriptors).R^2 0.85 (training), RMSE 0.38 (training), 0.4 (validation). This model was also redeveloped to combine all data.<br>RLBA MLR (6 descriptors)<br>R^2 0.81 (training)<br>RMSE 0.52 (training), 0.66 (validation).<br>DoA: leverage approach and Williams plot	FITC-T4 model: naasC, SpMin4_Bhs, VE3_Dzs<br><br>RLBA model: PubchemFP590, SpMax1_Bhe, PubchemFP18, GATS5c, AATSC1e, AATS4v
Zhao *et al.* (2025)[21]<br><br>Model is based on in silico predictions of TTR-PFAS	Predicted TTR-PFAS binding (in silico) for 430 PFAS (124 training, 287 validation)	354 molecular 2D and 3D descriptors (MOE software)	MLR model (72 descriptors)<br>R^2 0.89	PEOE_RPC-, E_vdw, MNDO_LUMO, and vsurf

binding from the same study				
Sosnowska *et al.* (2025)[22]<br><br>Based on TTR-TRβ-CALUX bioassay data (same study)	45 PFAS (4:1 training to validation split)<br><br>In multiple regression models: randomly assigned 30% PFAS plus compounds with maximum and minimum activity	Molecular descriptors including 1D and 2D (Alvadesc software)	Classification model (two descriptors) Accuracy 94% (training) 91% (validation)<br><br>MLR model (3 descriptors) R^2 0.77 RMSE 0.555 DoA: Williams plot<br><br>Multiple regression models (MRM) suite	Classification model: SM4_D and GATS3m MLR model: AMW, GATS7p, B10[F-F]<br><br>MRM suite, 55 molecular descriptors combined, standout descriptors: JG110, ATSC7c, MATS6i
Evangelista et al. (2025)[23]<br><br>TTR-PFAS binding data from Degitz *et al.* (2024)[24]	Regression model Training: 43 PFAS Validation: 20 PFAS Classification model Training: 82 PFAS Validation: 39 PFAS	Fingerprints, 1D and 2D molecular descriptors (PaDEL)	Regression model (3 descriptors) Multiple linear regression Training R^2 0.81 Validation R^2 0.77 Classification model (4 descriptors) Linear discriminant analysis Accuracy 89% (training), 85% (validation)	Regression: piPC5, GGI9, AATSC0e Classification: autocorrelation descriptors (GATS3e, ATSC6p, GATS8m), MIC2
Makarov *et al.* (2025)[25]<br><br>Tox24 Challenge TTR binding (winning team)<br><br>TTR-PFAS binding data from Degitz *et al.* (2024)[24] and TTR Tox21[26, 27]	Regression model Train set = 1512 Blind set = 300<br><br>RMSE=20.3	Multiple descriptor sets retrieved through OCHEM (including Mold2, ALogPS, OEstate, Mordred, PaDEL, MACCs)	Ensemble of predictions from four separate models: catBoost with ALogPS and OEstate descriptors, catBoost with Mold2 Descriptors, Transformer CNF, and Transformer CNN.<br><br>Validation (best model)<br><br>R^2 0.70 ± 0.04 All data 0.65 ± 0.02	For best model: ALogPS_logP, Se1C3O1a, ALogPS_logS, PSA, SsCH3, SeaC3C3aa, SeaC2C3aa, MW, aCNOS, DONORS

Table S6 – Top descriptors for QSAR models 1 and 2 (listed in the order of decreasing importance)

QSAR model 1	QSAR model 2
b_single	a_count
a_count	b_single
a_nC	a_nC
KierA1	E_vdw
GCUT_SLOGP_3	a_nO
a_nO	GCUT_SLOGP_3
lip_violation	TPSA
balabanJ	balabanJ
TPSA	lip_violation

Table S8 – Averaged binding energies for human and rat MD models

Molecules studied	Binding affinities human model (kcal/mol)	Binding affinities rat model binding pocket 1 (kcal/mol)	pEC_{50s}
T_4	-40.01±4.34	-35.95±4.02	0.68
PFBS	-11.61±3.50	-14.75±3.86	0.11
PFHxS	-19.14±2.89	-21.74±2.87	0.74
PFHpS	-24.21±3.40	-19.23±3.69	1.17
PFOS	-25.25±2.99	-21.55±3.66	0.27
PFBA	-11.65±4.40	-6.25±4.24	-2.24
PFHxA	-15.85±3.66	-17.47±3.12	-0.15
PFHpA	-19.34±2.70	-17.86±2.64	0.40
PFOA	-22.42±3.65	-18.68±4.57	0.36
PFNA	-25.46±2.98	-27.30±3.40	0.36
PFDA	-20.38±4.32	-22.79±3.69	0.15
ADONA	-21.14±3.70	-	0.68
GenX	-17.36±3.73	-	0.11

Table S9 – Measured serum concentration data for PFAS in male rats used to test performance of toxicokinetic models

Study	PFAS	Doses Administered (mg/kg-d)	Measured Serum Concentrations (mg/L)
Butenhoff *et al* 2012 [28]	PFBA	5.301, 25.4, 130.2	24.65, 38.04, 82.2
NTP 2022 [29]	PFBS	62.6, 125, 250, 500, 1000	0.09, 2.222, 5.366, 12.43, 43.16
NTP 2022 [30]	PFDA	0.156, 0.312, 0.625, 1.25, 2.5	8.505, 23.03, 42.72, 101.6, 259.4

NTP 2022 [29]	PFHxS	0.625, 1.25, 2.5, 5, 10	66.76, 92.08, 129, 161.7, 198.6
NTP 2022 [30]	PFNA	0.625, 1.25, 2.5	56.73, 161, 380
NTP 2022 [29]	PFOS	0.312, 0.625, 1.25, 2.5, 5	23.73, 51.56, 94.26, 173.7, 318.2
NTP 2022 [30]	PFHxA	62.6, 125, 250, 500, 1000	0.378, 0.503, 1.297, 3.339, 10.9
NTP 2022 [30]	PFOA	0.625, 1.25, 2.5, 5, 10	50.69, 73.48, 95.43, 110.7, 148.6

Table S10 – Model performance metrics for best-performing toxicokinetic models for each PFAS chemical for male rats

	Model Type	R^2	RMSE (mg/L)	Additive Bias (mg/L)	Multiplicative Bias (unitless ratio)
PFHxA	Absorption-independent	0.9646	2.77	-1.43	0.771
PFBS	Bi-phasic	0.9646	12.20	-11.84	0.1460
PFBA	Absorption	0.9946	44.78	-41.25	0.08022
PFOS	PBPK	0.9975	66.28	-54.53	0.5488
PFDA	Absorption-independent	0.9886	82.6	-53.73	0.4607
PFOA	Absorption	0.9248	83.65	-80.95	0.1065
PFNA	Absorption-independent	0.9999	129.5	-100.3	0.5601
PFHxS	Absorption-independent	0.8967	166.5	83.81	1.142

METHODOLOGY

hTTR and rTTR QSAR models

QSAR model 1 equation (S1): -15.847+6.270*a_count/SD(a_count)–1.253*a_nC/SD(a_nC)+ 0.660*a_nO/SD(a_nO)+0.375*BalabanJ/SD(BalabanJ)+0.947*GCUT_SLOP_3/SD(GCUT_SLOP_3)+1.250*KierA1/SD(KierA1)–0.408*lip_violation/SD(lip_violation)– 0.317*TPSA/SD(TPSA) – 6.883 *b_single /SD(b_single)

QSAR model 2 equation (S2): -13.029 – 2.934*a_nC/SD(a_nC) + 9.808*a_count/SD(a_count) + 0.851* a_nO/SD(a_nO) + 0.419*BalabanJ/SD(BalabanJ) – 9.163*b_single/SD(b_single) –

0.146* lip_violation/SD(lip_violation) + 1.312*E_vdw/SD(E_vdw) – 0.613*TPSA/SD(TPSA) + 0.665* GCUT_SLOP_3/SD(GCUT_SLOP_3)

hTTR and rTTR docking models

The Extended Hückel Theory (EHT) pharmacophore model was used with an essential feature (hydrogen bonding acceptor) for both human and rat models.[31, 32] Both pharmacophore models included an essential hydrogen bonding acceptor feature to Lys15, with a radius of 1.8 and 1.6 angstroms, for human and rat respectively as implemented in MOE 2022 (Figs. 3B, D). For the rat model, an anionic feature was additionally included which aids in recovering a higher percentage of suitable docking poses. In docking, the EHT forcefield was used for minimization purposes.[31, 32] The London ΔG scoring function was used with pharmacophore scoring evaluating 100 placements, which were then refined to 10 poses with the generalized-Born volume integral/weighted surface (GBVI/WSA).[33] The poses were then analyzed, the best suiting pose selected, and the corresponding docking score collected.

Molecular dynamic simulations

Molecular dynamics simulations were performed with Amber 22 and the structural analysis was done with AmberTools 22.[34] The AM1-BCC charge scheme was selected to calculate the partial charges of these compounds based on the antechamber module, using gaff2.[35, 36] The ff14SB forcefield was utilized for both scaffolds (TTR proteins).[37] The protein-ligand complex was fitted into a 14 Å box, neutralized and ionized with 0.10 M of NaCl, using Joung and Cheatham parameters.[38] Six different harmonic potentials were selected for the minimization step, reducing the harmonic potential constraints in a stepwise fashion. Each individual system was then heated for 30 picoseconds (ps) from 100 to 300K. The SHAKE algorithm was selected to constrain hydrogen bonds and the particle-mesh Ewald method was considered to approximate long-range electrostatic interactions.[39]

Toxicokinetic modelling

PFAS toxicokinetic models were used based on their availability in literature. PBPK models were prioritized, followed by first-order single compartment models with an absorption phase, and lastly, absorption-independent first order single compartment models when absorption phase rate constants were unavailable. A PBPK model was only available for PFOS (i.e., Chou & Lin)[4], and absorption rate phase constants were only available for PFOA, PFBS (males only), and PFBA. A complete list of toxicokinetic parameters for all models used, including their sources, are available in Table S4. Default model parameters and code provided in Chou & Lin[4] were used to model PFOS serum concentrations.

When an estimate for the absorption rate constant (k_a; hr^{-1}) was available, the following equation was used to model the serum concentration of PFAS in the rat at time point (*t*):

Equation S3 $$C(t) = \frac{D \cdot F \cdot k_a}{V_d(k_a - k_e)} \cdot \left(\frac{e^{-k_e \cdot t}}{1-e^{-k_e \cdot \tau}} - \frac{e^{-k_a \cdot t}}{1-e^{-k_a \cdot \tau}}\right)$$

Where D is the body weight-normalized administered dose (mg/kg), *F* is the oral bioavailability fraction, which was assumed to be 100% for all PFAS in this assessment, (i.e., F = 1) (F was experimentally demonstrated to be 95-100% in male rats for all PFAS analyzed in Fig. 9; ATSDR [7], Huang *et al.*[40], Dzierlinga *et al.* 2019[41]) ; k_e is the elimination rate constant (hr^{-1}) – which is equivalent to ln(2) divided by the elimination half-life; V_d is the volume of distribution at steady-state (L/kg); τ is the dosing interval (hr) – which was 24 hr for all exposure scenarios.

When an estimate for the absorption rate constant (k_a; hr^{-1}) was not available for a given PFAS, the following simplified first-order, single-compartment model equation was used:

Equation S4 $$C(t) = \frac{D \cdot F}{V_d} \cdot \left(\frac{1-e^{-n \cdot k_e \cdot \tau}}{1-e^{-k_e \cdot \tau}}\right) \cdot e^{-k_e \cdot t}$$

Certain PFAS are known to follow biphasic kinetic profiles (e.g., GenX, PFOA, PFBS, PFOS) (Gomis *et al.*[42]). When toxicokinetic parameters were available for a biphasic model, serum concentrations were modelled using a two-phase approach consisting of an initial distribution (α) phase followed by a terminal elimination (β) phase, as described in Gomis et al. (2018). During the α phase, tissue distribution and absorption both contribute to the decline in serum concentration; during the β phase, elimination from the central compartment governs the serum concentration profile.

Equation S5 $$C_{\text{serum},\alpha}(t) = \frac{F \cdot \left(\frac{D}{U}\right) \cdot k_a}{Vd_0 \cdot (k_a - k_d)} \cdot \left(e^{-t \cdot k_d} - e^{-t \cdot k_a}\right), \quad t \in [0, t_{\alpha,\text{end}}]$$

Where *D* is the body weight-normalized administered dose (mg/kg); *U* is a unit conversion factor (24 hr/day) that normalizes the daily dose to an hourly basis; *F* is the oral bioavailability fraction (unitless; assumed = 1 as above); V_{d0} is the volume of distribution at time zero (L/kg); k_a is the absorption rate constant (hr^{-1}); k_d is the distribution rate constant into peripheral tissues (hr^{-1}); and $t_{\alpha,end}$ is the time corresponding to the end of the α phase (hr).

Equation S6 $$C_{\text{serum},\beta}(t) = C_{\text{serum},\alpha}(t_{\alpha,\text{end}}) \cdot e^{-t \cdot k_e}, \quad t \in [t_{\alpha,\text{end}}, t_{\text{end}}]$$

Where $C_{\alpha\_(t\alpha,end)}$ is the serum concentration at the end of the α phase (mg/L), which serves as the initial condition for the β phase; k_e is the terminal elimination rate constant (hr^{-1}); and t_{end} is the time corresponding to the end of the modelled exposure period (hr).

The area under the curve (AUC) of modelled serum concentrations was calculated using the trapezoidal rule, which approximates the area under a curve by dividing it into a series of trapezoids as in equation S7.

Equation S7 $$AUC = \sum_{i=1}^{n-1} \left(\frac{C_i + C_{i+1}}{2}\right) \times (t_{i+1} - t_i)$$

Where C_i and C_{i+1} are the concentrations at time points t_i and t_{i+1}, respectively; and t_i and t_{i+1} are consecutive time points. The area between each pair of points is calculated as the average of the concentrations, multiplied by the time interval between them.

The time-weighted average (TWA) of modelled serum concentrations was derived by dividing the AUC by the total time interval (i.e., t_0 to t_{final}), as shown in equation S8.

Equation S8 $$\text{TWA} = \frac{\text{AUC}}{\Delta t}$$

TK code in R is provided at https://github.com/OEHHA-NTES/Rat_TTR_PFAS_QSAR_Docking

TK model evaluation

Model performance was evaluated by comparing model-predicted serum concentrations (C) to measured serum concentrations ($\hat{C}$) (Table S9) using standard error metrics (i.e., root-mean squared error [RMSE], and R^2). In addition, model bias was calculated for the best-performing model, which (i.e., the model with the lowest RMSE within each chemical/species/sex/route grouping) as the mean signed error:

Equation S9 $$Bias = mean(\hat{C} - C)$$

Bias is expressed in the same units as serum concentration (mg/L). A bias of 0 mg/L indicates no systematic over- or under-prediction on average. Positive bias indicates systematic overprediction (predicted concentrations higher than measured), whereas negative bias indicates systematic underprediction. Because PFAS serum concentrations can span orders of magnitude, additive (mg/L) bias is most interpretable as an absolute error measure; when interpreting proportional error across broad concentration ranges, bias was also expressed on a unitless multiplicative scale (e.g., geometric mean ratio $\hat{C}/C$). Model performance for male rats for best-performing models for each PFAS chemical are presented in Table S10. Overall, the selected best-performing models showed strong agreement with measured serum concentrations (R^2 = 0.897–0.9999), with PBPK selected for PFOS, absorption-phase models selected for PFBA and PFOA, bi-phasic model selected for PFBS, and absorption-independent models selected for PFHxA, PFDA, PFNA, and PFHxS. Across chemicals, RMSE ranged from 2.77 mg/L (PFHxA) to 166.5 mg/L (PFHxS). Additive bias was negative for most PFAS (−1.43 to −100.3 mg/L), indicating a general tendency toward underprediction, and this was consistent with multiplicative bias ratios <1 for PFHxA, PFBA, PFOS, PFDA, PFOA, and PFNA (0.080–0.771). In contrast, PFHxS exhibited positive additive bias (83.8 mg/L) and a multiplicative bias >1 (1.142), indicating modest overprediction. Additionally, a modeled vs. measured serum concentration scatterplot for male rats is presented in Figure S35.

SUPPORTING FIGURES

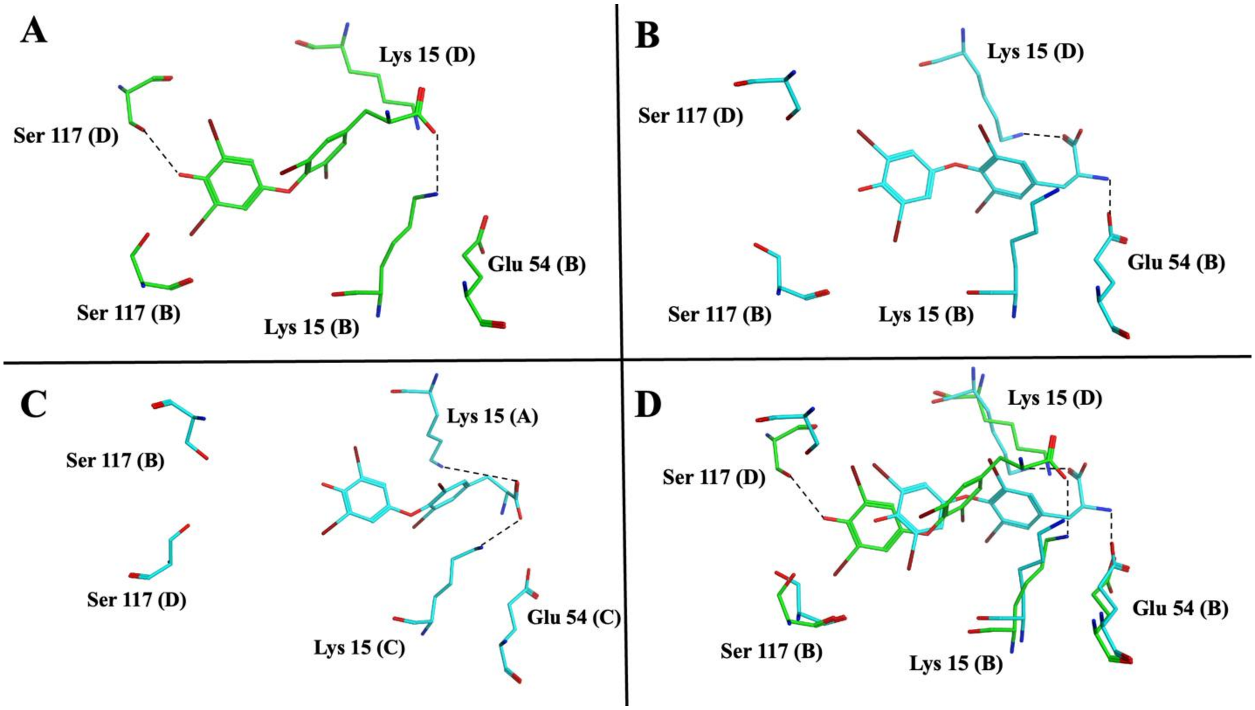

Figure S1 – Crystal structures for human and rat TTR. **A** - Human transthyretin ligand binding pocket, co-crystalized with T4 and relevant interactions, from PDB ID: 1ICT. **B** - Rat transthyretin crystal structure binding pocket 1, co-crystalized with T4 and relevant interactions from PDB ID: 1IE4. **C** - Rat transthyretin crystal structure binding pocket 2, co-crystalized with T4 and relevant interactions from PDB ID: 1IE4. **D** - Overlap of binding pockets 1 from human and rat crystal structure.

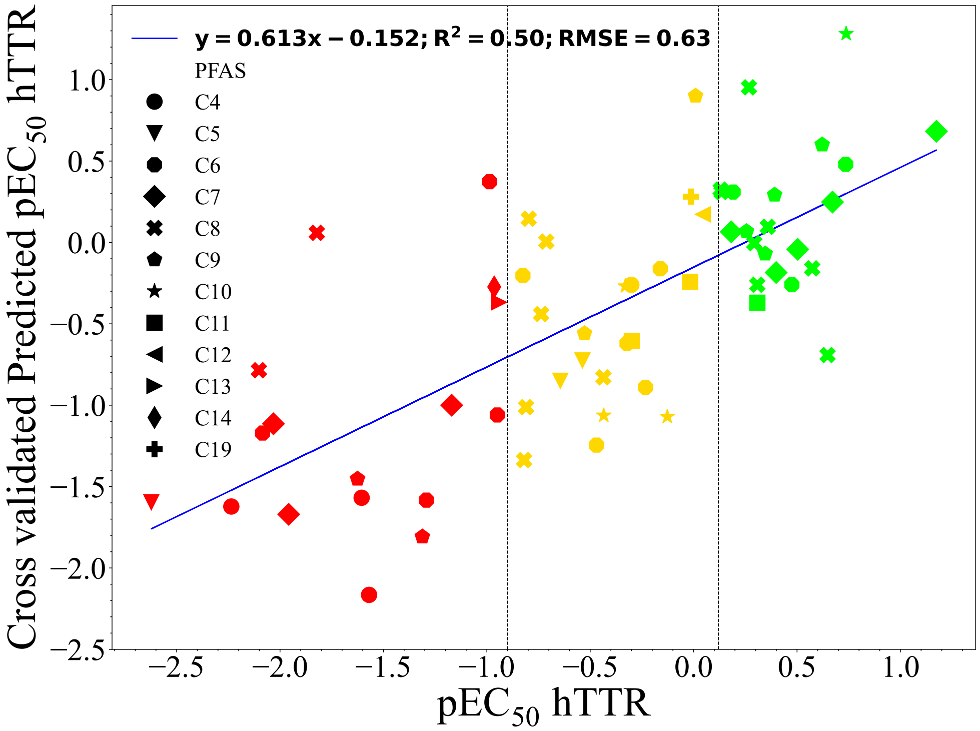

Figure S2 – QSAR model 1 cross validation data – LOO (from Figure 4A).

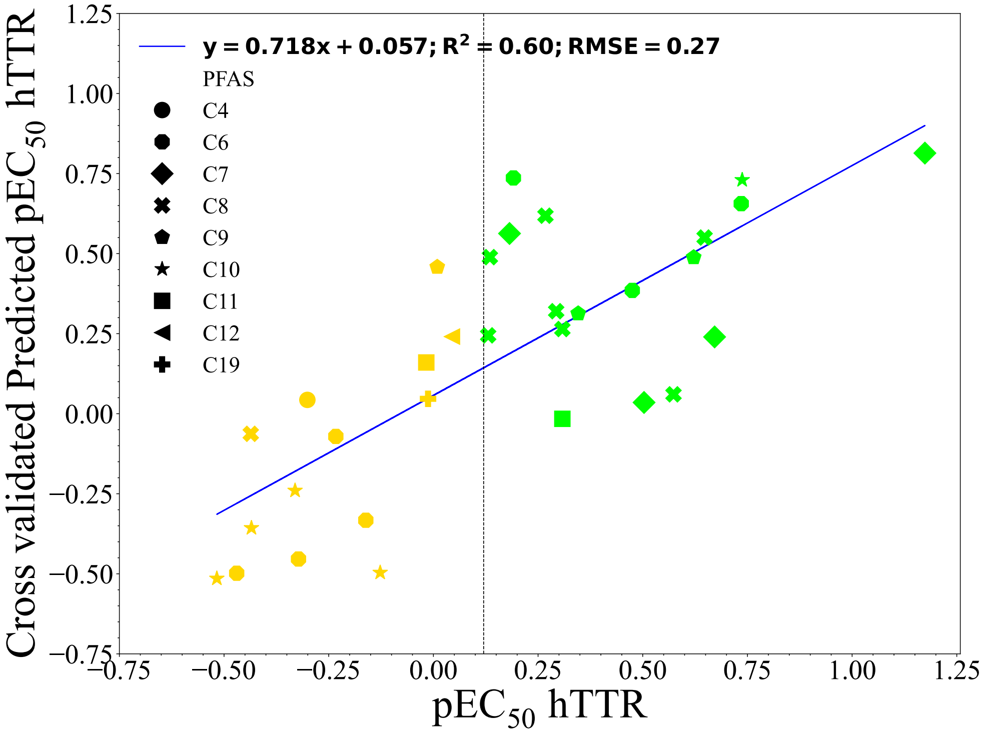

Figure S3 – QSAR model 2 cross validation data – LOO (from Figure 4B).

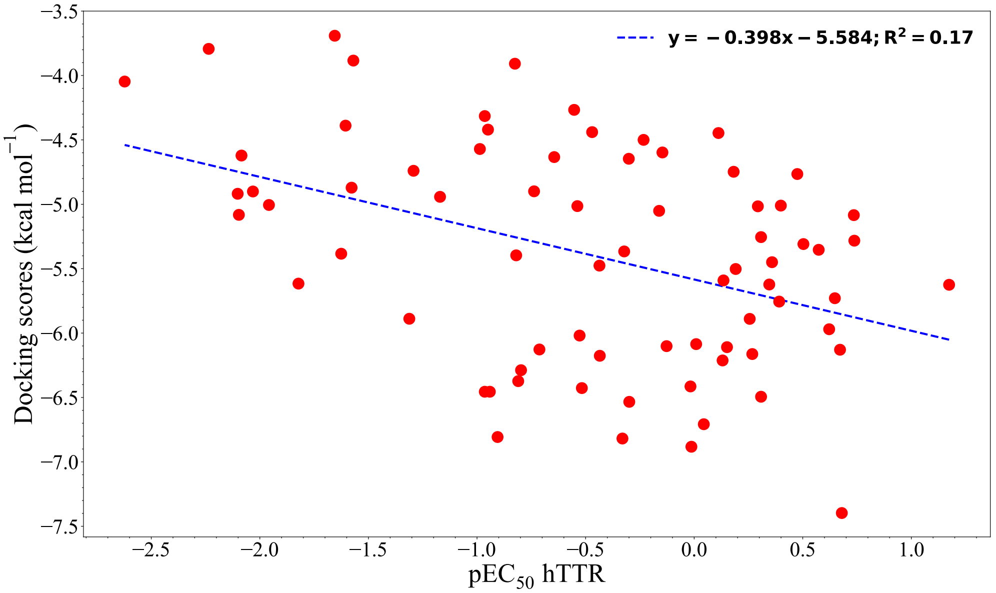

Figure S4 – All Docking scores versus pEC$_{50}$.

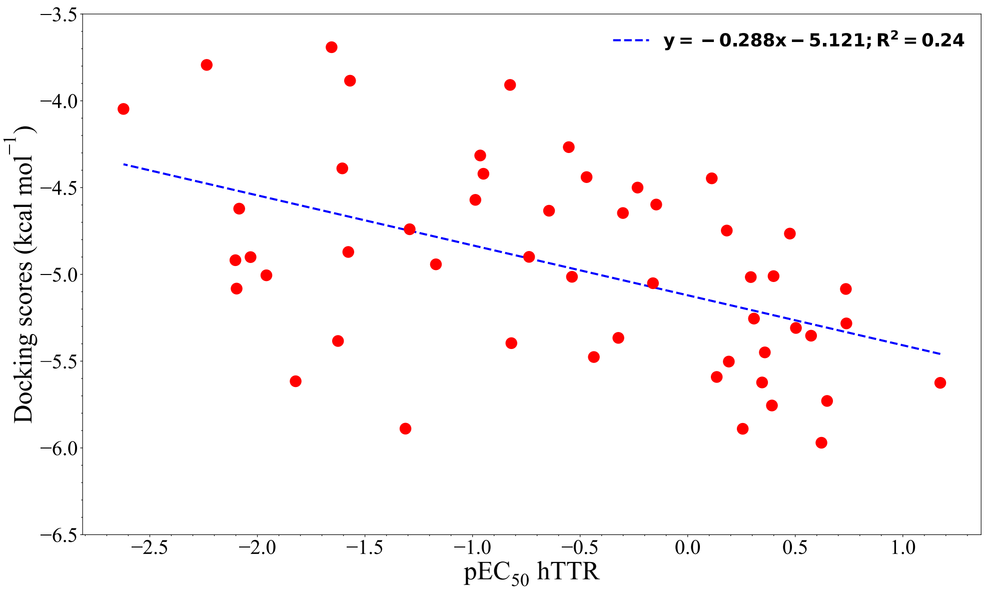

Figure S5 - Docking scores (below 6 kcal mol^{-1}) versus pEC$_{50}$.

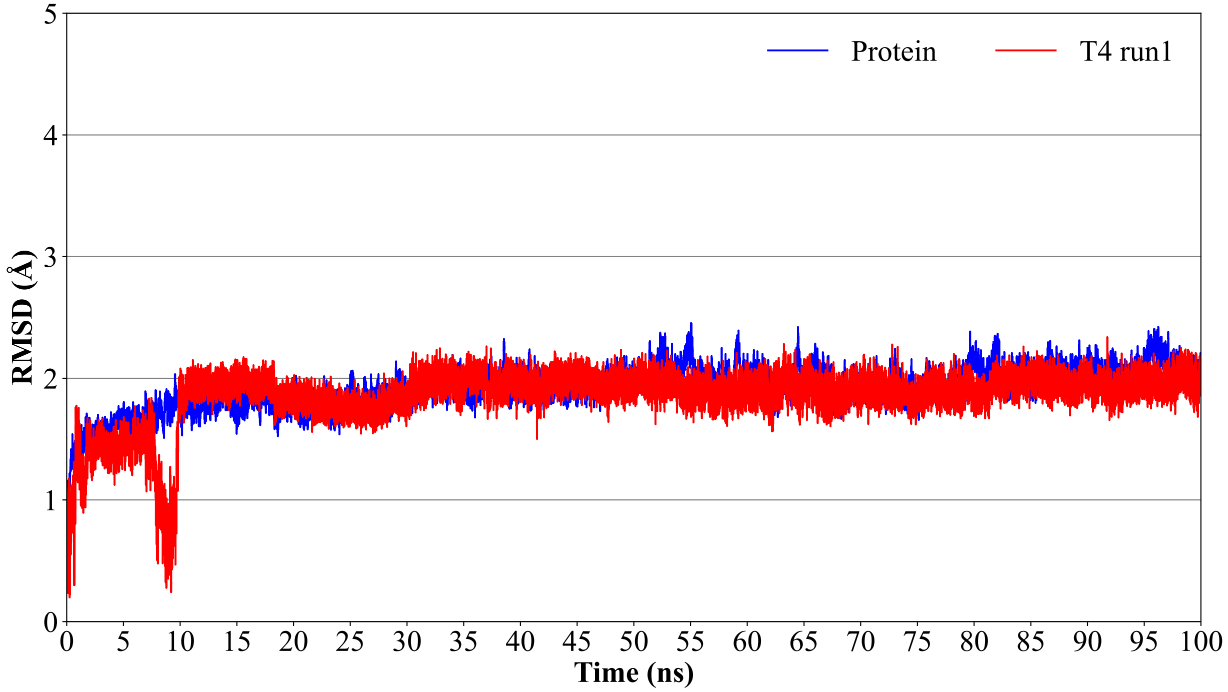

Figure S6 - Human TTR and T₄ run1 RMSD.

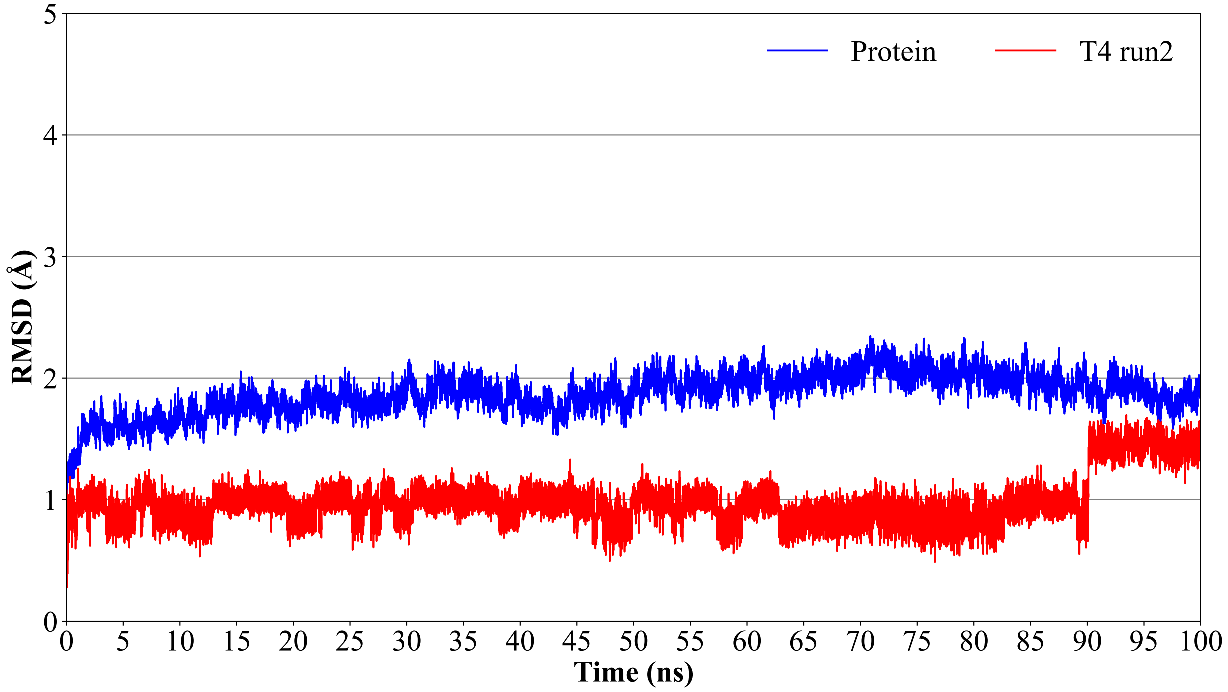

Figure S7 - Human TTR and T₄ run2 RMSD.

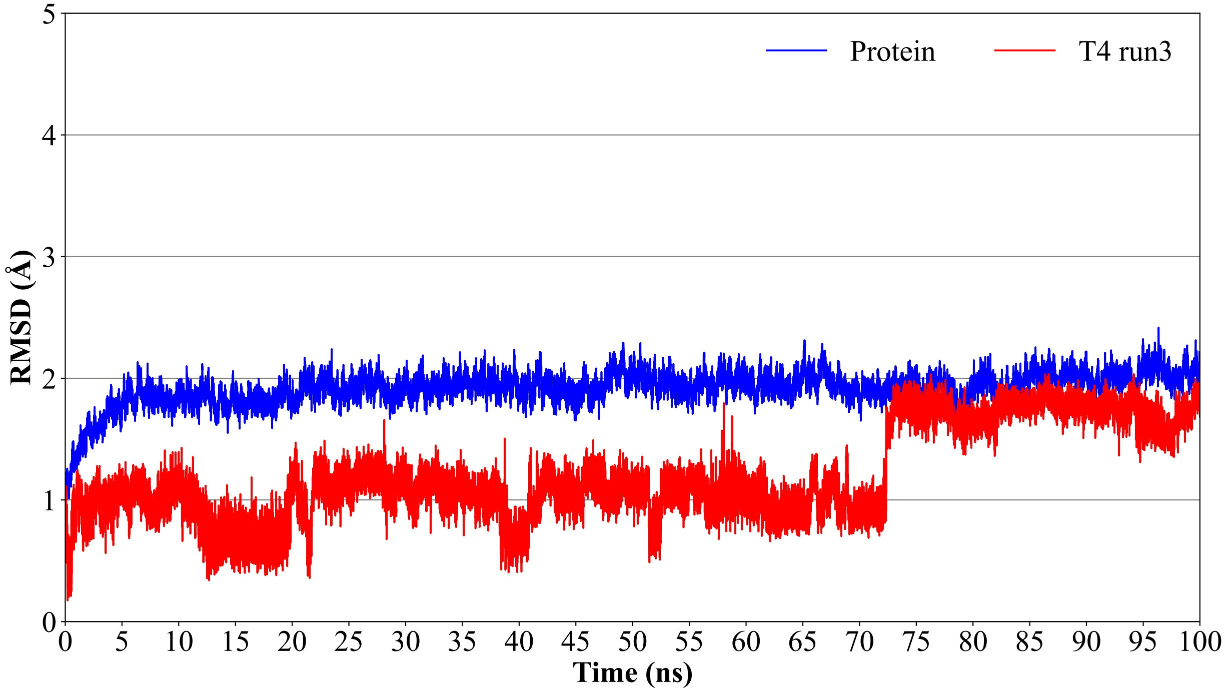

Figure S8 - Human TTR and T_4 run3 RMSD.

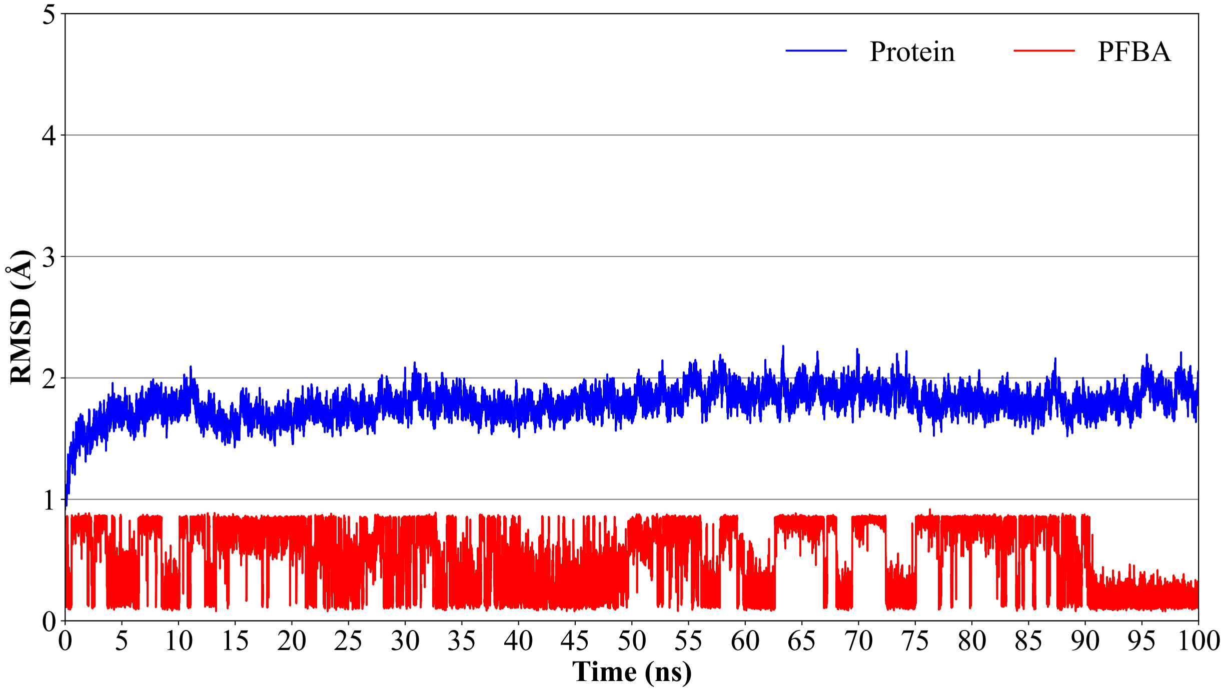

Figure S9 - Human TTR and PFBA RMSD

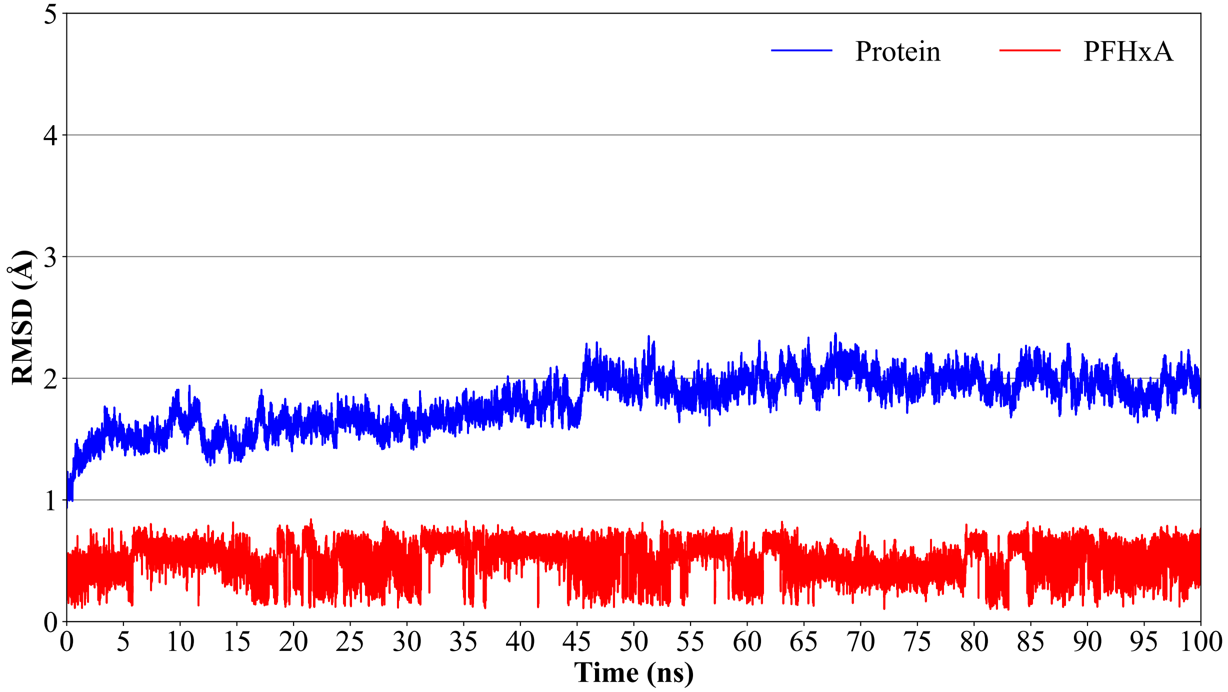

Figure S10 - Human TTR and PFHxA RMSD.

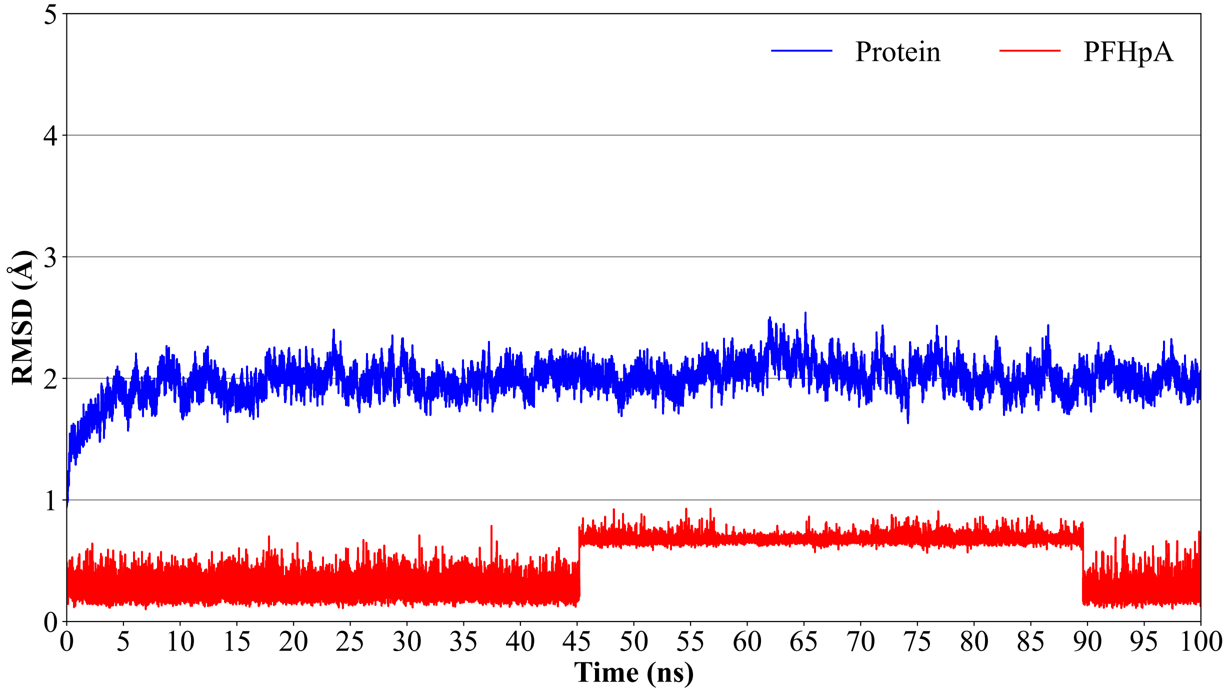

Figure S11 - Human TTR and PFHpA RMSD.

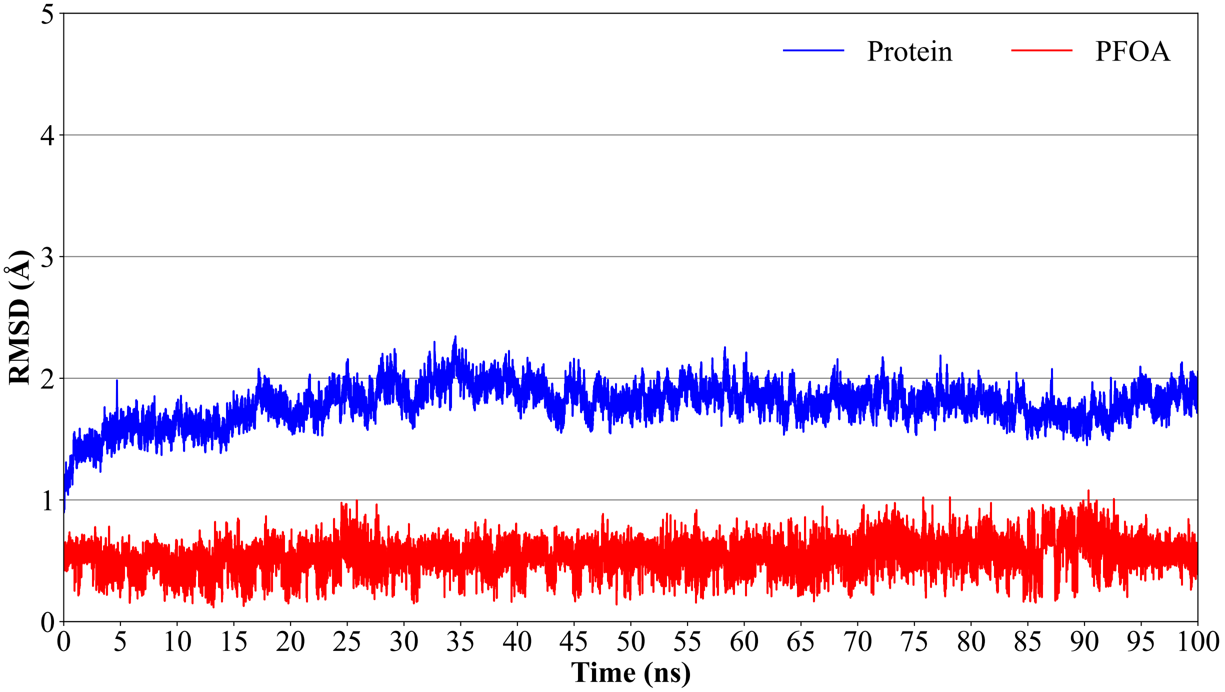

Figure S12 - Human TTR and PFOA RMSD.

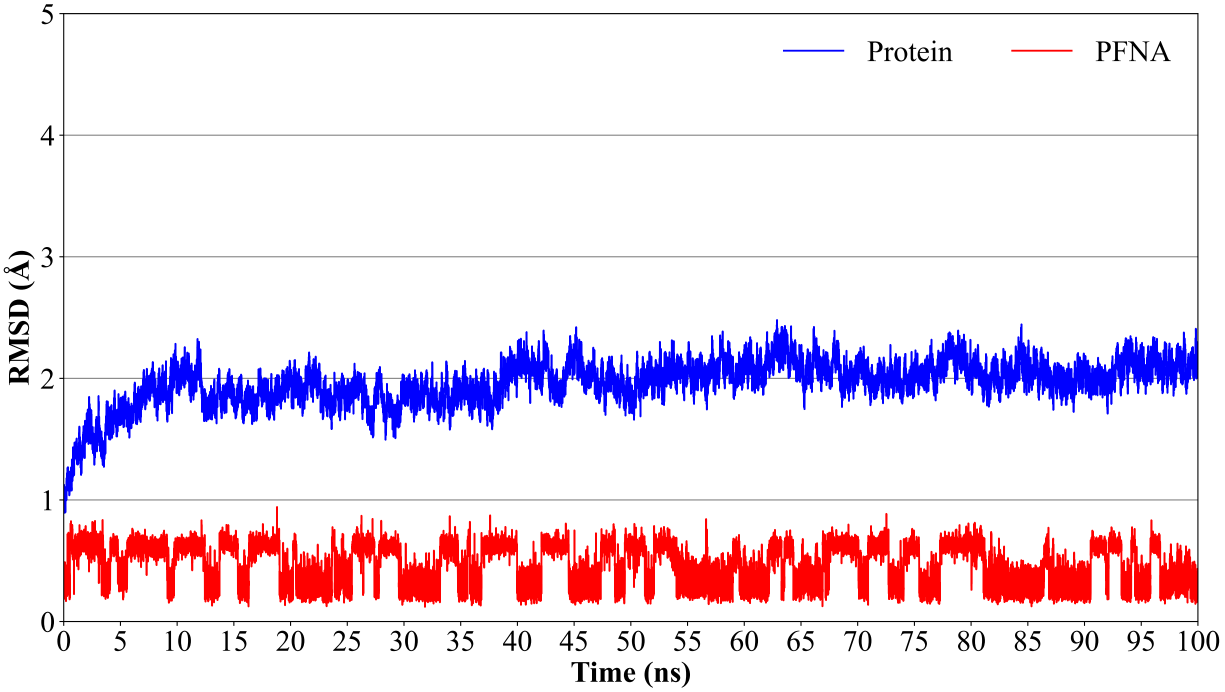

Figure S13 - Human TTR and PFNA RMSD.

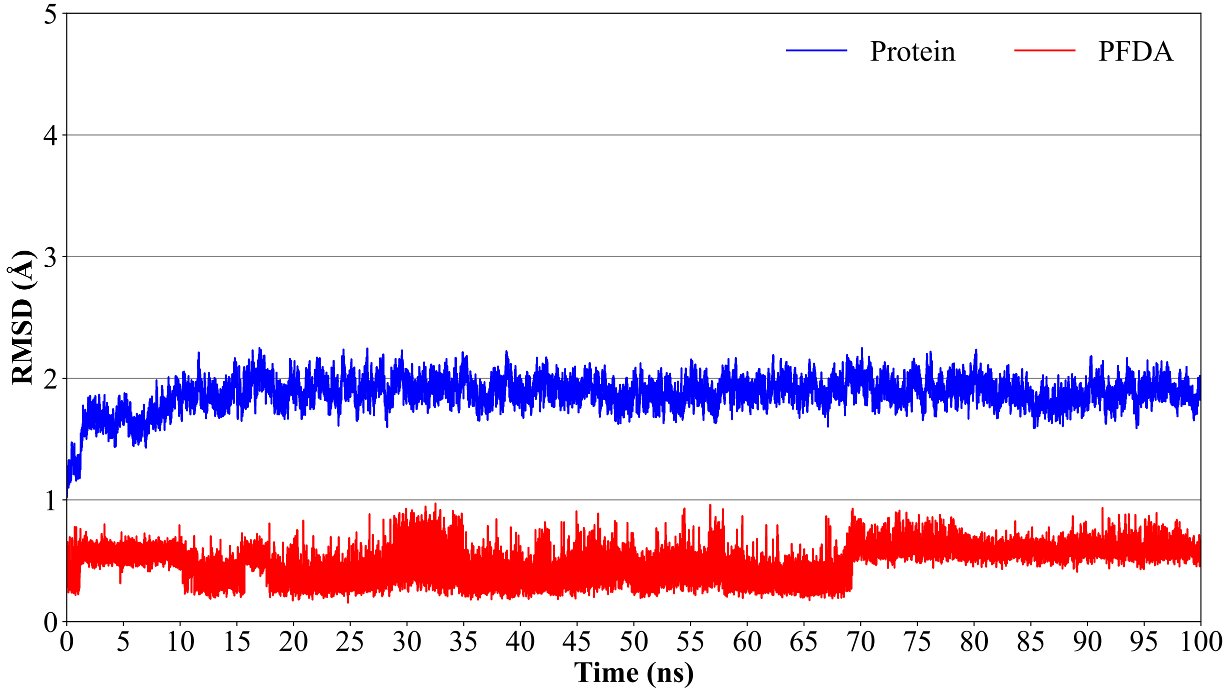

Figure S14 - Human TTR and PFDA RMSD.

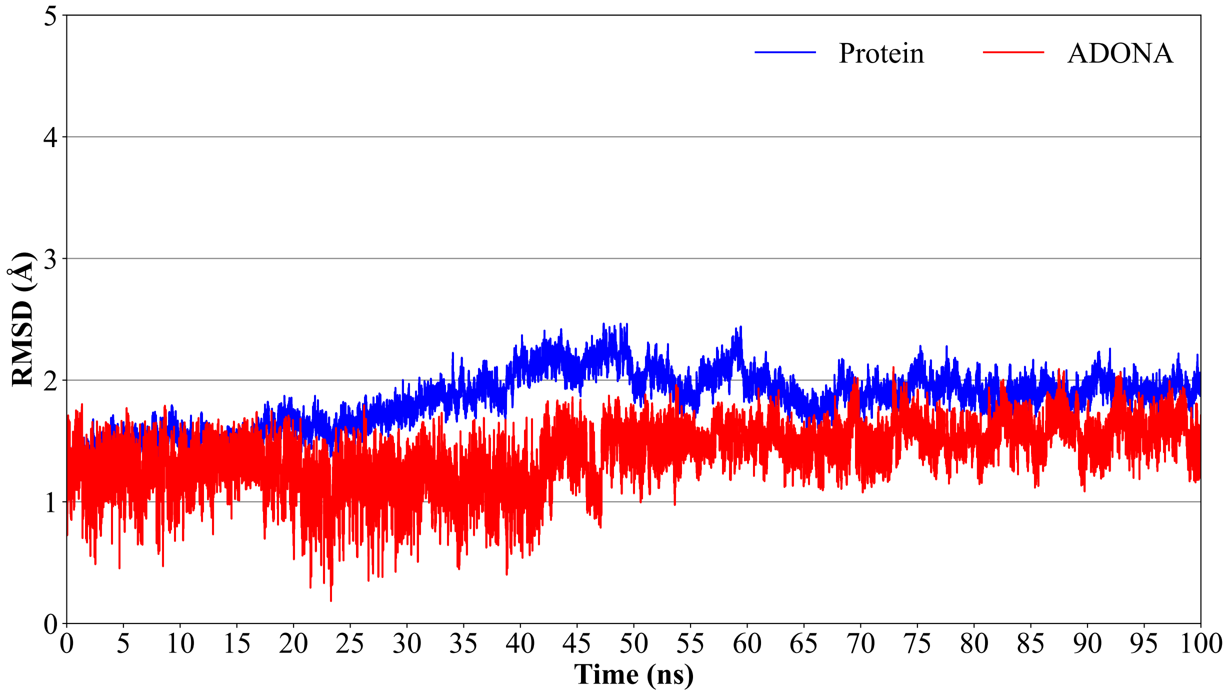

Figure S15 - Human TTR and ADONA RMSD.

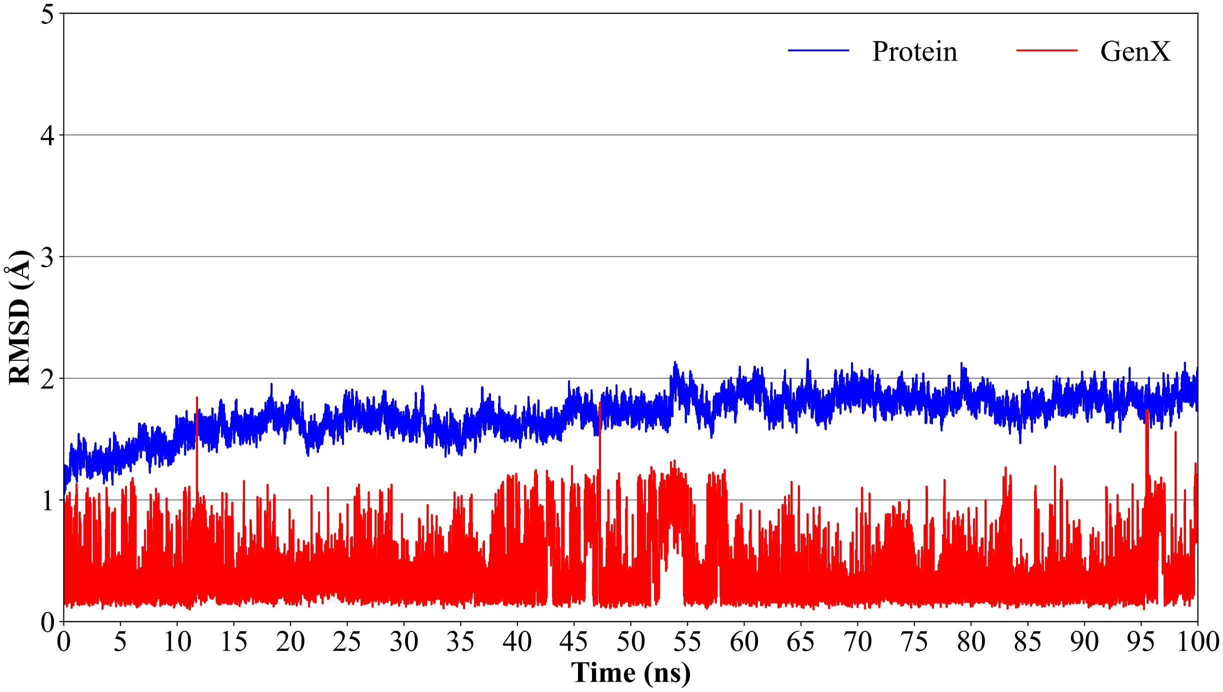

Figure S16 - Human TTR and GenX RMSD.

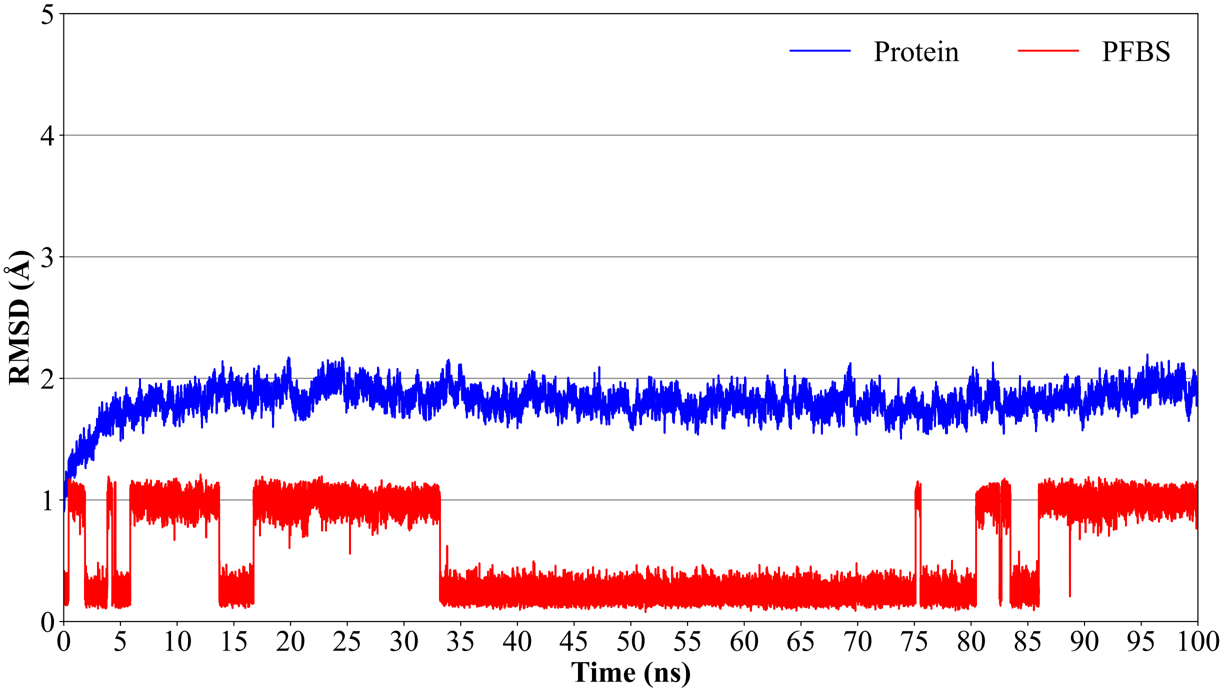

Figure S17 - Human TTR and PFBS RMSD.

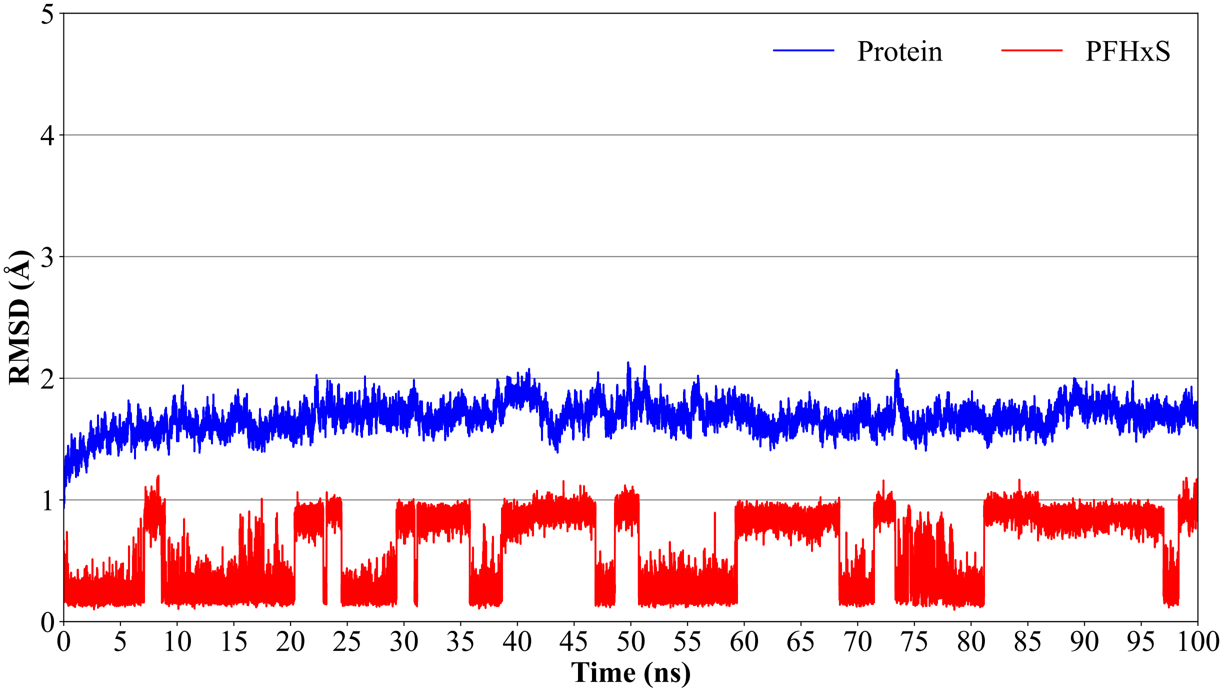

Figure S18 - Human TTR and PFHxS RMSD.

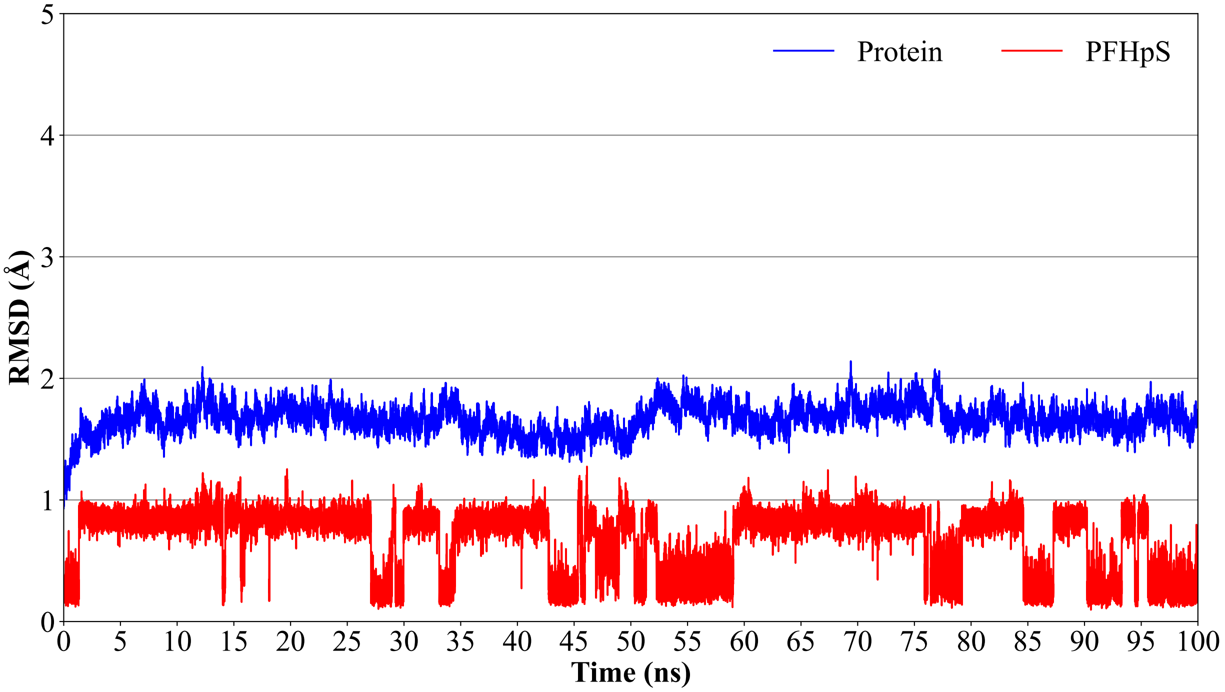

Figure S19 - Human TTR and PFHpS RMSD.

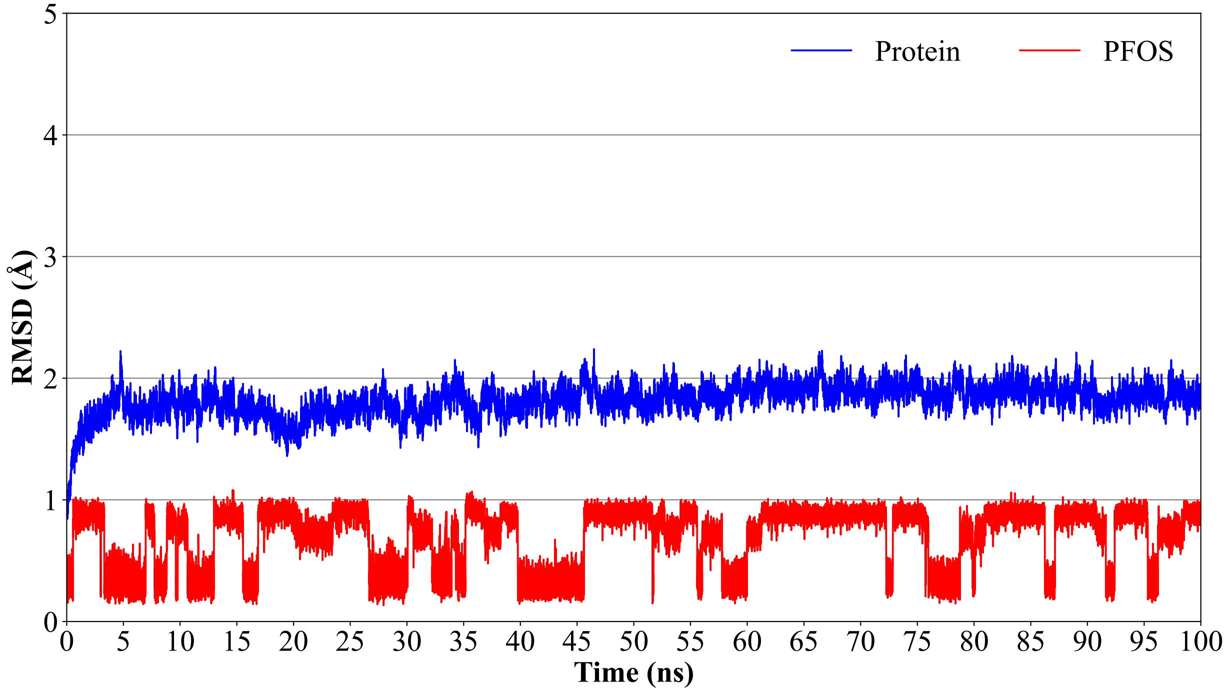

Figure S20 - Human TTR and PFOS RMSD.

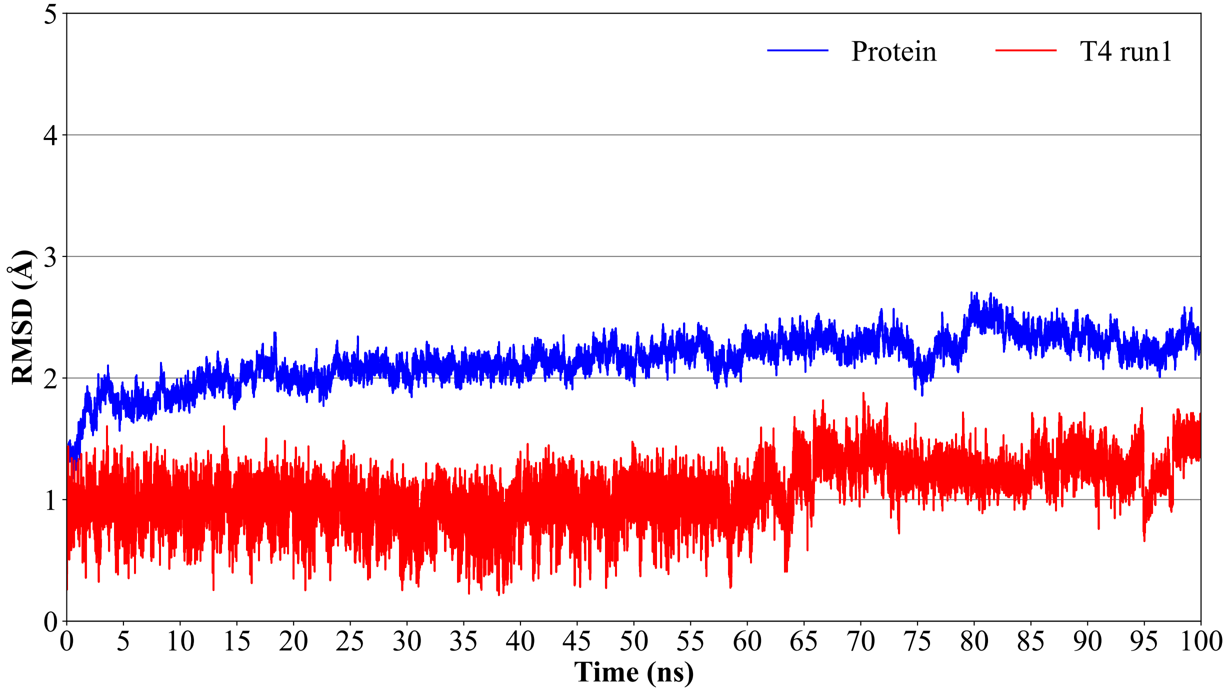

Figure S21 - Rat TTR and T_4 run1 RMSD.

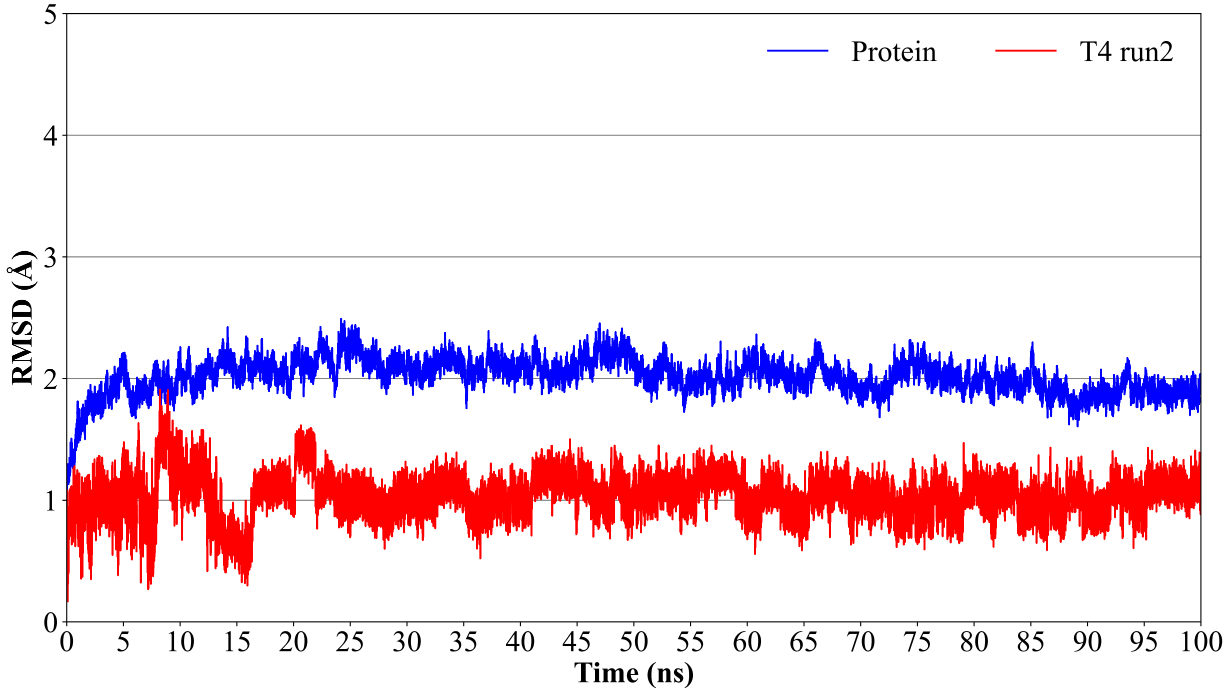

Figure S22 - Rat TTR and T_4 run2 RMSD.

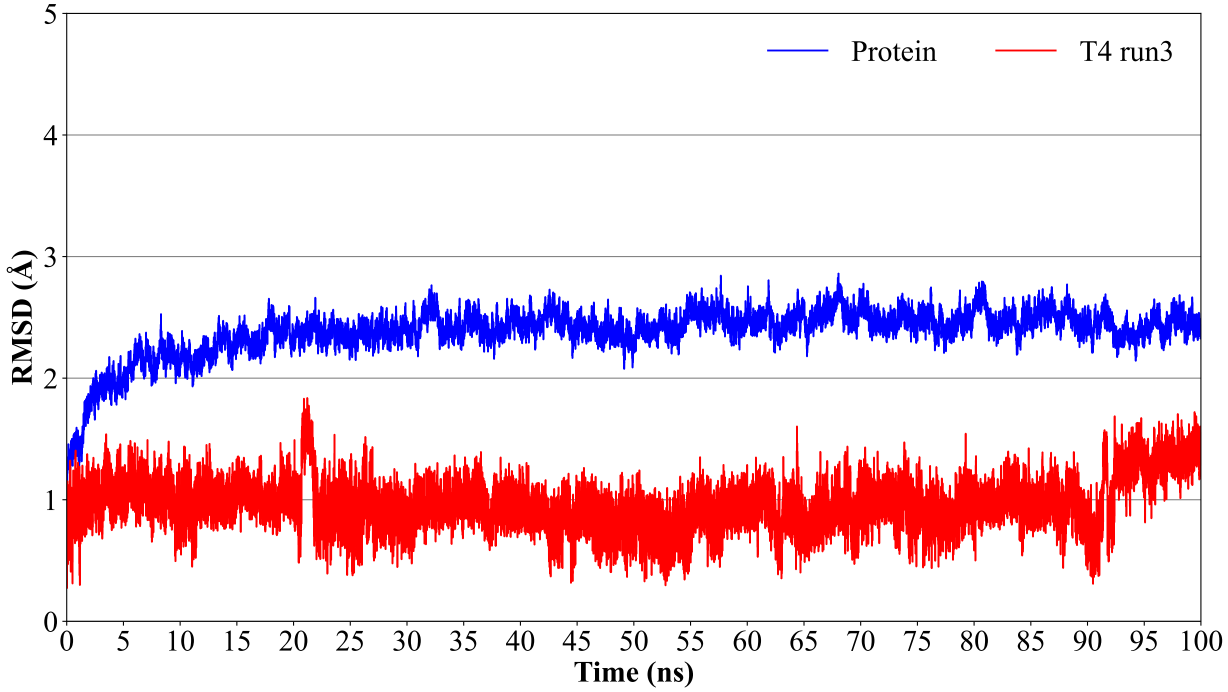

Figure S23 - Rat TTR and T_4 run3 RMSD.

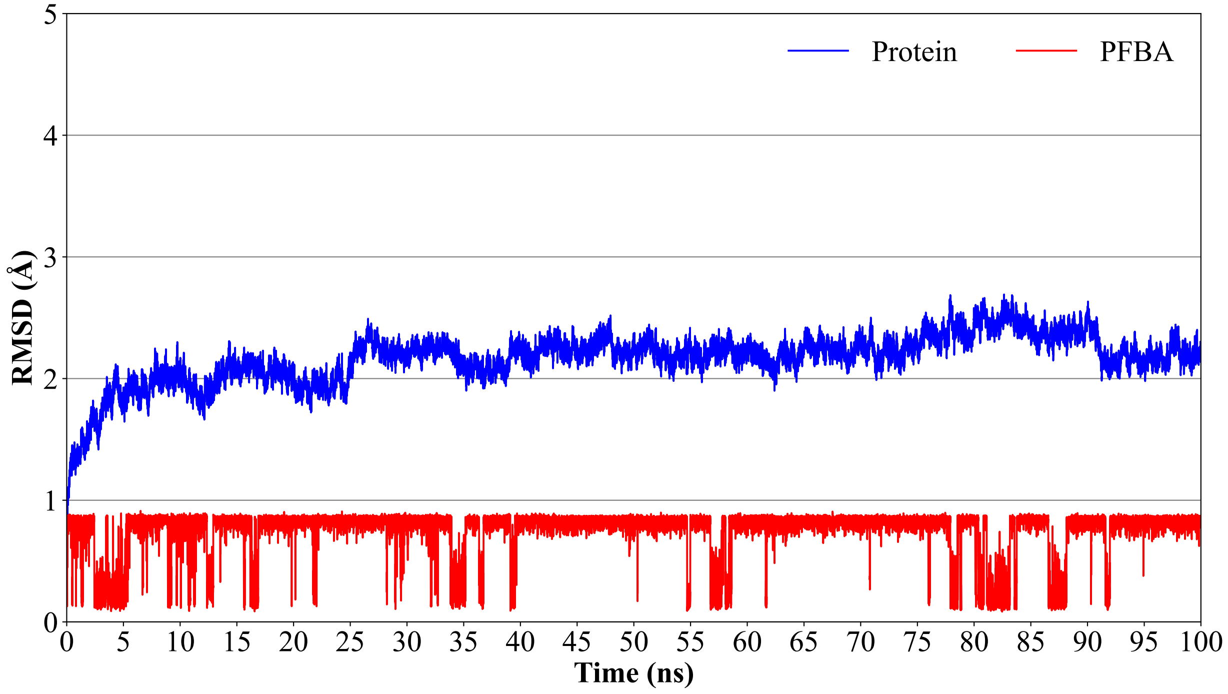

Figure S24 - Rat TTR and PFBA RMSD.

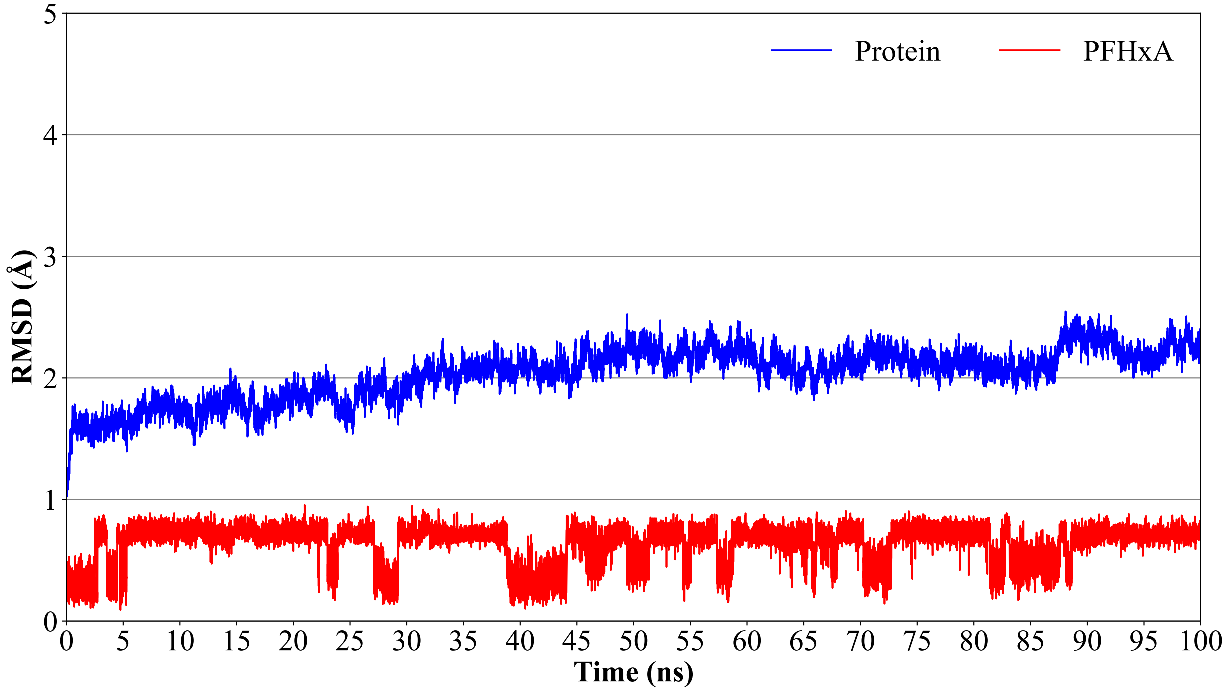

Figure S25 - Rat TTR and PFHxA RMSD.

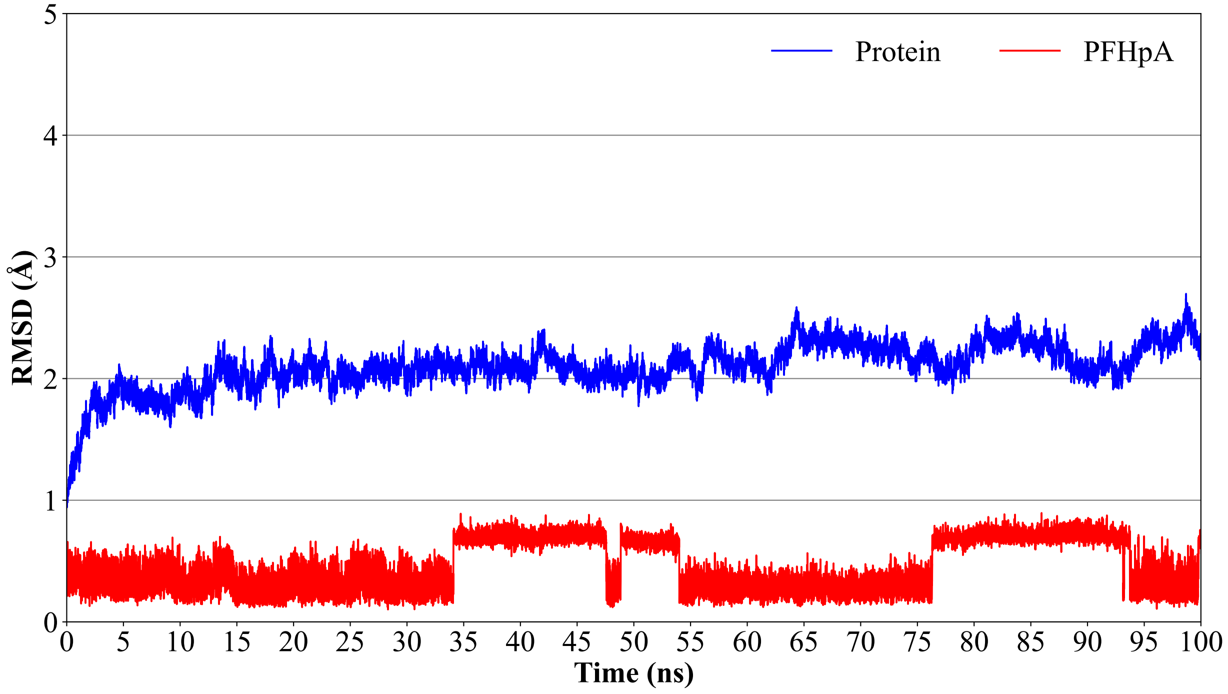

Figure S26 - Rat TTR and PFHpA RMSD.

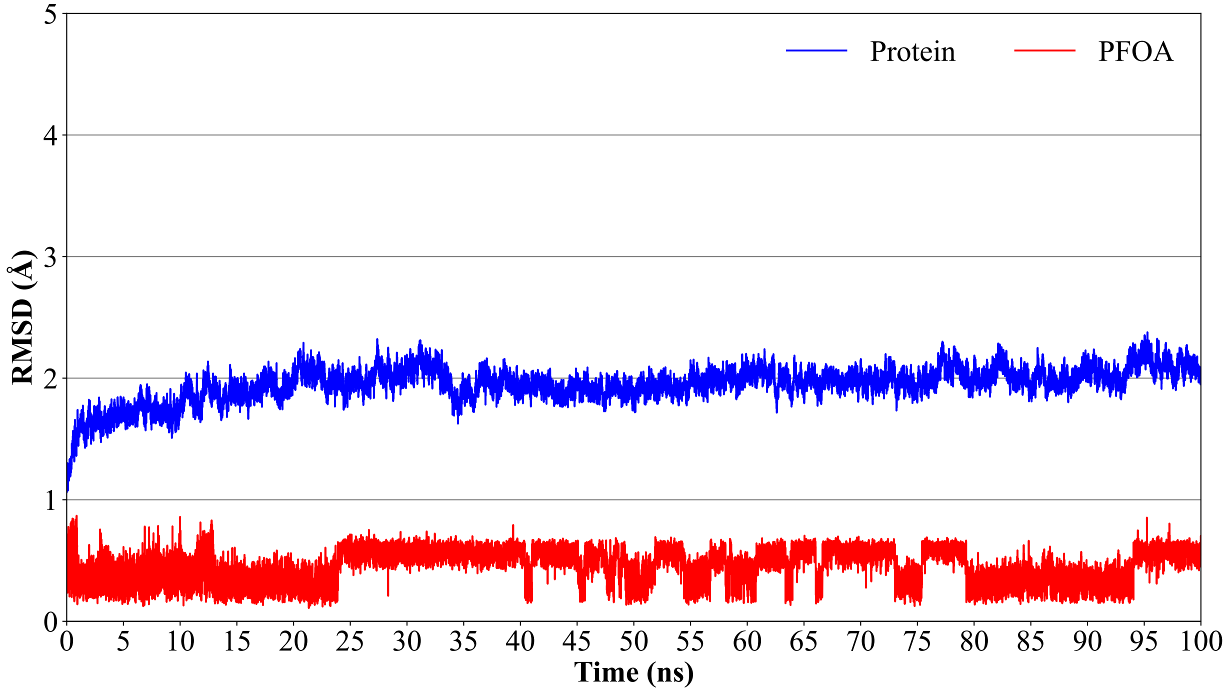

Figure S27 - Rat TTR and PFOA RMSD.

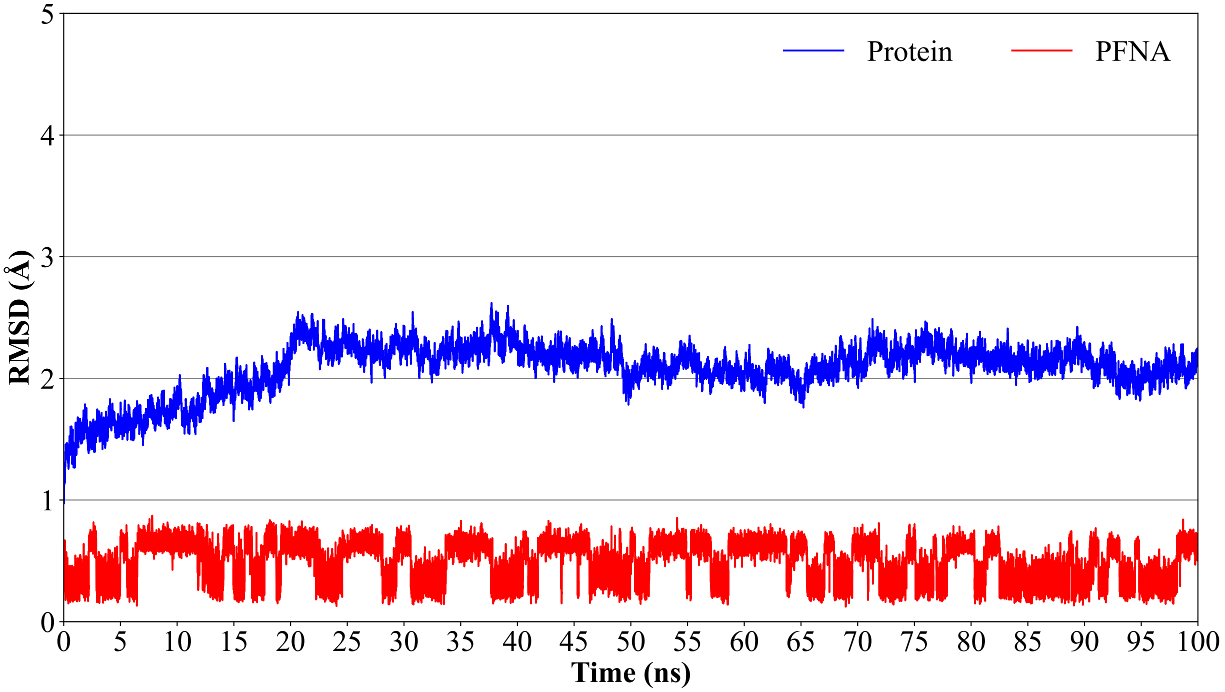

Figure S28 - Rat TTR and PFNA RMSD.

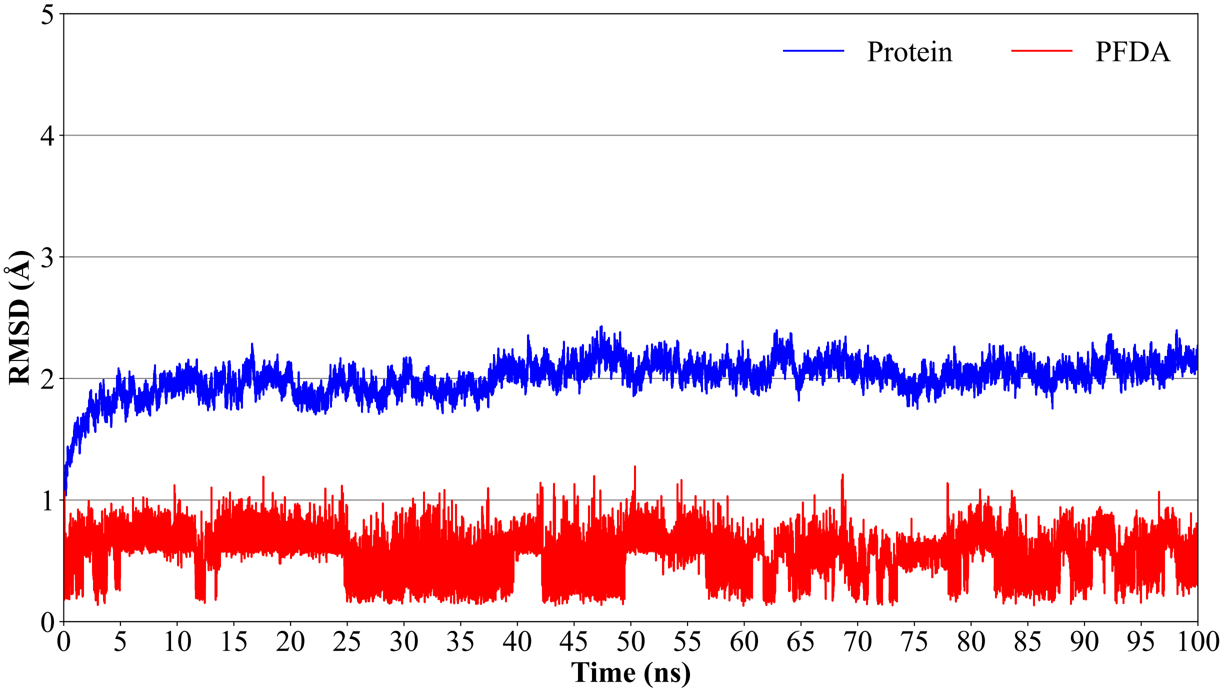

Figure S29 - Rat TTR and PFDA RMSD.

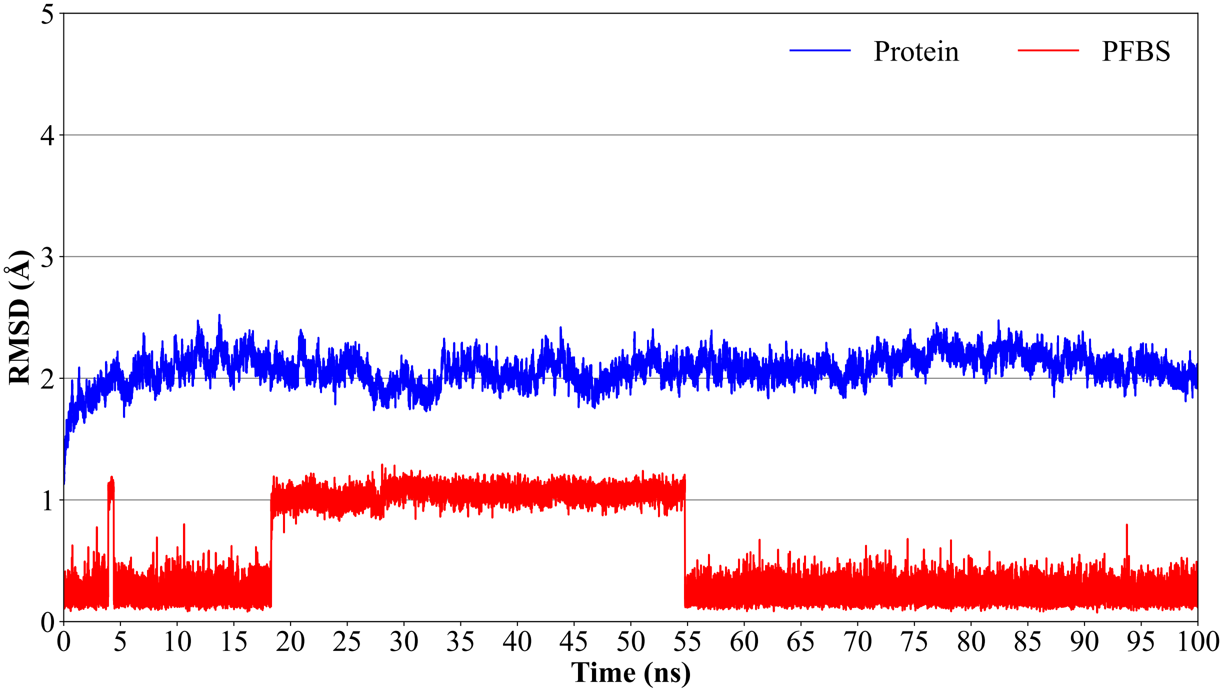

Figure S30 - Rat TTR and PFBS RMSD.

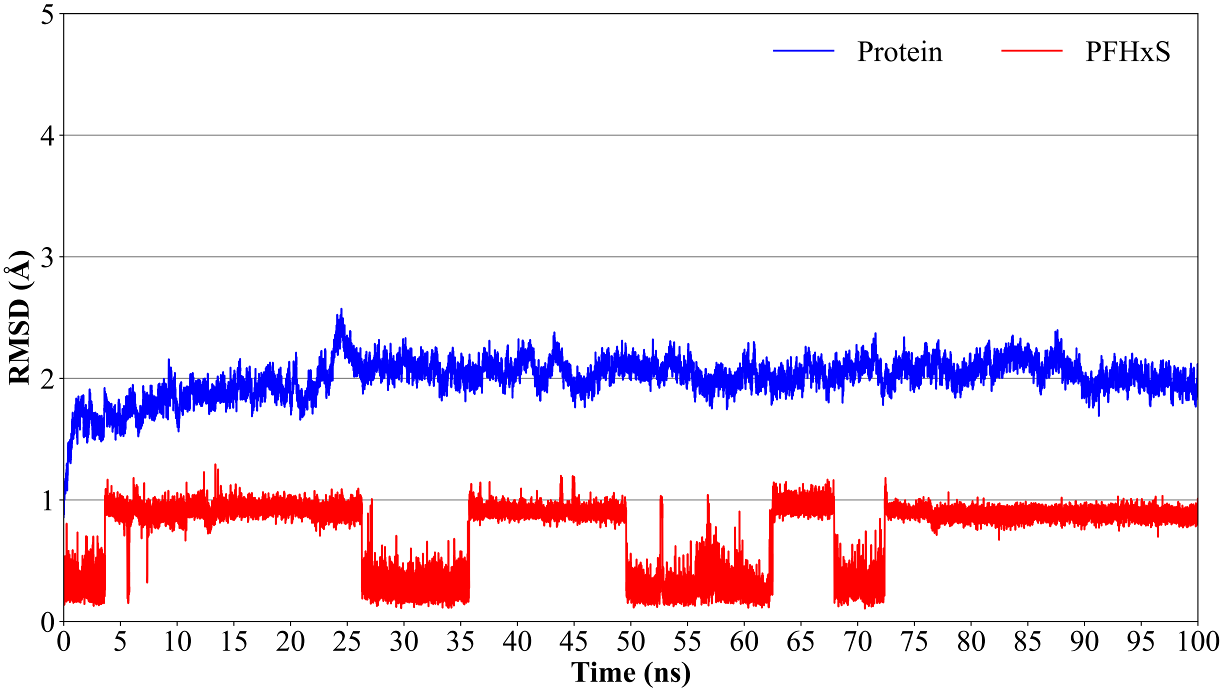

Figure S31 - Rat TTR and PFHxS RMSD.

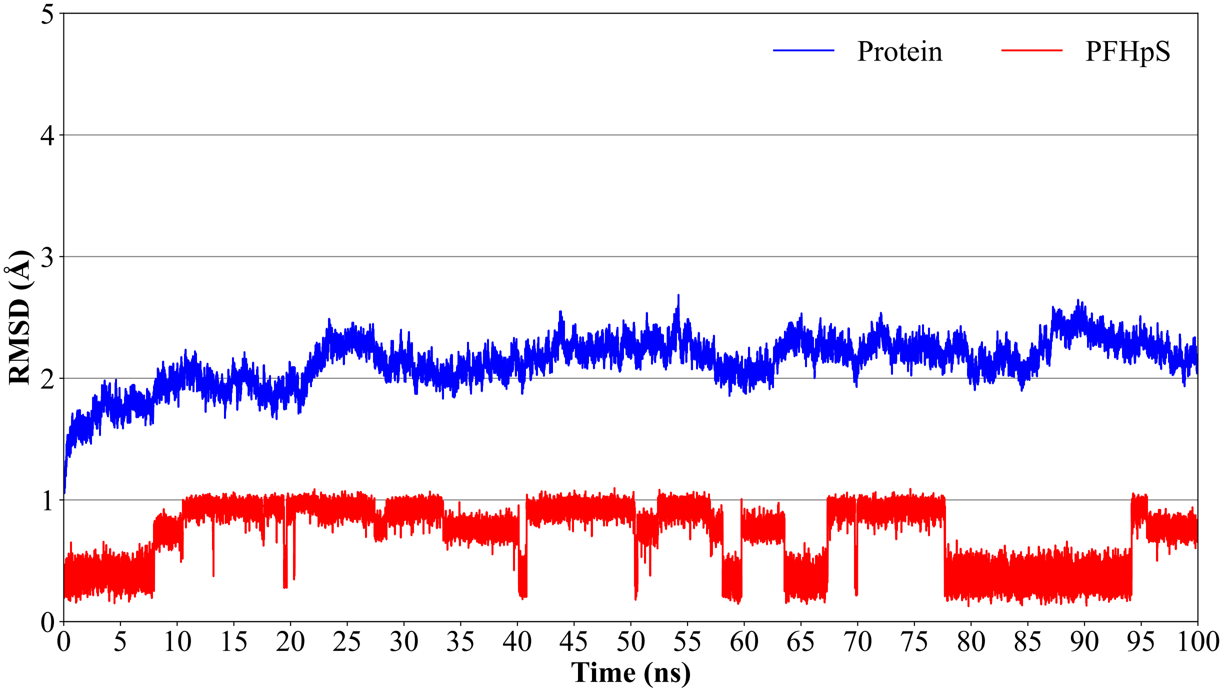

Figure S32 - Rat TTR and PFHpS RMSD.

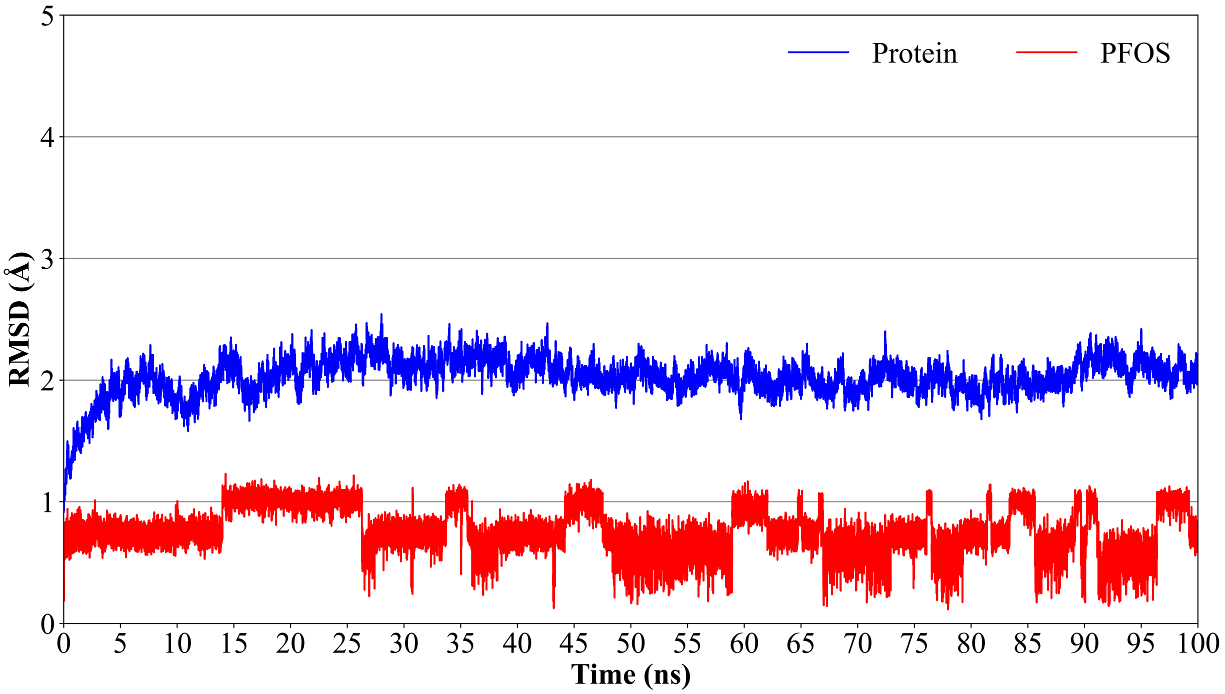

Figure S33 - Rat TTR and PFOS RMSD.

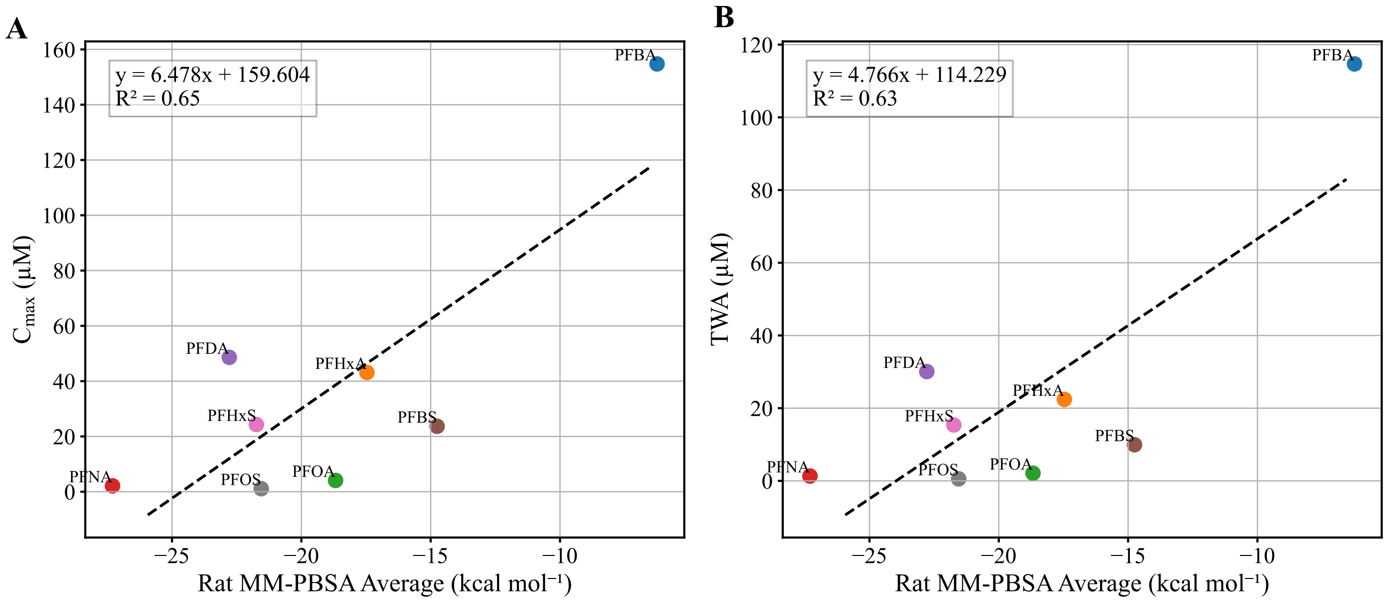

Figure S34 - PFAS/rTTR free binding energies correlate with *in vivo* points of departure (PODs) for serum free T_4 decreases in male rats. Free binding energies were calculated with molecular dynamic simulations. For toxicity studies, applied doses were converted to (A) maximum plasma concentrations (B) time averaged plasma concentrations and using PFAS-specific toxicokinetic models and converted to molar equivalents for comparison.

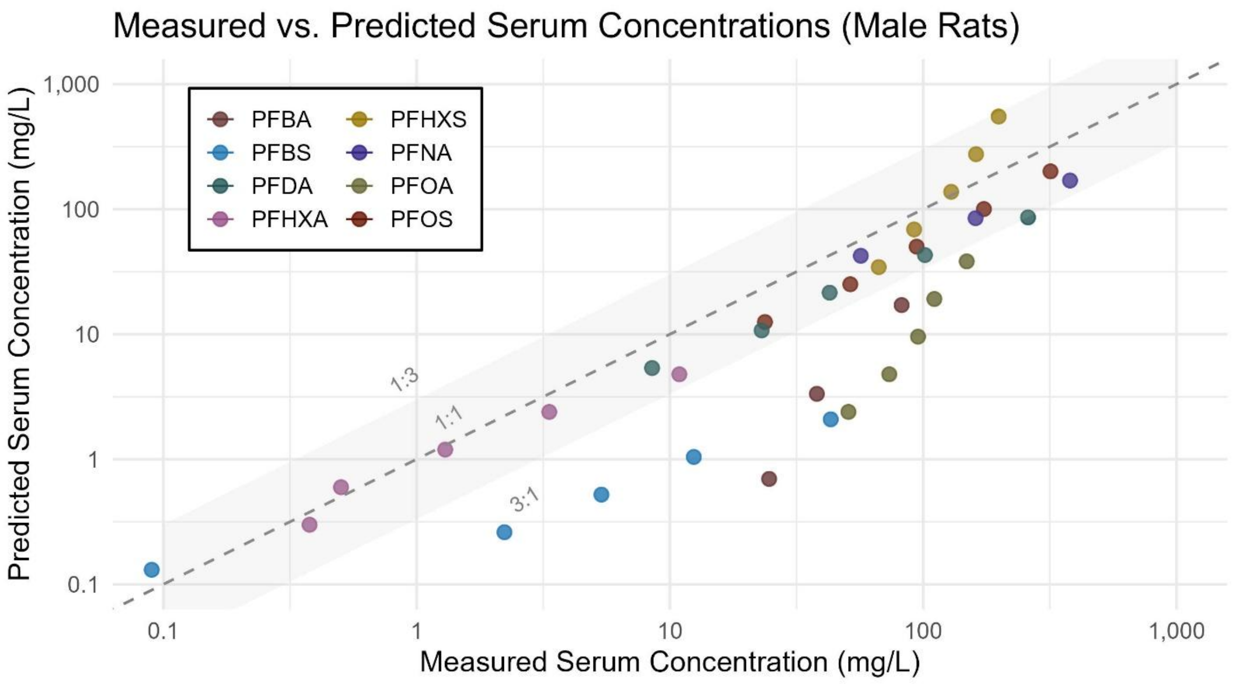

Figure S35 - Toxicokinetic modeled vs. measured serum concentrations of PFAS in male rats. Scatterplot comparing measured serum concentrations (x-axis, mg/L) to model-predicted serum concentrations from the best-performing toxicokinetic model for each PFAS (y-axis, mg/L) on $\log_{10}$–$\log_{10}$ scales. The dashed line indicates the 1:1 agreement line, and the shaded band denotes ±3-fold deviation from unity. Points represent individual study/dose observations, with colors associated with different PFAS.

References

(1) United States Environmental Protection Agency. Human Health Toxicity Value for Perfluoropropanoic Acid (CASRN 422-64-0| DTXSID8059970). **2023**.

(2) Chang, S.-C.; Das, K.; Ehresman, D. J.; Ellefson, M. E.; Gorman, G. S.; Hart, J. A.; Noker, P. E.; Tan, Y.-M.; Lieder, P. H.; Lau, C.; et al. Comparative Pharmacokinetics of Perfluorobutyrate in Rats, Mice, Monkeys, and Humans and Relevance to Human Exposure via Drinking Water. *Toxicological Sciences* **2008**, *104* (1), 40-53.

(3) United States Environmental Protection Agency. FINAL Human Health Toxicity Assessment for Perfluorooctane Sulfonic Acid (PFOS) and Related Salts. **2024**, U.S. Environmental Protection Agency Office of Water (4304T) Health and Ecological Criteria Division Washington, DC 20460, 514.

(4) Chou, W.-C.; Lin, Z. Bayesian evaluation of a physiologically based pharmacokinetic (PBPK) model for perfluorooctane sulfonate (PFOS) to characterize the interspecies uncertainty between mice, rats, monkeys, and humans: Development and performance verification. *Environment International* **2019**, *129*, 408-422.

(5) Ohmori, K.; Kudo, N.; Katayama, K.; Kawashima, Y. Comparison of the toxicokinetics between perfluorocarboxylic acids with different carbon chain length. *Toxicology* **2003**, *184* (2), 135-140.

(6) Dzierlenga, A. L.; Robinson, V. G.; Waidyanatha, S.; DeVito, M. J.; Eifrid, M. A.; Gibbs, S. T.; Granville, C. A.; and Blystone, C. R. Toxicokinetics of perfluorohexanoic acid (PFHxA), perfluorooctanoic acid (PFOA) and perfluorodecanoic acid (PFDA) in male and female Hsd:Sprague dawley SD rats following intravenous or gavage administration. *Xenobiotica* **2020**, *50* (6), 722-732.

(7) United States Department of Health and Human Services. Toxicological Profile for Perfluoroalkyls. **2021**, Agency for Toxic Substances and Disease Registry.

(8) Kim, S.-J.; Heo, S.-H.; Lee, D.-S.; Hwang, I. G.; Lee, Y.-B.; Cho, H.-Y. Gender differences in pharmacokinetics and tissue distribution of 3 perfluoroalkyl and polyfluoroalkyl substances in rats. *Food and Chemical Toxicology* **2016**, *97*, 243-255.

(9) De Silva, A. O.; Benskin, J. P.; Martin, L. J.; Arsenault, G.; McCrindle, R.; Riddell, N.; Martin, J. W.; Mabury, S. A. Disposition of perfluorinated acid isomers in sprague-dawley rats; Part 2: Subchronic dose. *Environmental Toxicology and Chemistry* **2009**, *28* (3), 555-567.

(10) Tatum-Gibbs, K.; Wambaugh, J. F.; Das, K. P.; Zehr, R. D.; Strynar, M. J.; Lindstrom, A. B.; Delinsky, A.; Lau, C. Comparative pharmacokinetics of perfluorononanoic acid in rat and mouse. *Toxicology* **2011**, *281* (1), 48-55.

(11) Sundström, M.; Chang, S.-C.; Noker, P. E.; Gorman, G. S.; Hart, J. A.; Ehresman, D. J.; Bergman, Å.; Butenhoff, J. L. Comparative pharmacokinetics of perfluorohexanesulfonate (PFHxS) in rats, mice, and monkeys. *Reproductive Toxicology* **2012**, *33* (4), 441-451.
(12) Benskin, J. P.; De Silva, A. O.; Martin, L. J.; Arsenault, G.; McCrindle, R.; Riddell, N.; Mabury, S. A.; Martin, J. W. Disposition of perfluorinated acid isomers in sprague-dawley rats; Part 1: Single dose. *Environmental Toxicology and Chemistry* **2009**, *28* (3), 542-554.
(13) D'eon Jessica, C.; Mabury Scott, A. Exploring Indirect Sources of Human Exposure to Perfluoroalkyl Carboxylates (PFCAs): Evaluating Uptake, Elimination, and Biotransformation of Polyfluoroalkyl Phosphate Esters (PAPs) in the Rat. *Environmental Health Perspectives* **2011**, *119* (3), 344-350.
(14) Weiss, J. M.; Andersson, P. L.; Lamoree, M. H.; Leonards, P. E.; van Leeuwen, S. P.; Hamers, T. Competitive binding of poly- and perfluorinated compounds to the thyroid hormone transport protein transthyretin. *Toxicol Sci* **2009**, *109* (2), 206-216.
(15) Kovarich, S.; Papa, E.; Li, J.; Gramatica, P. QSAR classification models for the screening of the endocrine-disrupting activity of perfluorinated compounds. *SAR and QSAR in Environmental Research* **2012**, *23* (3-4), 207-220.
(16) Papa, E.; Kovarich, S.; Gramatica, P. QSAR prediction of the competitive interaction of emerging halogenated pollutants with human transthyretin. *SAR and QSAR in Environmental Research* **2013**, *24* (4), 333-349.
(17) Zhang, J.; Kamstra, J. H.; Ghorbanzadeh, M.; Weiss, J. M.; Hamers, T.; Andersson, P. L. In Silico Approach To Identify Potential Thyroid Hormone Disruptors among Currently Known Dust Contaminants and Their Metabolites. *Environmental Science & Technology* **2015**, *49* (16), 10099-10107.
(18) Kar, S.; Sepulveda, M. S.; Roy, K.; Leszczynski, J. Endocrine-disrupting activity of per- and polyfluoroalkyl substances: Exploring combined approaches of ligand and structure based modeling. *Chemosphere* **2017**, *184*, 514-523.
(19) Evangelista, M.; Chirico, N.; Papa, E. In silico models for the screening of human transthyretin disruptors. *Journal of Hazardous Materials* **2024**, *480*, 136188.
(20) Ren, X.-M.; Qin, W.-P.; Cao, L.-Y.; Zhang, J.; Yang, Y.; Wan, B.; Guo, L.-H. Binding interactions of perfluoroalkyl substances with thyroid hormone transport proteins and potential toxicological implications. *Toxicology* **2016**, *366-367*, 32-42.
(21) Zhao, L.; Zhang, Z.; Su, H.; Zhang, W.; Sun, J.; Li, Y.; Teng, M. Molecular docking–QSAR–Kronecker-regularized least squares-based multiple machine learning for assessment and prediction of PFAS–protein binding interactions. *Journal of Hazardous Materials* **2025**, *492*, 138069.
(22) Sosnowska, A.; Mudlaff, M.; Mombelli, E.; Behnisch, P.; Zdybel, S.; Besselink, H.; Kuckelkorn, J.; Bulawska, N.; Kepka, K.; Kowalska, D.; et al. Identification of new PFAS for severe interference with thyroid hormone transport: A combined in vitro/silico approach. *Journal of Hazardous Materials* **2025**, *491*.
(23) Evangelista, M.; Chirico, N.; Papa, E. New QSAR Models to Predict Human Transthyretin Disruption by Per- and Polyfluoroalkyl Substances (PFAS): Development and Application. *Toxics* **2025**, *13* (7), 590.
(24) Degitz, S. J.; Olker, J. H.; Denny, J. S.; Degoey, P. P.; Hartig, P. C.; Cardon, M. C.; Eytcheson, S. A.; Haselman, J. T.; Mayasich, S. A.; Hornung, M. W. In vitro screening of per- and polyfluorinated substances (PFAS) for interference with seven thyroid hormone system targets across nine assays. *Toxicology in Vitro* **2024**, *95*, 105762.

(25) Makarov, D. M.; Ksenofontov, A. A. B., Yury A. Consensus Modeling for Predicting Chemical Binding to Transthyretin as the Winning Solution of the Tox24 Challenge. *Chemical Research in Toxicology* **2025**, *38* (3), 392-399.
(26) Richard, A. M.; Huang, R.; Waidyanatha, S.; Shinn, P.; Collins, B. J.; Thillainadarajah, I.; Grulke, C. M.; Williams, A. J.; Lougee, R. R.; Judson, R. S.; et al. The Tox21 10K Compound Library: Collaborative Chemistry Advancing Toxicology. *Chemical Research in Toxicology* **2021**, *34* (2), 189-216.
(27) Richard, A. M.; Tao, D.; LeClair, C. A.; Leister, W.; Tretyakov, K. V.; White, E. V.; Lewis, K. C.; Sefler, A.; Shinn, P.; Collins, B. J.; et al. Analytical Quality Evaluation of the Tox21 Compound Library. *Chemical Research in Toxicology* **2025**, *38* (1), 15-41.
(28) Butenhoff, J. L.; Bjork, J. A.; Chang, S. C.; Ehresman, D. J.; Parker, G. A.; Das, K.; Lau, C.; Lieder, P. H.; van Otterdijk, F. M.; Wallace, K. B. Toxicological evaluation of ammonium perfluorobutyrate in rats: twenty-eight-day and ninety-day oral gavage studies. *Reproductive Toxicology* **2012**, *33* (4), 513-530.
(29) National Toxicology Program. NTP Technical Report on the Toxicity Studies of Perfluoroalkyl Sulfonates (Perfluorobutane Sulfonic Acid, Perfluorohexane Sulfonate Potassium Salt, and Perfluorooctane Sulfonic Acid) Administstered by Gavage to Sprague Dawley (Hsd:Sprague Dawley SD) Rats (Revised). Toxicity Report Series 96. **2022**.
(30) National Toxicology Program. NTP Technical Report on the Toxicity Studies of Perfluoroalkyl Carboxylates (Perfluorohexanoic Acid, Perfluorooctanoic Acid, Perfluorononanoic Acid, and Perfluorodecanoic Acid) Administered by Gavage to Sprague Dawley (Hsd: Sprague Dawley SD) Rats (Revised). Toxicity Report Series 97. **2022**.
(31) Hornak, V.; Abel, R.; Okur, A.; Strockbine, B.; Roitberg, A.; Simmerling, C. Comparison of multiple Amber force fields and development of improved protein backbone parameters. *Proteins: Structure, Function, and Bioinformatics* **2006**, *65* (3), 712-725.
(32) Hoffmann, R. An Extended Hückel Theory. I. Hydrocarbons. *The Journal of Chemical Physics* **1963**, *39* (6), 1397-1412.
(33) Corbeil, C. R.; Williams, C. I.; Labute, P. Variability in docking success rates due to dataset preparation. *Journal of Computer-Aided Molecular Design* **2012**, *26* (6), 775-786.
(34) D.A. Case, H. M. A., K. Belfon, I.Y. Ben-Shalom, J.T. Berryman, S.R. Brozell, D.S. Cerutti, T.E.; Cheatham, I., G.A. Cisneros, V.W.D. Cruzeiro, T.A. Darden, R.E. Duke, G. Giambasu, M.K. Gilson, H.; Gohlke, A. W. G., R. Harris, S. Izadi, S.A. Izmailov, K. Kasavajhala, M.C. Kaymak, E. King, A. Kovalenko, T. Kurtzman, T.S. Lee, S. LeGrand, P. Li, C. Lin, J. Liu, T. Luchko, R. Luo, M. Machado, V.; Man, M. M., K.M. Merz, Y. Miao, O. Mikhailovskii, G. Monard, H. Nguyen, K.A. O'Hearn, A.; Onufriev, F. P., S. Pantano, R. Qi, A. Rahnamoun, D.R. Roe, A. Roitberg, C. Sagui, S. Schott-Verdugo,; A. Shajan, J. S., C.L. Simmerling, N.R. Skrynnikov, J. Smith, J. Swails, R.C. Walker, J. Wang, J. Wang,; H. Wei, R. M. W., X. Wu, Y. Xiong, Y. Xue, D.M. York, S. Zhao, and P.A. Kollman. Amber2022. *University of California, San Francisco* **2022**.
(35) He, X.; Man, V. H.; Yang, W.; Lee, T. S.; Wang, J. A fast and high-quality charge model for the next generation general AMBER force field. *The Journal of Chemical Physics* **2020**, *153* (11), 114502.
(36) Wang, J.; Wolf, R. M.; Caldwell, J. W.; Kollman, P. A.; Case, D. A. Development and testing of a general amber force field. *Journal of Computational Chemistry* **2004**, *25* (9), 1157-1174.

(37) Maier, J. A.; Martinez, C.; Kasavajhala, K.; Wickstrom, L.; Hauser, K. E.; Simmerling, C. ff14SB: Improving the Accuracy of Protein Side Chain and Backbone Parameters from ff99SB. *Journal of Chemical Theory and Computation* **2015**, *11* (8), 3696-3713.
(38) Joung, I. S.; Cheatham, T. E., III. Determination of Alkali and Halide Monovalent Ion Parameters for Use in Explicitly Solvated Biomolecular Simulations. *The Journal of Physical Chemistry B* **2008**, *112* (30), 9020-9041.
(39) Ryckaert, J.-P.; Ciccotti, G.; Berendsen, H. J. C. Numerical integration of the cartesian equations of motion of a system with constraints: molecular dynamics of n-alkanes. *Journal of Computational Physics* **1977**, *23* (3), 327-341.
(40) Huang, M.; Dzierlenga, A.; Robinson, V.; Waidyanatha, S.; DeVito, M.; Eifrid, M.; Granville, C.; Gibbs, S.; Blystone, C. Toxicokinetics of perfluorobutane sulfonate (PFBS), perfluorohexane-1-sulphonic acid (PFHxS), and perfluorooctane sulfonic acid (PFOS) in male and female Hsd: Sprague Dawley SD rats after intravenous and gavage administration. *Toxicology reports* **2019**, *6*, 645-655.
(41) Dzierlenga, A. L.; Robinson, V. G.; Waidyanatha, S.; DeVito, M. J.; Eifrid, M. A.; Gibbs, S. T.; Granville, C. A.; Blystone, C. R. Toxicokinetics of perfluorohexanoic acid (PFHxA), perfluorooctanoic acid (PFOA) and perfluorodecanoic acid (PFDA) in male and female Hsd: Sprague dawley SD rats following intravenous or gavage administration. *Xenobiotica* **2020**, *50* (6), 722-732.
(42) Gomis, M. I.; Vestergren, R.; Borg, D.; Cousins, I. T. Comparing the toxic potency in vivo of long-chain perfluoroalkyl acids and fluorinated alternatives. *Environment International* **2018**, *113*, 1-9.
